# Pseudogene-gene functional networks are prognostic of patient survival in breast cancer

**DOI:** 10.1186/s12920-020-0687-0

**Published:** 2020-04-03

**Authors:** Sasha Smerekanych, Travis S. Johnson, Kun Huang, Yan Zhang

**Affiliations:** 1 Kenyon College, Gambier, OH 43022 USA; 2 Department of Biomedical Informatics, College of Medicine, The Ohio State University, Columbus, OH 43210 USA; 3 Department of Medicine, School of Medicine, Indiana University, Indianapolis, IN 46202 USA; 4 Regenstrief Institute, Indiana University, Indianapolis, IN 46202 USA; 5 The Ohio State University Comprehensive Cancer Center (OSUCCC – James), Columbus, OH 43210 USA

**Keywords:** Data integration, Breast cancer, Survival prognosis, Pseudogenes, Non-coding RNAs, RNA-Seq, Network analysis, Cox regression, Database

## Abstract

**Background:**

Given the vast range of molecular mechanisms giving rise to breast cancer, it is unlikely universal cures exist. However, by providing a more precise prognosis for breast cancer patients through integrative models, treatments can become more individualized, resulting in more successful outcomes. Specifically, we combine gene expression, pseudogene expression, miRNA expression, clinical factors, and pseudogene-gene functional networks to generate these models for breast cancer prognostics. Establishing a LASSO-generated molecular gene signature revealed that the increased expression of genes STXBP5, GALP and LOC387646 indicate a poor prognosis for a breast cancer patient. We also found that increased CTSLP8 and RPS10P20 and decreased HLA-K pseudogene expression indicate poor prognosis for a patient. Perhaps most importantly we identified a pseudogene-gene interaction, GPS2-GPS2P1 (improved prognosis) that is prognostic where neither the gene nor pseudogene alone is prognostic of survival. Besides, miR-3923 was predicted to target GPS2 using miRanda, PicTar, and TargetScan, which imply modules of gene-pseudogene-miRNAs that are potentially functionally related to patient survival.

**Results:**

In our LASSO-based model, we take into account features including pseudogenes, genes and candidate pseudogene-gene interactions. Key biomarkers were identified from the features. The identification of key biomarkers in combination with significant clinical factors (such as stage and radiation therapy status) should be considered as well, enabling a specific prognostic prediction and future treatment plan for an individual patient. Here we used our PseudoFuN web application to identify the candidate pseudogene-gene interactions as candidate features in our integrative models. We further identified potential miRNAs targeting those features in our models using PseudoFuN as well. From this study, we present an interpretable survival model based on LASSO and decision trees, we also provide a novel feature set which includes pseudogene-gene interaction terms that have been ignored by previous prognostic models. We find that some interaction terms for pseudogenes and genes are significantly prognostic of survival. These interactions are cross-over interactions, where the impact of the gene expression on survival changes with pseudogene expression and vice versa. These may imply more complicated regulation mechanisms than previously understood.

**Conclusions:**

We recommend these novel feature sets be considered when training other types of prognostic models as well, which may provide more comprehensive insights into personalized treatment decisions.

## Introduction

Breast cancer is the second most common cancer among women [1] and the second-leading cause of cancer death [2], indicating a widespread, detrimental effect on the population as a whole. The high death-rate is due to the complexity of the disease -- each case is unique, with different clinical and molecular characteristics which makes a single “silver bullet” treatment unlikely [3]. These considerations make personalized treatments a more promising route. Indeed, we see that individuals with the HER2-positive breast cancer subtype are treated with drugs designed to specifically target the surface HER2/neu proteins, such as Trastuzumab [4]. ER/PR-positive patients are treated with hormone therapy, as they are hormone sensitive and tend to have better outcomes. In contrast, patients with triple-negative breast cancer (TNBC) would not respond to such hormonal treatments as their cancer is not proliferated by hormones and tends to be more aggressive [5]. Current TNBC treatments include p53 (e.g., using drug taxanes) and cell proliferation targeted therapies, given the higher response rates using chemotherapy than in other types of breast cancer [5]. These past successes show personalized treatment plans with a greater degree of efficacy must account for a comprehensive understanding of that specific tumor and tumor microenvironment.

Cancerous states arise from mutations and tissue dysregulation, and these alterations are usually associated with abnormal gene expressions. For example, in aggressive B-cell lymphoma tumors resulting from an alteration in the p53 tumor suppressor, an overexpression of p14ARF was observed, suggesting its potential as a cell cycle regulator and a marker of high tumor severity [6]. Differential expression of specific genes have been found to lead to tumor development, such as ITGA11 and JAB1, which have been discovered to be biomarkers in breast cancer [7]. High levels of STAT1 have been detected in a subset of breast cancers, resulting in the induction of known IFN-regulated genes [8]. It is therefore possible to infer the underlying mechanism of specific cancer types through the measurement of mRNA expression in RNA-Seq and microarrays, leading to personalized medicine assays such as OncotypeDx [9, 10].

Expression of miRNAs, a class of short non-coding RNA sequences, can also play a role in tumorigenesis by mediating gene expression [11]. These sequences are also very common, with over 2500 sequences annotated in the human genome [7]. Already multiple associations with diseases and targetability by antisense inhibition make them a promising therapeutic target [12]. It has been discovered that about half of the known human miRNAs are related to cancer, influencing gene expression levels, which in turn impacts all aspects of the disease, from progression to remission [13]. Given that miRNA dysregulation impacts their regulatory function over cell proliferation, adhesion, and migration processes [2], these sequences thus have the potential to be significant biomarkers in breast cancer. This idea has been explored in previous studies, with findings indicating that miR-320a, miR-361-5p, and miR-21-5p may lead to a higher survival rate in breast cancer [2]. A correlation has been discovered between poor survival rates and decreased miR-200b expression levels, with an upregulation in miR-200 leading to improved colonization and proliferation suppression in cancer cell lines [14]. Expression levels of miRNAs should therefore be considered when predicting prognosis and establishing a treatment plan. Gene regulatory relationships are not limited to gene-miRNA interactions and in fact pseudogene regulation has been observed as well [15].

Pseudogenes have been shown to affect the regulatory mechanisms in pan-cancer studies [16]. Furthermore, pseudogene expression can be used as a novel marker to stratify patient subtypes in multiple cancer types [17] which implies these regulatory relationships should be taken into account in cancer survival prognosis. More specifically, individual pseudogenes have been implicated and externally validated as regulators of their parent genes [15]. One such example PTEN (gene)-PTENP1 (pseudogene) interaction has been implicated in prostate cancer due to the competing endogenous RNA (ceRNA) network they participate in^18^. There are a multitude of other regulatory pseudogene-gene relationships that have been validated including FTH1-FTH1PX (X denotes multiple pseudogenes) in prostate cancer [18], SUMO1-SUMO1P3 in gastric cancer [19], ATP8A2-ATP8A2Ψ in breast cancer [16]. These examples provide compelling evidence that pseudogene-gene interactions can be directly used as prognostic features in human cancer but to fully utilize these relationships it is important to identify candidate interactions.

Candidate interactions can be uncovered using direct 1:1 pseudogene to gene parent mappings; however, these interactions do not take into account the wider array of sequence homology relationships that may cause regulation. For this purpose, we use the idea of pseudogene-gene networks, which are networks of pseudogenes and genes that have high sequence homology and as a result may be functionally related [20]. These functional relationships are taken from the Pseudogene Functional Network (PseudoFuN) database [21] so that pseudogenes and genes could be mapped back to prospective regulatory partners.

Given the recent technological rise in next- and third-generation sequencing, the establishment of large genomic databases and effective computational and statistical methods enabled the potential to uncover new findings on cancer biology through bioinformatic analysis. One database, The Cancer Genome Atlas (TCGA) [22], contains genomic and clinical data on thousands of individuals that suffered from an array of cancer types. Utilizing their breast invasive carcinoma (BRCA) datasets, we can determine the prognosis of an individual based on clinical, molecular features, and molecular interactions and generate efficient, interpretable diagrams as a novel prognostic tool as well as to guide for treatment decisions.

Distilling the large numbers of candidate features down to usable subsets is a common problem in biomedical data analytics [23] which was first manifested as the “curse of dimensionality” as early as 1957 [24]. Univariate feature selection is an approach addressing the “curse of dimensionality” common in biomedical sciences because it allows the features to be ranked on their individual ability to predict the dependent variable [25, 26]. More specifically these methods have been applied directly with univariate Cox proportional hazards models [27]. Univariate feature selection has a long history of use [28] due to its computational efficiency and ability to rank genes based on their individual predictive power [25]. For these reasons, we first apply the univariate Cox proportional hazards models to filter down the pool of candidate features, followed by multivariate LASSO-Cox models for feature selection.

In order to efficiently interpret an individual’s prognosis, decision tree models will be generated based on the features selected by the above multivariate models considering both molecular and clinical factors, as this approach has been known to have high prediction accuracy [29]. This specific form of modeling is capable of handling common difficulties in bioinformatic datasets, such as their high dimensional data from large sample sizes. This model’s popularity also stems from its straightforward interpretation. The implemented algorithm follows a feature selection procedure, sorting out the most significant factors that influence the outcome, which in this case is a patient’s overall survival (OS). In a decision tree, the particular feature, or factor, then splits the samples into daughter nodes, followed by recursive repetition of the whole process into further nodes until the desired stopping criterion is reached [29, 30]. Ultimately, the most influential factors across all molecular and clinical data will be integrated as nodes in an interpretable tree, leading to the most probable survival curve for a specific individual given the values of molecular and clinical features. This enables the medical team to construct an appropriate treatment plan given the patient’s precise prognosis.

## Materials and methods

### Clinical and molecular data acquisition and alignment

**Fig. 1:**
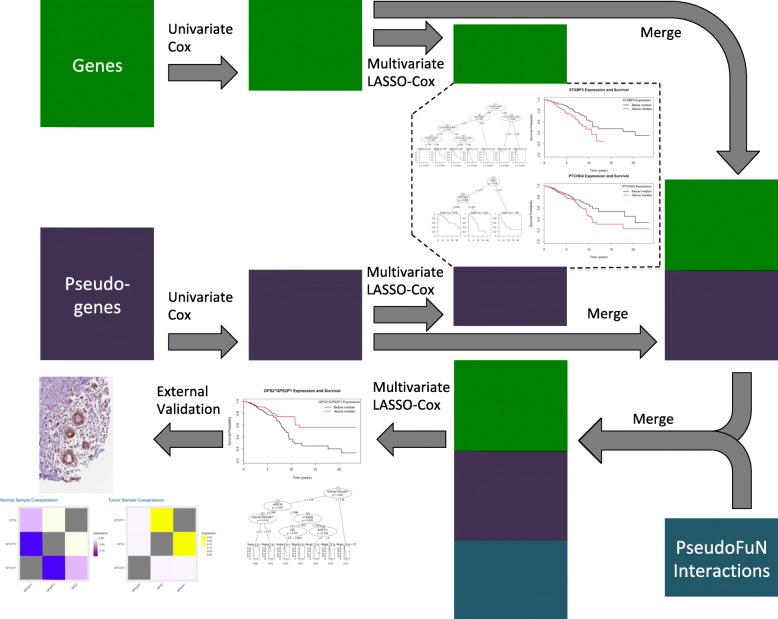
General workflow identifying genes, pseudogenes, and gene-pseudogene functional relationships that are prognostic of breast cancer survival. The workflow shows how the datasets were reduced down at each step and where new datasets were added. The rectangular boxes represent the gene expression matrices at each stage where the number of pseudogenes, genes, or interactions are reduced

We acquired the clinical data associated with BRCA samples, including the patients’ days to death, days to last follow up, vital status, pathological stage, histological type, number of lymph nodes, gender and race from Broad GDAC Firehose portal [22]. The pseudogene expression was downloaded from dreamBase [31]. The data was filtered to include only patient IDs present in the RNA expression datasets (mRNA, miRNA, and pseudogene expression). We compiled the overall survival time by assessing the vital status, utilizing the days to death value if the patient is deceased, or the days to last follow up value if the patient is alive. A Kaplan-Meier plot was used to visualize the overall survival data, comparing the proportion of patients that were still alive versus the survival time. To compare the survival data with RNA expression, the clinical and expression data were formatted, maintaining only matching patients samples and removing any *NAs* that were present. The normalized expression data (e.g., RPKM) was converted by a log2 transformation. The final processed gene mRNA and clinical datasets contained 765 patient samples and 20,532 genes. The processed pseudogene RNA and clinical datasets contained 1077 patient samples and 7146 pseudogenes. There were 762 patients that were contained in both the gene and pseudogene expression matrices. The general workflow of our analysis is summarized in Fig. 1.

### Screening for significantly expressed genes and pseudogenes

The screening was performed on the gene expression matrix and the pseudogene expression matrix independently using univariate Cox model for each gene or pseudogene, so that the pool of candidate features was selected in each dataset separately. A feature indicates one gene or pseudogene in the full gene set (*G*) or pseudogene set (*P*) depending on the expression matrix that is being used in this section. With the intention of deriving a molecular signature of genes that indicate a patient’s prognosis based on their expression values, we first split two-thirds of the data into a training set (67%) and the remainder into a test set (33%), then constructed a univariate Cox model to test the significance of each feature (gene/pseudogene). For each feature, a univariate Cox model was fit on the training set and the *p*-value of score test calculated on the same training set. (The test set will be used in testing LASSO-Cox regression models.) These *p*-values were corrected using Benjamini-Hochberg procedure (BH-FDR). All features with a BH-FDR below 0.05 were used as our top features (i.e., pool of candidate features) in the following analysis. Specifically, for each gene or pseudogene (*j* ∈ {*G*, *P*}) across all patients (*i*), the following model was fit:

$$ h\left(t|{X}_{i,j}\right)={h}_0(t)\exp \left({X}_{i,j}{\beta}_j\right), $$

where *t* is the time point, *X* is the expression matrix, and *β* are the coefficients of the model. *h*_0_(*t*) is the baseline hazard function, and *h*(*t*| *X*_*i*, *j*_) is the hazard function for individual *i* at time *t* using the *j* th expression profile. The hazard ratio was calculated using Cox regression and log-rank test was performed to see if the hazard ratio was significantly different than 1. The preselected gene set (*g*) and pseudogene set (*p*) were used for further analysis.

### LASSO-Cox regression on top features from screening

In order to enhance prediction accuracy, we utilized a multivariate LASSO-Cox model [32] on our training set, and test its predictive performance on the test set. Starting with the top univariate features from our univariate Cox models, the most precise gene signature impacting survival is determined by screening out features with a zero coefficient. A Kaplan-Meier plot was constructed for each selected feature, including a summary that revealed the specific *p*-values. These p-values enabled us to determine which features were the most significant biomarkers for determining prognosis. Specifically, using all of the preselected genes (*g* ⊆ *G*) and pseudogenes (*p* ⊆ *P*) the following models were fit:

$$ h\left(t|{X}_{i,g}\right)={h}_0\left(\mathrm{t}\right)\exp \left({X}_{i,g}{\beta}_g\right)+{\left\Vert {\beta}_g\right\Vert}_1 $$

and

$$ h\left(t|{X}_{i,p}\right)={h}_0\left(\mathrm{t}\right)\exp \left({X}_{i,p}{\beta}_p\right)+{\left\Vert {\beta}_p\right\Vert}_1. $$

### Generating interpretable prognostic models

Using the *ctree* function from the R *partykit* package [33], tree-structured regression models on survival were constructed taking into account LASSO-generated molecular signature and clinical variables (such as ethnicity, race, gender, histological type, pathologic stage, radiation therapy, race and so on). These tree-structured models were used to interpret how the given factors influences one’s survival prognosis in a concise manner.

### Integrating gene and pseudogene expression using pseudogene functional networks

The top genes and pseudogenes that were identified in the earlier analysis were used as features in the subsequent pseudogene-gene integrative models. The pseudogene-gene functional network edge file was obtained from the BlastDB database (a flavor of the pseudogene-gene family database) in the PseudoFuN website [21]. The edge between a gene and a pseudogene indicates that the gene and pseudogene were contained in the same homology network and were used to identify the possible interactions between genes and pseudogenes in the model. For each gene, the corresponding pseudogene(s) were identified using the BlastDB and the gene-pseudogene interaction terms (*a*) were added to the model. For each pseudogene, the corresponding gene(s) were identified using the BlastDB and the pseudogene-gene interaction terms were added to the model. A LASSO-Cox model was trained on the genes, pseudogenes, and interaction terms (*a*). The non-zero interaction terms were studied using median expression stratified Kaplan-Meier curves. Specifically, models were trained on all of the retained genes, pseudogenes, and associated interactions:

$$ h\left(t|{X}_{i,\left\{g,p,a\right\}}\right)={h}_0\left(\mathrm{t}\right)\exp \left({X}_{i,\left\{g,p,a\right\}}{\beta}_{\left\{g,p,a\right\}}\right)+{\left\Vert {\beta}_{\left\{g,p,a\right\}}\right\Vert}_1. $$

The features (genes, pseudogenes, interactions) with nonzero interactions were retained for further analysis. For each interaction term with nonzero coefficient, if the main effects corresponding to that interaction had a zero coefficient they were retained to improve interpretability producing the final feature set (*l* ⊆ {*g*, *p*, *a*}) of genes, pseudogenes, and interactions and the following model was fit:

$$ h\left(t|{X}_{i,l}\right)={h}_0\left(\mathrm{t}\right)\exp \left({X}_{i,l}{\beta}_l\right). $$

### External validation of features identified by interpretable models

Once the final models were fit and the final feature set was identified, it was important to externally validate the features that were available outside of the TCGA dataset. To perform this validation we used a Swedish cohort of 3069 patients (GSE81538). For each gene or pseudogene in the final Cox model, a Wald test was calculated using the median feature expression to stratify the patients into high and low risk groups. The Swedish cohort was stratified into 28 smaller cohorts to explore patient subtyping in detail by PAM50 status, receptor status (estrogen receptor, progesterone receptor, and HER2), and age (non-senior vs. all). These groups help us to further identify what patient cohorts are identified by what features in the multiple linear regression Cox model.

## Results

### miRNAs alone not directly prognostic of breast cancer survival in our dataset

To assess whether expression of individual miRNAs bestows prognostic capabilities based on the provided clinical factors, we used univariate Cox regression models followed by a multivariate LASSO-Cox model consisting of both expression and clinical data. None of the miRNAs were identified as significant in our dataset, excluding miRNA data from our consideration for features in our integrative model.

### Gene subsets prognostic of breast Cancer survival

**Fig. 2:**
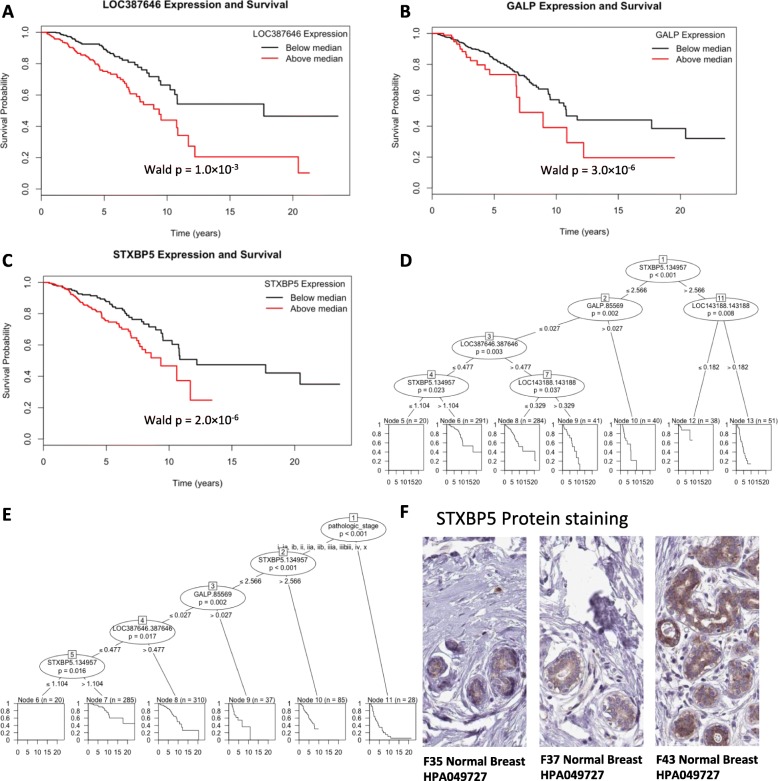
Prognostic gene models in BRCA. **a** Kaplan-Meier survival plot for LOC387646. **b** Kaplan-Meier survival plot for GALP. **c** Kaplan-Meier survival plot for STXBP5. D) Decision tree based on only gene expression data excluding clinical variables. **e** Decision tree based on gene expression and clinical variables. **f** Histology staining from the Human Protein Atlas [34] for STXBP5 in normal tissue (includes: gender, age, tissue type, and antibody used in bold on the histology slide). For example, F35 means a 35-year-old female, and HPA049727 is the antibody. The tissue staining is used to show that the specific protein product is present in normal tissue. Thus regulating that gene (STXBP5) at the mRNA level might affect the phenotype because the protein product is present and can be a regulation target

To assess whether expression of individual mRNAs bestows prognostic capabilities based on the provided clinical factors, a multivariate LASSO-Cox model, consisting of both expression and clinical data, was constructed (details follow the description in Materials and Methods). Of the 20,532 gene mRNAs screened by univariate Cox models, 1007 were significant at the unadjusted threshold of *p*-value < 0.05 and 27 were deemed statistically significant (BH-FDR < 0.05). To further evaluate the prognostic properties of mRNA expression, a multivariate LASSO-Cox model was developed with the top univariate genes previously found through the univariate Cox models, indicating a molecular signature with 4 key significantly expressed genes: LOC387646, GALP, STXBP5, and LOC143188. Kaplan-Meier curves were generated for the 4 most promising genes to further explore their clinical importance, comparing the specific gene expression with the median risk-score. High levels of LOC387646 (Fig. 2a, Wald *p*-value = 1.0 × 10^− 03^), GALP (Fig. 2b, Wald p-value = 3.0 × 10^− 06^), STXBP5 (Fig. 2c, Wald *p*-value = 2.0 × 10^− 06^), and LOC143188 (Additional file 1 R compilation output, Wald p-value = 4.0 × 10^− 07^) were significantly associated with reduced survival times.

In order to assess the likely outcome of a specific patient, not only does the molecular expression (mRNA, miRNA) need to be evaluated, but also clinical variables, such as ethnicity, race, gender, cancer stage, type, and radiation therapy status, need to be factored in as well for more reliable, individualized prognosis. The target model, based on the data from 769 patients, is summarized and visualized. The *ctree* function is used to construct a tree that displays the survival curves given the set of clinical and/or molecular factors. The first tree generated included only gene expression data, creating an outline using genes LOC387646, GALP, and STXBP5 (Fig. 2d). Using only these genes, patients could be stratified into 7 groups based on survival. The following tree was constructed with both clinical and molecular data under consideration (Fig. 2e). The tree started with the assessment of pathologic stage, followed by the expression level of STXBP5. A patient with the highest likelihood of survival would fall under Node 8 (Fig. 2e, Node 8 labeled in bottom of Fig. 2e), and patients could be stratified into 6 groups based on survival. Aside from the ability of these genes to predict survival prognosis we find that STXBP5 protein is detectable at medium levels in glandular cells from normal breast tissue samples (Fig. 2f). Since the protein is present in normal breast tissue, STXBP5 mRNA dysregulation could affect syntaxin binding protein 5 (STXBP5) protein levels in BRCA tumor tissue.

### Prognostic ability of pseudogenes in breast cancer

**Fig. 3:**
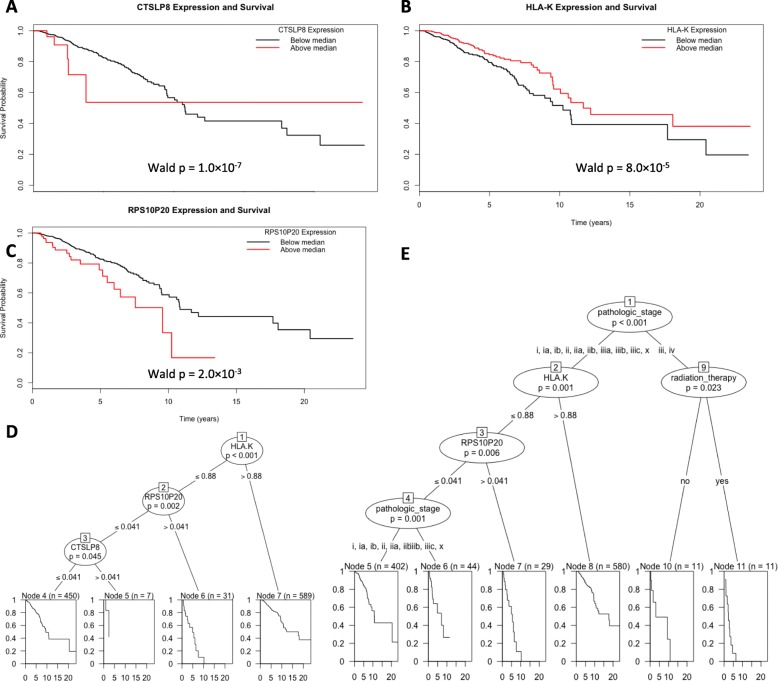
Prognostic pseudogene models in BRCA. **a** Kaplan-Meier survival plot for PGK1. **b** Kaplan-Meier survival plot for PTCHD4. **c** Decision tree based on only pseudogene expression data without clinical variables. **d** Decision tree based on pseudogene expression and clinical variables

Of the 7146 pseudogenes screened using univariate Cox models, 323 pseudogenes were significant at the unadjusted threshold of *p*-value < 0.05 and 14 pseudogenes were significant after multiple testing correction at (BH-FDR < 0.05). Of those, 5 pseudogenes were selected by a multivariate LASSO-Cox model, CTSLP8, EEF1GP4, HLA-K, CBX1P3, and RPS10P20. We find that increased CTSLP8 (Wald p-value = 5.0 × 10^− 05^), increased EEF1GP4 (Wald p-value = 1.0 × 10^− 06^), decreased HLA-K (Wald p-value = 8.0 × 10^− 05^), increased CBX1P3 (Wald p-value = 1.0 × 10^− 03^), and increased RPS10P20 (Wald p-value = 2.0 × 10^− 03^) indicate worse prognosis in breast cancer (Fig. 3a-c). Furthermore we find that the patients can be stratified into 4 distinct groups using a decision tree (all 14 BH-FDR significant pseudogenes were used as input) on only HLA-K, RPS10P20, and CTSLP8 (Fig. 3d). When clinical variables are added to the model, the patients can be further stratified into 6 groups (Fig. 3e). Individually, HLA-K has the most prognostic utility in breast cancer since median separation splits the patients (50.1 to 49.9%). Using the 323 unadjusted significant pseudogenes we are able to combine with the 1,007 unadjusted significant genes to produce a combined model of BRCA prognosis.

### Prognostic ability of pseudogene-gene interactions from PseudoFuN

The interactions between pseudogenes and genes are known to have an influence in multiple types of cancer [16–18, 35]. We use the unadjusted gene set and unadjusted pseudogene set as a starting point to determine possible pseudogene-gene interactions. From the PseudoFuN database, we found 77 candidate interactions that were added to the model. From the genes, pseudogenes, and interactions features we found that 40 total features had non-zero coefficients in our LASSO-Cox model and one corresponded to pseudogene-gene interactions. We fit the following model to our data using Cox regression on the retained gene, pseudogene, interaction, and interaction main effects resulting in 42 total features.

$$ h\left(t|{X}_{i,l}\right)={h}_0\left(\mathrm{t}\right)\exp \left(1.43\ast SLITRK3+14.21\ast GALP+2.79\ast OR4C13+3.23\ast VN1R4+4.84\ast L\mathrm{C}E3C+-0.11\ast STXBP5+0.01\ast IYD+0.51\ast ARID1B+0.78\ast SMR3A+-0.08\ast DIP2B+1.34\ast LOC100101266+-0.12\ast C19 orf66+-1.02\ast PARP12+0.21\ast EXOC1+-0.54\ast CEL+0.63\ast SLC9A1+-0.09\ast CCDC28B+0.59\ast PCMT1+1.88\ast C10 orf131+0.78\ast HARS2+1.69\ast CEL P+0.82\ast C7 orf53+1.33\ast OR52E6+0.18\ast HBS1L+11.26\ast MEMO1P3+0.34\ast HLA\_K+1.33\ast GVINP2+4.78\ast RPS10P20+5.93\ast HSPA8P1+0.47\ast GAPDHP45+3.04\ast ANKRD30 BP2+10.48\ast RPS20P25+10.79\ast KRT18P62+16.82\ast OR7E10P+0.26\ast SCML2P1+7.42\ast RPL5P28+2.56\ast RPS27P12+0.52\ast PRR13P5+-2.21\ast IGKV2D\_23+-0.11\ast GPS2+-6.96\ast GPS2P1+1.20\ast GPS2: GPS2P1\right) $$

**Fig. 4:**
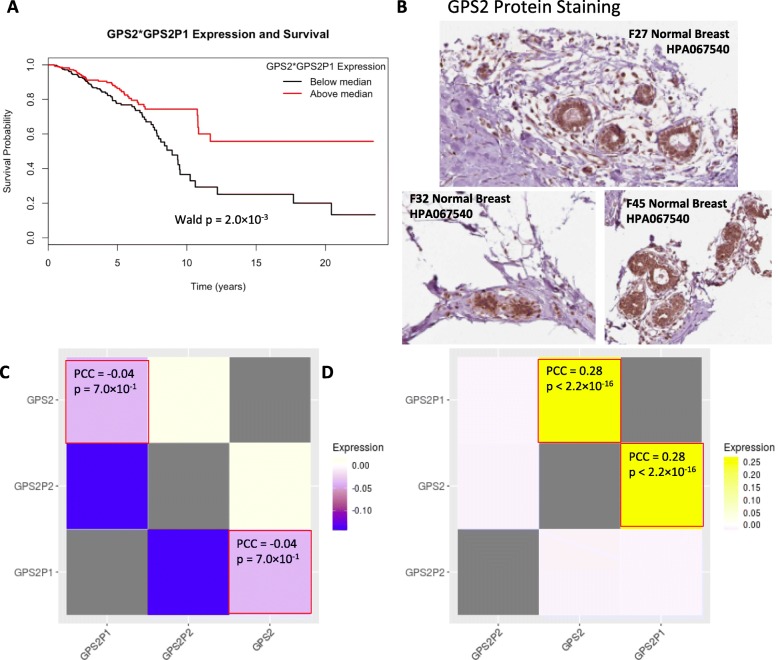
Pseudogene-gene interactions as survival prognosis features in BRCA. **a** Kaplan-Meier survival plot for GPS2-GPS2P1 interaction. **b** Histology staining from the Human Protein Atlas for GPS2 in normal tissue (includes: gender, age, tissue type, and antibody used in bold on the histology slide). The tissue staining is used to show that some protein product is present in normal tissue which could be affected by pseudogene regulation. **c** GPS2-GPS2P1 coexpression in normal and **d** tumor tissue in BRCA samples

Increased GPS2-GPS2P1 (Wald *p*-value = 2.0 × 10^− 03^) expression interaction indicates better prognosis (Fig. 4a). These observations are of interest considering neither the gene nor pseudogene alone are significant predictors of survival prognosis representing a cross-over (i.e., disordinal) interaction. Furthermore, we found that GPS2 protein is detectable at medium levels in both glandular cells and myoepithelial cells from normal breast tissue in the Human Protein Atlas [36] (Fig. 4c). GPS2 and GPS2P1 are uncorrelated in normal tissue but are correlated in primary tumor samples [21] (Fig. 4d, e) achieving a significantly higher correlation in tumor tissue (Fisher’s r to z transformation p-value < 0.0001). This relationship can indicate a regulatory change in breast cancer tissue. The TCGA miRNA data also shows that miR-3923 is significantly negatively correlated with GPS2 and predicted to target GPS2 by miRanda [37], PicTar [38], and TargetScan [39]. Upon further investigation, GPS2-GPS2P1 have high sequence homology in the top 1% of all comparisons in the PseudoFuN database. These observations are indicative of a ceRNA network. Considering that GPS2 siRNA knock-down causes increased proliferation of MCF-7 BRCA cell line [40], the GPS2-GPS2P1 interaction is an exciting prospect in BRCA regulation.

**Fig. 5:**
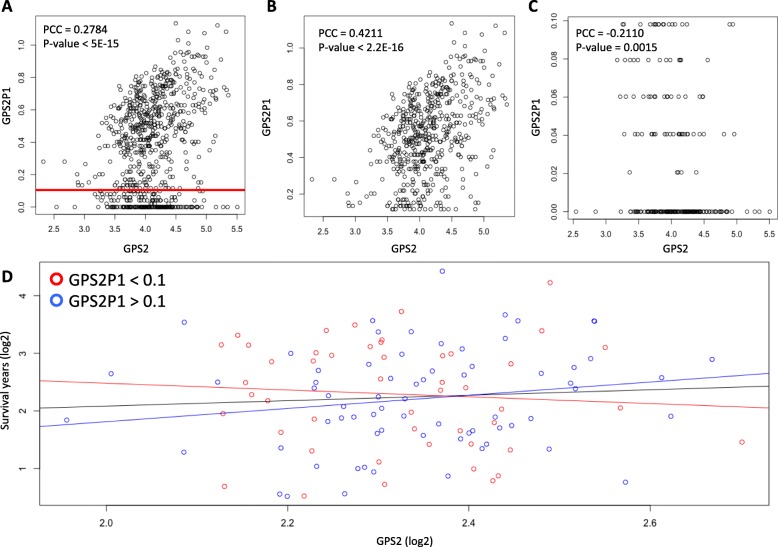
GPS2-GPS2P1 interaction terms and relationship with survival. All plots with survival included use the subset of patients with an event. **a-c** GPS2 and GPS2P1 associations including **b** GPS2 correlation with GPS2P1 for GPS2P1 high group (> 0.1, blue line in D, above red line in **a** and **c** GPS2 correlation with GPS2P1 for GPS2P1 low group (≤0.1, red line in D, below red line in **a**. **d** GPS2 association with survival for GPS2P1 high and low groups. Line color indicates the fit to the corresponding colored points and black color indicates all points. Note that only samples with events (i.e., survival times) could be used in **d**

Since the interpretation of cross-over interactions is more complicated we further evaluated the relationships between the independent and dependent variables in the cross-over interaction, GPS2-GPS2P1. GPS2P1 was positively correlated with GPS2 at high levels of GPS2P1 (Fig. 5b) and negatively correlated with GPS2 at low levels of GPS2P1 (Fig. 5c). These correlations were significantly different (Fisher’s r to z transformation *p*-value < 0.0001). We found for both interactions that pseudogene high expression and pseudogene low expression groups had a different association between the gene and survival using the subset of patients with events. Only the patients with events could be used so that the survival time in months was a valid dependent variable. The starkest relationship is that of GPS2 and GPS2P1 where GPS2 in the high GPS2P1 group was positively correlated with survival time while GPS2 in the low GPS2P1 group was negatively associated with survival time (Fig. 5d). These results could imply even more complicated interactions between genes and pseudogenes than previously understood. Ideally, more samples are needed to study the effect of pseudogene expression on gene-survival associations considering there were only 114 events (i.e., uncensored patient with time to survival) which drastically reduced our sample size in the gene-survival time comparisons.

### External validation of genes identified by interpretable models

In total 24 of the 42 Cox model features (see *Prognostic Ability of Pseudogene-Gene Interactions from PseudoFuN*) were also found in the Swedish cohort and the majority (16/18) of the missing features were pseudogenes. In fact, ANKRD30BP2 was the only pseudogene found in the Swedish cohort expression data. This is not surprising considering pseudogene expression is understudied, i.e., most datasets do not have quantified pseudogene expression and dreamBase has only calculated the pseudogene expression in TCGA data. Also, the pseudogene reads are most likely being assigned to a close homology sister genes. Pseudogene ANKRD30BP2 was found significantly associated with survival in the non-senior PAM50-normal patients (Wald *p*-value = 3.73 × 10^− 2^) and non-senior ER^+^HER2^+^ patients (Wald *p*-value = 3.73 × 10^− 2^) from the Swedish cohort. Of the 25 genes in the Cox model, 23 were found in the Swedish cohort and 16 of those genes were significant (Wald p-value < 5.00 × 10^− 2^) in at least one of the Swedish groups of patients. GPS2 was found to be associated with survival in non-senior patients (Wald *p*-value = 3.58 × 10^− 2^). These analyses also allow us to identify features that help stratify difficult to treat breast cancer subtypes, i.e., GALP, IYD, and PARP12 were all significantly associated with survival in non-senior triple-negative breast cancer patients. Additionally, all of the Wald *p*-values for each model feature in the Swedish cohort groups are contained in Additional file 2: Table S1.

## Discussion

### Insights

Many factors play a role on an individual’s prognosis, as demonstrated by the range of outcomes from our generated prognostic decision trees. One cannot assume an accurate prognosis simply based on molecular expression or clinical information - they should be considered as a unit. Not only do multiple factors need to be included for but likely molecular interactions must also be accounted for. Through LASSO-Cox regression and *ctree* visualization, it became apparent that high STXBP5 expression is a reliable indicator of a poor prognosis. STXBP5 encodes syntaxin binding protein 5 which can be readily detected in breast tissue (Fig. 2f). Perhaps what is most interesting is the mechanism that STXBP5 affects cancerous tissue. STXBP5-AS1 is a long non-coding RNA from the STXBP5 gene which is prognostic for breast cancer survival and may be regulated by miR-190 through ceRNA network interactions [41, 42]. Our results further support these previous findings and the idea of non-coding regulation of gene expression in cancer. It is important to not only view expression biomarkers from a gene level, and pseudogenes should also be considered.

Decreased HLA-K pseudogene expression was prognostic of poor patient survival. This finding is supported in the literature where multiple human leukocyte antigen (HLA) genes have been implicated in immune response to breast cancer [43]. Since the HLA genes encode major histocompatibility complex class I (MHC1) proteins, it is perhaps not surprising that a related pseudogene is prognostic. HLA pseudogenes have long been known to exist [44, 45] but the regulation of HLA genes by these pseudogenes has been understudied. Considering HLA genes are directly related to patient response to immunotherapy in lung cancer [46] and disease free survival in breast cancer [47], HLA-K should be further investigated.

Aside from identifying multiple individual genes and pseudogenes that are prognostic of patient survival, we leveraged prior knowledge of possible pseudogene-gene interactions from PseudoFuN [21] to identify prognostic interactions between genes and pseudogenes. In the case of GPS2-GPS2P1 there is clear evidence that the protein is expressed in normal breast tissue, that GPS2 and GPS2P1 are positively correlated in tumor samples (i.e., positive correlation is indicative of ceRNA interaction), there is evidence of miRNA targeting GPS2, and there are external experimental findings showing GPS2 miRNA regulation causes breast cancer cell proliferation by reducing GPS2 protein levels. Furthermore, we see interesting cross-over interactions in GPS2-GPS2P1 where the relationship between GPS2 and GPS2P1 changes as GPS2P1 expression increases. We also see changes in the relationship between GPS2 and survival time based on the expression of GPS2P1. Additionally this general wrokflow can be used in multiple cancer types since the pseudogene expression data is available for most TCGA cancer types.

With these considerations in mind, it should be possible to find additional genes, pseudogenes, and interactions in other cancers. Pseudogenes and pseudogene-gene interactions that are consistent across multiple cancers may be of interest as gene regulatory cancer drivers. Our integrative methodology, combining genes, pseudogenes, miRNAs, clinical variables, and a priori interaction information can improve the feature sets we use to model the complex systems in cancer.

### Limitations

Due to the dataset availability, we did not have an extremely large patient pool to work from. As more samples become available, our models should continue to be strengthened. With a larger sample size, we should find significance with miRNA data, enhancing our molecular model. Additionally, a larger sample size would increase the variety in patient clinical factors as well as the number of adverse events. Furthermore, pseudogene functional analysis and expression analysis is understudied resulting in a lack of these valuable data points for most available datasets. As a result, it is of the upmost importance to perform analyses like these shown so that the pseudogene knowledgebase can be enlarged.

## Conclusion

Given the highly unique characteristics cancer takes on in each impacted individual, the possibility of an effective universal cure is unlikely. Treatments have shifted to a more targeted approach to the molecular factors of specific subtypes. Furthermore, we are discovering novel regulatory relationships between different types of RNAs that can be leveraged in predictive models. Through statistically identifying key genes, pseudogenes, interactions and clinical factors impacting breast cancer survival to a more precise degree, treatments can become more individualized. Our study focuses on the use of a priori pseudogene-gene functional interactions to guide the features we use in predictive models. It is our conviction that by overlaying these regulatory relationships we can use the resulting interaction terms to improve the predictive accuracy of most any individual model type. We plan to use more regulatory data types (protein, expression and histology images) in further analysis to identify key interactions as features and to test alternative models such as random survival forests [48] and Cox-nnet neural networks [49].

## Supplementary information

**Additional file 1.** This file contains the R markdown output for the analysis. The plots and code in this file were directly used to generate the statistics and figures in the manuscript.

**Additional file 2.** This file contains the p-values for univariate cox proportional hazard models on each of the genes from our model in the Swedish cohort. The test was performed on multiple subsets of patients including age and breast cancer subtype using median expression as the cutoff.

## Data Availability

The major data, analyses and results are shared on GitHub: https://github.com/yanzhanglab/ICIBM_2019_SS_TSJ
