# Supplementary material for: Pseudogene-gene functional networks are prognostic of patient survival in breast cancer
Source: BMC Med Genomics. 2020 Apr 3;13(Suppl 5):51. doi: 10.1186/s12920-020-0687-0 (PMC7118805; doi:10.1186/s12920-020-0687-0)
Supplement: Supplementary file 1 — Additional file 1. This file contains the R markdown output for the analysis. The plots and code in this file were directly used to generate the statistics and figures in the manuscript. [file 12920_2020_687_MOESM1_ESM.pdf]

Pseudogene-gene functional networks are prognostic of patient survival in breast cancer.

Supplementary materials - R code and output.

Sasha Smerekanych, Travis Johnson, Kun Huang, Yan Zhang

Input files: Normalized RNA and clinical Tier 1 data from Firehose BRCA data.
Result description: Through opening both files, analysis can take place, understanding what genes/pseudogenes cause differential survival outcomes.

Setup and load libraries

```
## Install packages
#source("https://bioconductor.org/biocLite.R")
#biocLite(c("affy", "limma", "hgu95av2cdf", "hgu95av2.db", "genefilter", "multtest"))
#install.packages(c("glmnet", "Hmisc"))
library(rmarkdown)
library(affy)
## Loading required package: BiocGenerics
## Loading required package: parallel
##
## Attaching package: 'BiocGenerics'
## The following objects are masked from 'package:parallel':
##
##     clusterApply, clusterApplyLB, clusterCall, clusterEvalQ,
##     clusterExport, clusterMap, parApply, parCapply, parLapply,
##     parLapplyLB, parRapply, parSapply, parSapplyLB
## The following objects are masked from 'package:stats':
##
##     IQR, mad, sd, var, xtabs
## The following objects are masked from 'package:base':
##
##     anyDuplicated, append, as.data.frame, basename, cbind,
##     colMeans, colnames, colSums, dirname, do.call, duplicated,
##     eval, evalq, Filter, Find, get, grep, grepl, intersect,
##     is.unsorted, lapply, lengths, Map, mapply, match, mget, order,
##     paste, pmax, pmax.int, pmin, pmin.int, Position, rank, rbind,
##     Reduce, rowMeans, rownames, rowSums, sapply, setdiff, sort,
##     table, tapply, union, unique, unsplit, which, which.max,
##     which.min
## Loading required package: Biobase
## Welcome to Bioconductor
##
##     Vignettes contain introductory material; view with
##     'browseVignettes()'. To cite Bioconductor, see
##     'citation("Biobase")', and for packages 'citation("pkgname")'.
library(limma)
##
## Attaching package: 'limma'
## The following object is masked from 'package:BiocGenerics':
##
##     plotMA
library(hgu95av2cdf)
##
library(genefilter)
library(multtest)
library(hgu95av2.db)
## Loading required package: AnnotationDbi
## Loading required package: stats4
## Loading required package: IRanges
## Loading required package: S4Vectors
##
## Attaching package: 'S4Vectors'
## The following object is masked from 'package:base':
```

```
##
##     expand.grid
## Loading required package: org.Hs.eg.db
##
##
library(glmnet)
## Loading required package: Matrix
##
## Attaching package: 'Matrix'
## The following object is masked from 'package:S4Vectors':
##
##     expand
## Loading required package: foreach
## Loaded glmnet 2.0-16
library(Hmisc)
## Loading required package: lattice
## Loading required package: survival
## Loading required package: Formula
## Loading required package: ggplot2
##
## Attaching package: 'Hmisc'
## The following object is masked from 'package:AnnotationDbi':
##
##     contents
## The following object is masked from 'package:Biobase':
##
##     contents
## The following objects are masked from 'package:base':
##
##     format.pval, units
```

Read RNA-seq data

```
## Move to the correct working directory, then read in data.
header1 <-
unlist(read.table("~/Desktop/ICIBM_2019/gdac.broadinstitute.org_BRCA.Merge_rnaseq__ill
uminahiseq_rnaseq__unc_edu__Level_3__gene_expression__data.Level_3.2016012800.0.0/BRCA
.rnaseq__illuminahiseq_rnaseq__unc_edu__Level_3__gene_expression__data.data.txt",
                             nrows = 1, header = FALSE, stringsAsFactors = FALSE, sep
= "\t"))
header2 <-
unlist(read.table("~/Desktop/ICIBM_2019/gdac.broadinstitute.org_BRCA.Merge_rnaseq__ill
uminahiseq_rnaseq__unc_edu__Level_3__gene_expression__data.Level_3.2016012800.0.0/BRCA
.rnaseq__illuminahiseq_rnaseq__unc_edu__Level_3__gene_expression__data.data.txt",
                             skip = 1, nrows = 1, header = FALSE, stringsAsFactors =
FALSE, sep = "\t"))
names(header1) <- NULL
names(header2) <- NULL
dat <-
read.table("~/Desktop/ICIBM_2019/gdac.broadinstitute.org_BRCA.Merge_rnaseq__illuminahi
seq_rnaseq__unc_edu__Level_3__gene_expression__data.Level_3.2016012800.0.0/BRCA.rnaseq
__illuminahiseq_rnaseq__unc_edu__Level_3__gene_expression__data.data.txt",
                   skip = 2, header = FALSE, stringsAsFactors = FALSE, sep = "\t")

## Check data structure, they should be compatible.
str(header1)
##  chr [1:2635] "Hybridization REF" "TCGA-A1-A0SB-01A-11R-A144-07" ...
str(header2)
##  chr [1:2635] "gene" "raw_counts" "median_length_normalized" "RPKM" ...
header1[1:10]
##  [1] "Hybridization REF"            "TCGA-A1-A0SB-01A-11R-A144-07"
##  [3] "TCGA-A1-A0SB-01A-11R-A144-07" "TCGA-A1-A0SB-01A-11R-A144-07"
##  [5] "TCGA-A1-A0SD-01A-11R-A115-07" "TCGA-A1-A0SD-01A-11R-A115-07"
##  [7] "TCGA-A1-A0SD-01A-11R-A115-07" "TCGA-A1-A0SE-01A-11R-A084-07"
##  [9] "TCGA-A1-A0SE-01A-11R-A084-07" "TCGA-A1-A0SE-01A-11R-A084-07"
header2[1:10]
```

```
##  [1] "gene"                      "raw_counts"
##  [3] "median_length_normalized" "RPKM"
##  [5] "raw_counts"               "median_length_normalized"
##  [7] "RPKM"                     "raw_counts"
##  [9] "median_length_normalized" "RPKM"
dat[1:5, 1:10]
##              V1   V2        V3         V4   V5         V6         V7   V8
## 1 ?|100130426    0  0.000000  0.0000000    0   0.000000  0.0000000    0
## 2 ?|100133144  115  4.740894  1.1914669  134   5.538907  1.0832702  119
## 3 ?|100134869   59  1.850690  0.4632493   73   2.289837  0.4472326   72
## 4     ?|10357  269 21.147799  5.2935405  175  13.757862  2.6870755  392
## 5     ?|10431 1921 85.855098 21.5048610 3676 164.300537 32.1094332 5835
##           V9       V10
## 1   0.000000  0.0000000
## 2   4.852649  0.6488048
## 3   2.258469  0.2974940
## 4  30.817610  4.0594104
## 5 260.711091 34.3742365
## Gene expression data (mRNA seq)
#summary(dat[, (header2 == "RPKM")]) # Check value ranges
RNA.counts <- log2(dat[, (header2 == "RPKM")] + 1) # add 1e-10 then take log2 - YZ
20180707
RNA.counts[1:5, 1:10] # check data structure
##          V4        V7       V10       V13       V16       V19       V22
## 1 0.0000000 0.0000000 0.0000000 0.0000000 0.0000000 0.0000000 0.0000000
## 2 1.1318969 1.0588500 0.7214206 0.7886328 0.7519753 0.3930563 0.5928842
## 3 0.5491756 0.5332968 0.3757278 0.4971315 0.4363095 0.2797574 0.2663084
## 4 2.6538719 1.8824770 2.3389693 2.4956808 2.1464213 2.0462026 2.2302418
## 5 4.4921648 5.0491704 5.1446271 5.2273109 5.1346422 4.7456708 4.8835487
##         V25      V28       V31
## 1 0.0000000 0.000000 0.0000000
## 2 0.5849642 1.769816 0.6154439
## 3 0.2341860 1.106024 0.5405330
## 4 2.4951875 5.974958 2.1521699
## 5 4.5554912 5.320671 4.8346964
colnames(RNA.counts) <- substring(header1[header2 == "RPKM"], first = 1, last = 12)
rownames(RNA.counts) <- dat$V1
RNA.counts[29:35, 1:10] # check data structure
##              TCGA-A1-A0SB TCGA-A1-A0SD TCGA-A1-A0SE TCGA-A1-A0SF
## ?|90288        0.17241659  0.429408210  1.618660859  2.223554115
## A1BG|1         0.85294836  1.629600241  2.159952258  2.407789594
## A1CF|29974     0.00000000  0.000000000  0.000000000  0.008737625
## A2BP1|54715    0.09571601  0.003505648  0.004726608  0.000000000
## A2LD1|87769    1.17092382  1.825870116  1.841012912  2.685294292
## A2ML1|144568   0.40500662  0.472379329  0.271233111  0.354123929
## A2M|2          8.15720113  7.870114674  7.222408949  7.208264487
##              TCGA-A1-A0SG TCGA-A1-A0SH TCGA-A1-A0SI TCGA-A1-A0SJ
## ?|90288        1.64170715  0.422510460   1.56153970  0.142239409
## A1BG|1         1.76913811  1.904133650   1.53586028  2.308305276
## A1CF|29974     0.02381167  0.004138276   0.01432423  0.000000000
## A2BP1|54715    0.10379038  0.008888719   0.01922857  0.002522072
## A2LD1|87769    2.39317656  2.348299874   1.44453325  2.484279485
## A2ML1|144568   0.32279451  0.283304266   0.44259938  0.298939531
## A2M|2          6.94433090  6.832763431   6.56704504  7.658271385
##              TCGA-A1-A0SK TCGA-A1-A0SM
## ?|90288       0.226487805  0.619222935
## A1BG|1        1.357822329  2.571209423
## A1CF|29974    0.004241514  0.015532870
## A2BP1|54715   0.002282922  0.008375404
## A2LD1|87769   3.977806556  2.128351122
## A2ML1|144568  0.716054317  0.207288608
## A2M|2         5.342531235  6.308687380
dim(RNA.counts) # 20532   878
## [1] 20532   878
## Retain the tumor samples only - YZ 20180723
```

```
type <- substring(header1[header2 == "RPKM"], first = 14, last = 15)
table(type) # 01: Primary Solid Tumor, 06: Metastatic, 11: Solid Tissue Normal
## type
##  01  06  11
## 775   3 100
RNA.counts.01 <- RNA.counts[, type == "01"]
dim(RNA.counts.01) # 20532   775
## [1] 20532   775
RNA.counts.01[29:35, 1:10] # check data structure
##              TCGA-A1-A0SB TCGA-A1-A0SD TCGA-A1-A0SE TCGA-A1-A0SF
## ?|90288        0.17241659  0.429408210  1.618660859  2.223554115
## A1BG|1         0.85294836  1.629600241  2.159952258  2.407789594
## A1CF|29974     0.00000000  0.000000000  0.000000000  0.008737625
## A2BP1|54715    0.09571601  0.003505648  0.004726608  0.000000000
## A2LD1|87769    1.17092382  1.825870116  1.841012912  2.685294292
## A2ML1|144568   0.40500662  0.472379329  0.271233111  0.354123929
## A2M|2          8.15720113  7.870114674  7.222408949  7.208264487
##              TCGA-A1-A0SG TCGA-A1-A0SH TCGA-A1-A0SI TCGA-A1-A0SJ
## ?|90288        1.64170715  0.422510460   1.56153970  0.142239409
## A1BG|1         1.76913811  1.904133650   1.53586028  2.308305276
## A1CF|29974     0.02381167  0.004138276   0.01432423  0.000000000
## A2BP1|54715    0.10379038  0.008888719   0.01922857  0.002522072
## A2LD1|87769    2.39317656  2.348299874   1.44453325  2.484279485
## A2ML1|144568   0.32279451  0.283304266   0.44259938  0.298939531
## A2M|2          6.94433090  6.832763431   6.56704504  7.658271385
##              TCGA-A1-A0SK TCGA-A1-A0SM
## ?|90288       0.226487805  0.619222935
## A1BG|1        1.357822329  2.571209423
## A1CF|29974    0.004241514  0.015532870
## A2BP1|54715   0.002282922  0.008375404
## A2LD1|87769   3.977806556  2.128351122
## A2ML1|144568  0.716054317  0.207288608
## A2M|2         5.342531235  6.308687380
sum(table(colnames(RNA.counts.01)) > 1) # this value should be 0, meaning no redundant
cancer samples.
## [1] 0
```

Read survival data

```
data_clinical_patient <-
read.table("~/Desktop/ICIBM_2019/gdac.broadinstitute.org_BRCA.Clinical_Pick_Tier1.Leve
l_4.2016012800.0.0/BRCA.clin.merged.picked.txt",
                                    header=TRUE, stringsAsFactors = FALSE, sep="\t")
colnames(data_clinical_patient) <- gsub("\\.", "-",
toupper(colnames(data_clinical_patient)))
rownames(data_clinical_patient) <- data_clinical_patient[, 1]
data_clinical_patient <- data_clinical_patient[, -1] # Run this only once
str(data_clinical_patient) # Still chr columns
## 'data.frame':    18 obs. of  1097 variables:
##  $ TCGA-5L-AAT0: chr  "value" "42" "0" NA ...
##  $ TCGA-5L-AAT1: chr  "value" "63" "0" NA ...
##  $ TCGA-A1-A0SP: chr  "value" "40" "0" NA ...
##  $ TCGA-A2-A04V: chr  "value" "39" "1" "1920" ...
##  $ TCGA-A2-A04Y: chr  "value" "53" "0" NA ...
##  $ TCGA-A2-A0CQ: chr  "value" "62" "0" NA ...
##  $ TCGA-A2-A1G4: chr  "value" "71" "0" NA ...
##  $ TCGA-A2-A25A: chr  "value" "44" "0" NA ...
##  $ TCGA-A7-A0CD: chr  "value" "66" "0" NA ...
##  $ TCGA-A7-A13G: chr  "value" "79" "0" NA ...
##  $ TCGA-A7-A26E: chr  "value" "71" "0" NA ...
##  $ TCGA-A7-A26F: chr  "value" "55" "0" NA ...
##  $ TCGA-A7-A26H: chr  "value" "72" "0" NA ...
##  $ TCGA-A7-A26I: chr  "value" "65" "0" NA ...
##  $ TCGA-A7-A2KD: chr  "value" "53" "0" NA ...
##  $ TCGA-A7-A3J1: chr  "value" "63" "0" NA ...
##  $ TCGA-A7-A426: chr  "value" "50" "0" NA ...
```

```
##  $ TCGA-A7-A5ZX: chr  "value" "48" "0" NA ...
##  $ TCGA-A8-A06T: chr  "value" "74" "0" NA ...
##  $ TCGA-A8-A06U: chr  "value" "80" "1" "883" ...
##  $ TCGA-A8-A07E: chr  "value" "81" "0" NA ...
##  $ TCGA-A8-A084: chr  "value" "81" "0" NA ...
##  $ TCGA-A8-A08F: chr  "value" "59" "0" NA ...
##  $ TCGA-A8-A08S: chr  "value" "71" "0" NA ...
##  $ TCGA-A8-A091: chr  "value" "61" "0" NA ...
##  $ TCGA-A8-A093: chr  "value" "61" "0" NA ...
##  $ TCGA-A8-A09C: chr  "value" "69" "0" NA ...
##  $ TCGA-A8-A09E: chr  "value" "73" "0" NA ...
##  $ TCGA-A8-A09I: chr  "value" "84" "0" NA ...
##  $ TCGA-A8-A09K: chr  "value" "68" "0" NA ...
##  $ TCGA-AC-A3W5: chr  "value" "65" "0" NA ...
##  $ TCGA-AC-A3W6: chr  "value" "90" "0" NA ...
##  $ TCGA-AC-A5EI: chr  "value" "88" "0" NA ...
##  $ TCGA-AC-A5XS: chr  "value" "74" "0" NA ...
##  $ TCGA-AC-A62X: chr  "value" "72" "0" NA ...
##  $ TCGA-AO-A0JI: chr  "value" "56" "0" NA ...
##  $ TCGA-AO-A1KT: chr  "value" "78" "0" NA ...
##  $ TCGA-AQ-A0Y5: chr  "value" "70" "1" "172" ...
##  $ TCGA-AQ-A54N: chr  "value" "51" "0" NA ...
##  $ TCGA-AQ-A54O: chr  "value" "51" "0" NA ...
##  $ TCGA-AQ-A7U7: chr  "value" "55" "1" "584" ...
##  $ TCGA-AR-A1AM: chr  "value" "52" "0" NA ...
##  $ TCGA-AR-A1AR: chr  "value" "50" "1" "524" ...
##  $ TCGA-AR-A2LL: chr  "value" "70" "0" NA ...
##  $ TCGA-AR-A2LR: chr  "value" "49" "0" NA ...
##  $ TCGA-AR-A5QM: chr  "value" "62" "0" NA ...
##  $ TCGA-B6-A0I8: chr  "value" "46" "1" "749" ...
##  $ TCGA-BH-A0B6: chr  "value" "47" "0" NA ...
##  $ TCGA-BH-A0DO: chr  "value" "78" "0" NA ...
##  $ TCGA-BH-A0EA: chr  "value" "72" "1" "991" ...
##  $ TCGA-BH-A18H: chr  "value" "63" "0" NA ...
##  $ TCGA-BH-A1F5: chr  "value" "62" "1" "2712" ...
##  $ TCGA-BH-A8FY: chr  "value" "87" "1" "295" ...
##  $ TCGA-C8-A1HL: chr  "value" NA "0" NA ...
##  $ TCGA-D8-A146: chr  "value" "57" "0" NA ...
##  $ TCGA-E2-A158: chr  "value" "43" "0" NA ...
##  $ TCGA-E2-A15O: chr  "value" "89" "0" NA ...
##  $ TCGA-E2-A1BC: chr  "value" "63" "0" NA ...
##  $ TCGA-E2-A1L8: chr  "value" "52" "0" NA ...
##  $ TCGA-E2-A1LG: chr  "value" "50" "0" NA ...
##  $ TCGA-E2-A2P5: chr  "value" "78" "1" "821" ...
##  $ TCGA-E2-A572: chr  "value" "72" "0" NA ...
##  $ TCGA-EW-A1J5: chr  "value" "59" "0" NA ...
##  $ TCGA-EW-A1P3: chr  "value" "48" "0" NA ...
##  $ TCGA-GM-A2DD: chr  "value" "53" "0" NA ...
##  $ TCGA-GM-A2DO: chr  "value" "54" "0" NA ...
##  $ TCGA-LD-A74U: chr  "value" "79" "0" NA ...
##  $ TCGA-LL-A440: chr  "value" "61" "0" NA ...
##  $ TCGA-LL-A442: chr  "value" "56" "0" NA ...
##  $ TCGA-LL-A50Y: chr  "value" "84" "0" NA ...
##  $ TCGA-LL-A5YP: chr  "value" "49" "0" NA ...
##  $ TCGA-LL-A740: chr  "value" "61" "0" NA ...
##  $ TCGA-OL-A5RU: chr  "value" "63" "0" NA ...
##  $ TCGA-S3-AA12: chr  "value" "82" "0" NA ...
##  $ TCGA-S3-AA17: chr  "value" "64" "0" NA ...
##  $ TCGA-W8-A86G: chr  "value" "66" "0" NA ...
##  $ TCGA-WT-AB41: chr  "value" NA "0" NA ...
##  $ TCGA-Z7-A8R5: chr  "value" "61" "0" NA ...
##  $ TCGA-3C-AAAU: chr  "value" "55" "0" NA ...
##  $ TCGA-3C-AALI: chr  "value" "50" "0" NA ...
##  $ TCGA-3C-AALJ: chr  "value" "62" "0" NA ...
##  $ TCGA-3C-AALK: chr  "value" "52" "0" NA ...
```

```
##  $ TCGA-4H-AAAK: chr  "value" "50" "0" NA ...
##  $ TCGA-5T-A9QA: chr  "value" "52" "0" NA ...
##  $ TCGA-A1-A0SB: chr  "value" "70" "0" NA ...
##  $ TCGA-A1-A0SD: chr  "value" "59" "0" NA ...
##  $ TCGA-A1-A0SE: chr  "value" "56" "0" NA ...
##  $ TCGA-A1-A0SF: chr  "value" "54" "0" NA ...
##  $ TCGA-A1-A0SG: chr  "value" "61" "0" NA ...
##  $ TCGA-A1-A0SH: chr  "value" "39" "0" NA ...
##  $ TCGA-A1-A0SI: chr  "value" "52" "0" NA ...
##  $ TCGA-A1-A0SJ: chr  "value" "39" "0" NA ...
##  $ TCGA-A1-A0SK: chr  "value" "54" "1" "967" ...
##  $ TCGA-A1-A0SM: chr  "value" "77" "0" NA ...
##  $ TCGA-A1-A0SN: chr  "value" "50" "0" NA ...
##  $ TCGA-A1-A0SO: chr  "value" "67" "0" NA ...
##  $ TCGA-A1-A0SQ: chr  "value" "45" "0" NA ...
##  $ TCGA-A2-A04N: chr  "value" "66" "0" NA ...
##  $ TCGA-A2-A04P: chr  "value" "36" "1" "548" ...
##   [list output truncated]
data_clinical_patient[, 1:6] # check data structure
##                                                         TCGA-5L-AAT0
## Composite Element REF                                          value
## years_to_birth                                                    42
## vital_status                                                       0
## days_to_death                                                   <NA>
## days_to_last_followup                                           1477
## tumor_tissue_site                                             breast
## pathologic_stage                                           stage iia
## pathology_T_stage                                                 t2
## pathology_N_stage                                                 n0
## pathology_M_stage                                                 m0
## gender                                                        female
## date_of_initial_pathologic_diagnosis                            2010
## days_to_last_known_alive                                        <NA>
## radiation_therapy                                                yes
## histological_type                     infiltrating lobular carcinoma
## number_of_lymph_nodes                                              0
## race                                                           white
## ethnicity                                         hispanic or latino
##                                                         TCGA-5L-AAT1
## Composite Element REF                                          value
## years_to_birth                                                    63
## vital_status                                                       0
## days_to_death                                                   <NA>
## days_to_last_followup                                           1471
## tumor_tissue_site                                             breast
## pathologic_stage                                            stage iv
## pathology_T_stage                                                 t2
## pathology_N_stage                                                 n0
## pathology_M_stage                                                 m1
## gender                                                        female
## date_of_initial_pathologic_diagnosis                            2010
## days_to_last_known_alive                                        <NA>
## radiation_therapy                                                 no
## histological_type                     infiltrating lobular carcinoma
## number_of_lymph_nodes                                              0
## race                                                           white
## ethnicity                                         hispanic or latino
##                                                        TCGA-A1-A0SP
## Composite Element REF                                         value
## years_to_birth                                                   40
## vital_status                                                      0
## days_to_death                                                  <NA>
## days_to_last_followup                                           584
## tumor_tissue_site                                            breast
## pathologic_stage                                          stage iia
```

```
## pathology_T_stage                                                 t2
## pathology_N_stage                                           n0 (i-)
## pathology_M_stage                                                 m0
## gender                                                        female
## date_of_initial_pathologic_diagnosis                            2007
## days_to_last_known_alive                                        <NA>
## radiation_therapy                                               <NA>
## histological_type                    infiltrating ductal carcinoma
## number_of_lymph_nodes                                              0
## race                                                            <NA>
## ethnicity                                   not hispanic or latino
##                                                       TCGA-A2-A04V
## Composite Element REF                                          value
## years_to_birth                                                    39
## vital_status                                                       1
## days_to_death                                                   1920
## days_to_last_followup                                           <NA>
## tumor_tissue_site                                             breast
## pathologic_stage                                           stage iia
## pathology_T_stage                                                 t2
## pathology_N_stage                                           n0 (i-)
## pathology_M_stage                                                 m0
## gender                                                        female
## date_of_initial_pathologic_diagnosis                            2005
## days_to_last_known_alive                                        <NA>
## radiation_therapy                                                 no
## histological_type                    infiltrating ductal carcinoma
## number_of_lymph_nodes                                              0
## race                                                           white
## ethnicity                                   not hispanic or latino
##                                                       TCGA-A2-A04Y
## Composite Element REF                                          value
## years_to_birth                                                    53
## vital_status                                                       0
## days_to_death                                                   <NA>
## days_to_last_followup                                           1099
## tumor_tissue_site                                             breast
## pathologic_stage                                           stage iib
## pathology_T_stage                                                 t2
## pathology_N_stage                                               n1mi
## pathology_M_stage                                                 m0
## gender                                                        female
## date_of_initial_pathologic_diagnosis                            2008
## days_to_last_known_alive                                        <NA>
## radiation_therapy                                                yes
## histological_type                    infiltrating ductal carcinoma
## number_of_lymph_nodes                                              1
## race                                                           white
## ethnicity                                                       <NA>
##                                                       TCGA-A2-A0CQ
## Composite Element REF                                          value
## years_to_birth                                                    62
## vital_status                                                       0
## days_to_death                                                   <NA>
## days_to_last_followup                                           2695
## tumor_tissue_site                                             breast
## pathologic_stage                                            stage ia
## pathology_T_stage                                                 t1
## pathology_N_stage                                           n0 (i-)
## pathology_M_stage                                                 m0
## gender                                                        female
## date_of_initial_pathologic_diagnosis                            2003
## days_to_last_known_alive                                        <NA>
## radiation_therapy                                                yes
## histological_type                    infiltrating ductal carcinoma
```

```
## number_of_lymph_nodes                                                    0
## race                                          black or african american
## ethnicity                                        not hispanic or latino
```

Survival Analysis

We want to look at overall patient survival, simply comparing the number of those who lived and those who died using a table, then histograms (separately).

```
data_clinical_patient <- t(data_clinical_patient)
data_clinical_patient <- data.frame(data_clinical_patient, stringsAsFactors = FALSE)
str(data_clinical_patient)
## 'data.frame':    1097 obs. of  18 variables:
##  $ Composite.Element.REF             : chr  "value" "value" "value" "value" ...
##  $ years_to_birth                    : chr  "42" "63" "40" "39" ...
##  $ vital_status                      : chr  "0" "0" "0" "1" ...
##  $ days_to_death                     : chr  NA NA NA "1920" ...
##  $ days_to_last_followup             : chr  "1477" "1471" "584" NA ...
##  $ tumor_tissue_site                 : chr  "breast" "breast" "breast"
"breast" ...
##  $ pathologic_stage                  : chr  "stage iia" "stage iv" "stage iia"
"stage iia" ...
##  $ pathology_T_stage                 : chr  "t2" "t2" "t2" "t2" ...
##  $ pathology_N_stage                 : chr  "n0" "n0" "n0 (i-)" "n0 (i-)" ...
##  $ pathology_M_stage                 : chr  "m0" "m1" "m0" "m0" ...
##  $ gender                            : chr  "female" "female" "female"
"female" ...
##  $ date_of_initial_pathologic_diagnosis: chr  "2010" "2010" "2007" "2005" ...
##  $ days_to_last_known_alive          : chr  NA NA NA NA ...
##  $ radiation_therapy                 : chr  "yes" "no" NA "no" ...
##  $ histological_type                 : chr  "infiltrating lobular carcinoma"
"infiltrating lobular carcinoma" "infiltrating ductal carcinoma" "infiltrating ductal
carcinoma" ...
##  $ number_of_lymph_nodes             : chr  "0" "0" "0" "0" ...
##  $ race                              : chr  "white" "white" NA "white" ...
##  $ ethnicity                         : chr  "hispanic or latino" "hispanic or
latino" "not hispanic or latino" "not hispanic or latino" ...
## Important: Change some variables into numeric.
data_clinical_patient$years_to_birth <-
as.numeric(data_clinical_patient$years_to_birth)
data_clinical_patient$vital_status <- as.numeric(data_clinical_patient$vital_status)
data_clinical_patient$days_to_death <- as.numeric(data_clinical_patient$days_to_death)
data_clinical_patient$days_to_last_followup <-
as.numeric(data_clinical_patient$days_to_last_followup)
data_clinical_patient$date_of_initial_pathologic_diagnosis <-
as.numeric(data_clinical_patient$date_of_initial_pathologic_diagnosis)
data_clinical_patient$number_of_lymph_nodes <-
as.numeric(data_clinical_patient$number_of_lymph_nodes)
data_clinical_patient$days_to_last_known_alive <-
as.numeric(data_clinical_patient$days_to_last_known_alive)
str(data_clinical_patient)
## 'data.frame':    1097 obs. of  18 variables:
##  $ Composite.Element.REF             : chr  "value" "value" "value" "value" ...
##  $ years_to_birth                    : num  42 63 40 39 53 62 71 44 66 79 ...
##  $ vital_status                      : num  0 0 0 1 0 0 0 0 0 0 ...
##  $ days_to_death                     : num  NA NA NA 1920 NA NA NA NA NA NA ...
##  $ days_to_last_followup             : num  1477 1471 584 NA 1099 ...
##  $ tumor_tissue_site                 : chr  "breast" "breast" "breast"
"breast" ...
##  $ pathologic_stage                  : chr  "stage iia" "stage iv" "stage iia"
"stage iia" ...
##  $ pathology_T_stage                 : chr  "t2" "t2" "t2" "t2" ...
##  $ pathology_N_stage                 : chr  "n0" "n0" "n0 (i-)" "n0 (i-)" ...
##  $ pathology_M_stage                 : chr  "m0" "m1" "m0" "m0" ...
##  $ gender                            : chr  "female" "female" "female"
"female" ...
##  $ date_of_initial_pathologic_diagnosis: num  2010 2010 2007 2005 2008 ...
```

```
##  $ days_to_last_known_alive            : num  NA NA NA NA NA NA NA NA NA NA ...
##  $ radiation_therapy                   : chr  "yes" "no" NA "no" ...
##  $ histological_type                   : chr  "infiltrating lobular carcinoma"
"infiltrating lobular carcinoma" "infiltrating ductal carcinoma" "infiltrating ductal
carcinoma" ...
##  $ number_of_lymph_nodes               : num  0 0 0 0 1 0 2 0 0 0 ...
##  $ race                                : chr  "white" "white" NA "white" ...
##  $ ethnicity                           : chr  "hispanic or latino" "hispanic or
latino" "not hispanic or latino" "not hispanic or latino" ...
table(data_clinical_patient$tumor_tissue_site)
##
## breast
##   1097
table(data_clinical_patient$vital_status)
##
##   0   1
## 945 152
```

Create days_to_death_or_last_fup following Broad conversion process

```
surdata <- data_clinical_patient
surdata$days_to_death_or_fup <- NA # initialization
```

1. if 'vital_status'==1(dead), 'days_to_last_followup' is always NA. Thus, uses 'days_to_death' value for 'days_to_death_or_fup'

```
idx <- (surdata$vital_status == 1)
surdata$days_to_death_or_fup[idx] <- surdata$days_to_death[idx]
```

2. if 'vital_status'==0(alive) 2.1. if 'days_to_death'==NA & 'days_to_last_followup'!=NA, uses 'days_to_last_followup' value for 'days_to_death_or_fup'

```
idx <- (surdata$vital_status == 0) & (is.na(surdata$days_to_death))
& !(is.na(surdata$days_to_last_followup))
surdata$days_to_death_or_fup[idx] <- surdata$days_to_last_followup[idx]
```

2.2. if 'days_to_death'!=NA, excludes this case in survival analysis and report the case.

```
idx <- (surdata$vital_status == 0) & !(is.na(surdata$days_to_death))
sum(idx) # this should be 0. Otherwise we should excludes this case in survival
analysis and report the case.
## [1] 0
```

3. if 'vital_status'==NA,excludes this case in survival analysis and report the case.

```
sum(is.na(surdata$vital_status)) # this should be 0. Otherwise excludes this case in
survival analysis and report the case.
## [1] 0
```

After the "Broad process": remove NAs, and remove patients if their days_to_death_or_fup == 0

```
sum(is.na(surdata$days_to_death_or_fup)) # 1
## [1] 1
sum(surdata$days_to_death_or_fup == 0) # NA
## [1] NA
idx <- !(is.na(surdata$days_to_death_or_fup)) & (surdata$days_to_death_or_fup >0)
surdata2 <- surdata[idx,]
dim(surdata2) # 1083  19
## [1] 1083   19
rm(idx)

surdata2$survival.years <- (surdata2$days_to_death_or_fup)/365
sum(is.na(surdata2$survival.years)) # 0
## [1] 0
```

Plot survival time

```
hist(surdata2$survival.years, main = "Overall survival", xlab = "Survival Time
(years)")
```

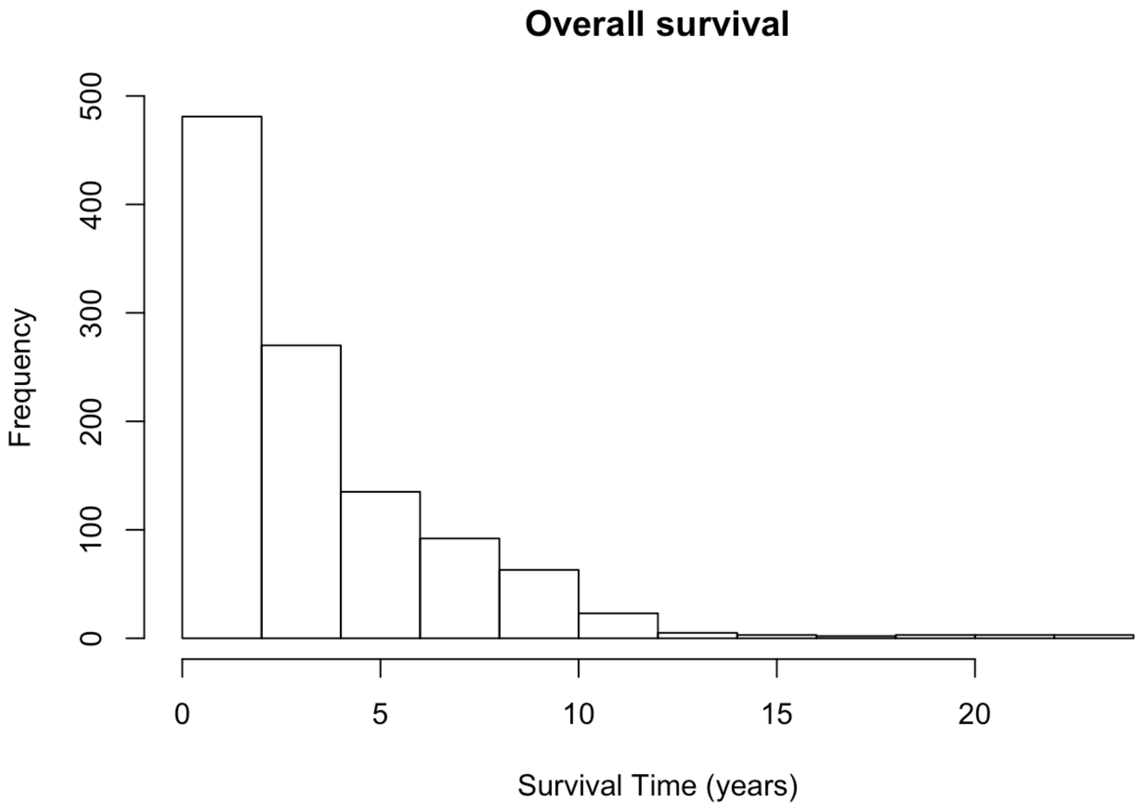


```
# Among those who died
hist(surdata2$survival.years[surdata2$vital_status == 1], main = "Overall survival
(deceased)",
     xlab = "Survival Time (years)")
```

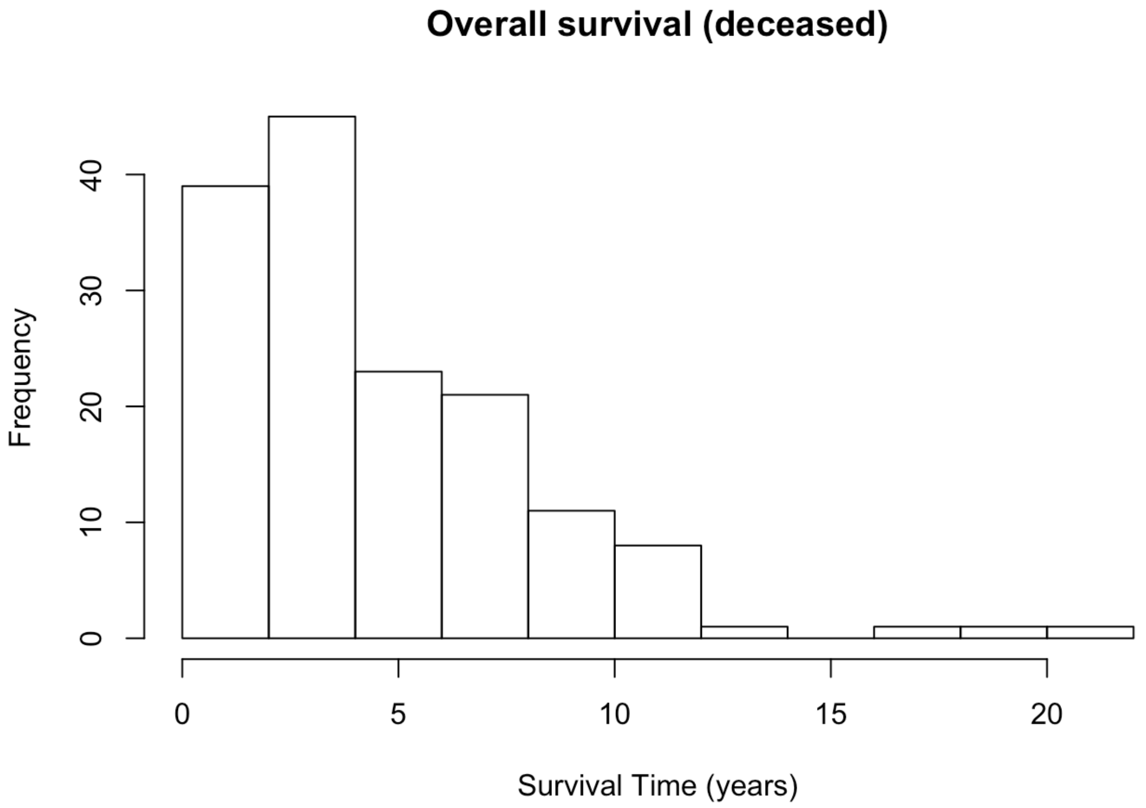


```
# Among those still alive
hist(surdata2$survival.years[surdata2$vital_status == 0], main = "Overall survival
(alive)",
     xlab = "Survival Time (years)")
```

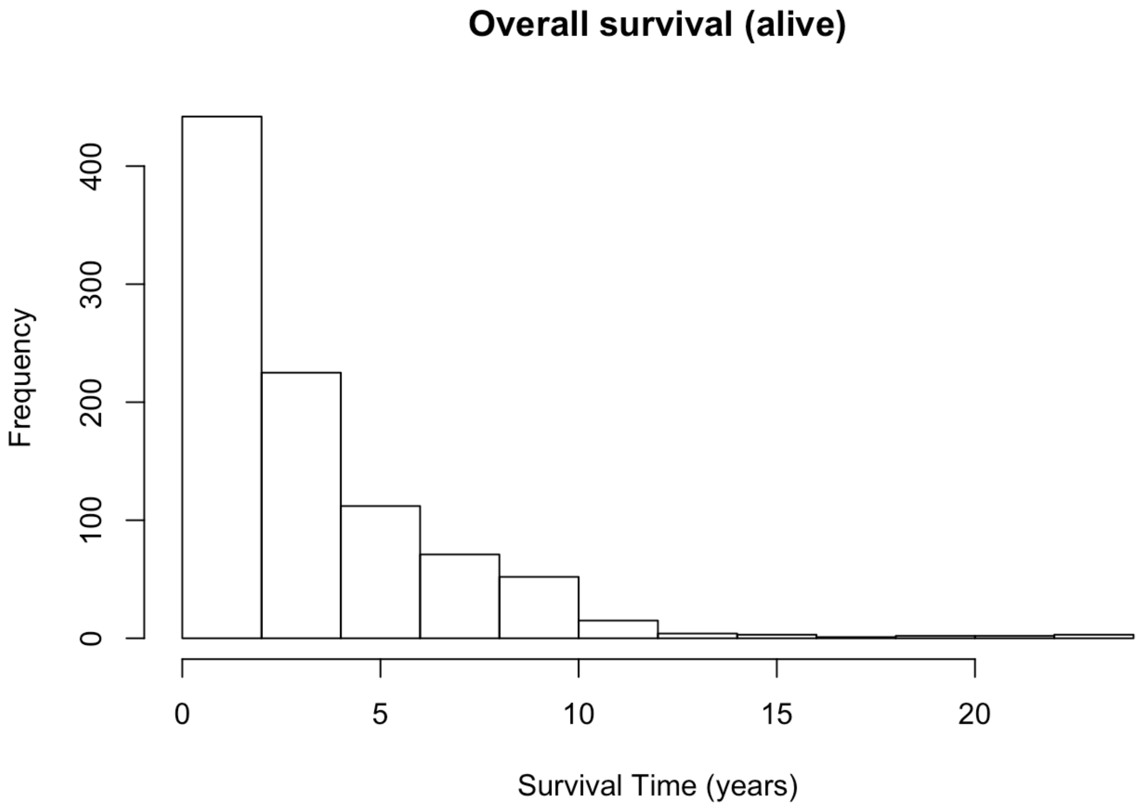

Visualizing survival plot - survival time vs. proportion still alive using a Kaplan Meier plot.

First, organize formats to align:

```
common.patients <- base::intersect(colnames(RNA.counts.01), rownames(surdata2))

## Check the number of patients after filtering
nrow(surdata) # 1097
## [1] 1097
nrow(surdata2) # 1083
## [1] 1083
length(common.patients) # 765
## [1] 765
surdata3 <- surdata2[common.patients,]
dim(surdata3) # 765  20 <------ Use surdata3
## [1] 765  20
##############################################################################
## Kaplan-Meier Plot for Overall Survival
##############################################################################
require(survival)
km <- survfit(Surv(survival.years, vital_status) ~ 1, data = surdata3)
plot(km, xlab = "Time (years)",  ylab = "Survival Probability",lwd = 2, main =
"Overall Survival")
```

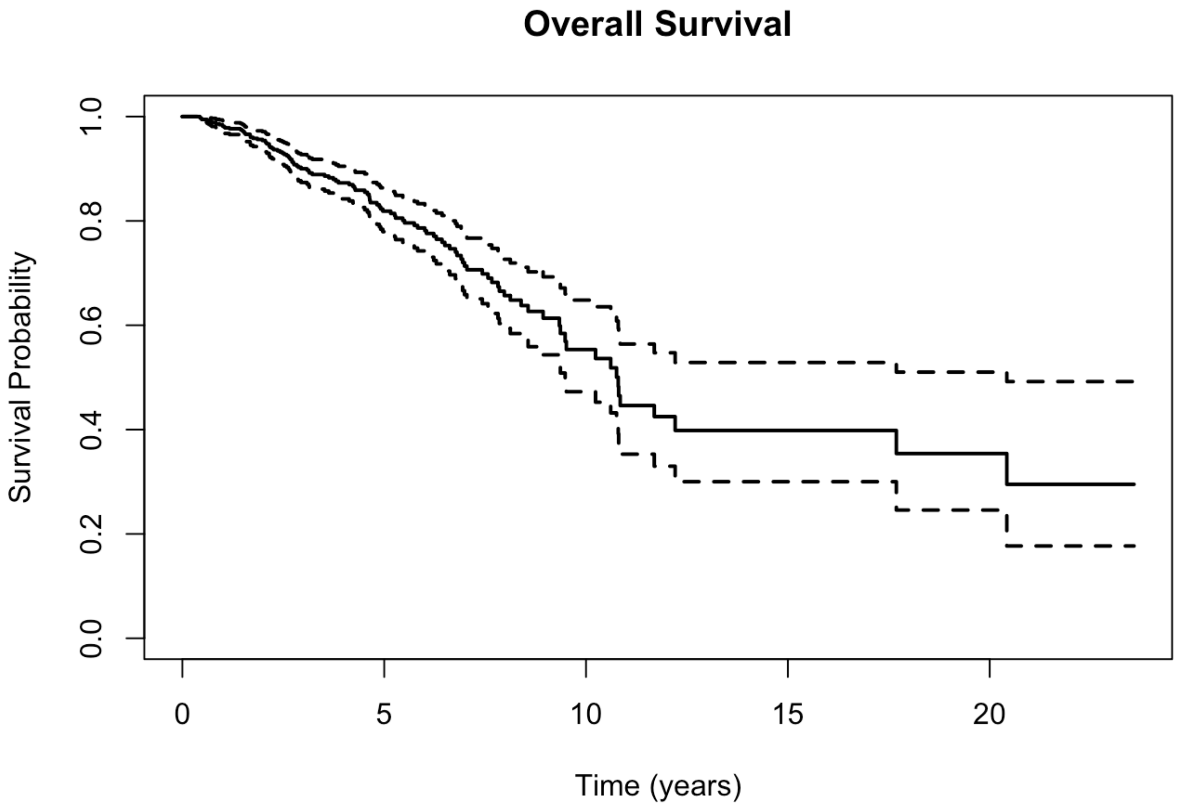


```
##We want to format the data frames (RNA.counts.01 and surdata) so we can compare gene
expression with survival data

#YZ's check point: Count the number of NAs per gene
na_count <- apply(RNA.counts.01, 1, function(x) sum(is.na(x)))
table(na_count) # It seems there is no NA.
## na_count
##     0
## 20532
#Transpose data
RNA.counts.01 <- t(RNA.counts.01) # Run this only once
RNA.counts.01 <- RNA.counts.01[common.patients,] # <------ Use this RNA.counts.01

## double-check that rownames are equal
all.equal(rownames(RNA.counts.01), rownames(surdata3)) # TRUE
## [1] TRUE
length(rownames(RNA.counts.01)) # 765
## [1] 765
```

Multivariable model In order to create a multivariable model to assess multiple genes at once, we need to split the data into a training set and a test set. The model is built on the training data, and assessed using the test data, which enables us to avoid over-fitting. We then have a molecular signature which will tell us the prognosis of a patient based on a number of genes' expression levels.

```
## Split the data into 2/3 training and 1/3 test
## Fit model on 2/3 and evaluate on the test data

## X = expression data (patients in rows and genes in columns)
## S = Surv(survival.time, survival.status)

## GENE EXPRESSION DATA = RNA.counts1

## SURVIVAL
```

```
#S <- Surv(as.numeric(surdata3$survival.years, surdata3$OS_STATUS))
S <- Surv(surdata3$survival.years, surdata3$vital_status)

### training data and validation data
set.seed(1000) # set the seed to ensure reproducibility

## Traing set rows
## randomly sample 2/3 of subjects
train.idx <- sample(1:nrow(surdata3), round(nrow(surdata3)*2/3), replace = FALSE)
length(train.idx) # 510
## [1] 510
nrow(surdata3) # 765
## [1] 765
X.train <- RNA.counts.01[train.idx,]
S.train <- S[train.idx,]

## For test set rows use '-train.idx'
X.test <- RNA.counts.01[-train.idx,]
S.test <- S[-train.idx,]

## Check dimensions
dim(X.train) # 510 20532
## [1]   510 20532
dim(X.test) # 255 20532
## [1]   255 20532
```
We want to select the features for our model, which is the expression level of genes that are most associated with survival - using the filtering method.
```
## fitting univariate models
cox.uni.p <- numeric(ncol(X.train))
m <- ncol(X.train)
for (i in 1:m)  {
  if (i != 4818){
      #print(i)
      cox0 <- coxph(S.train ~ X.train[,i])
      cox.uni.p[i] <- 1-pchisq(cox0$score, 1) # error when message i=4818, matrix
singularity error?
  }
}
names(cox.uni.p) <- colnames(X.train)
## Takes a minute but not bad
## YZ will double check Warning messages:
## 1: In fitter(X, Y, strats, offset, init, control, weights = weights,  ... :
##               Loglik converged before variable  1 ; beta may be infinite.

## histogram of univariate p-values
hist(cox.uni.p,main="",xlab="Univariate p-values")
```

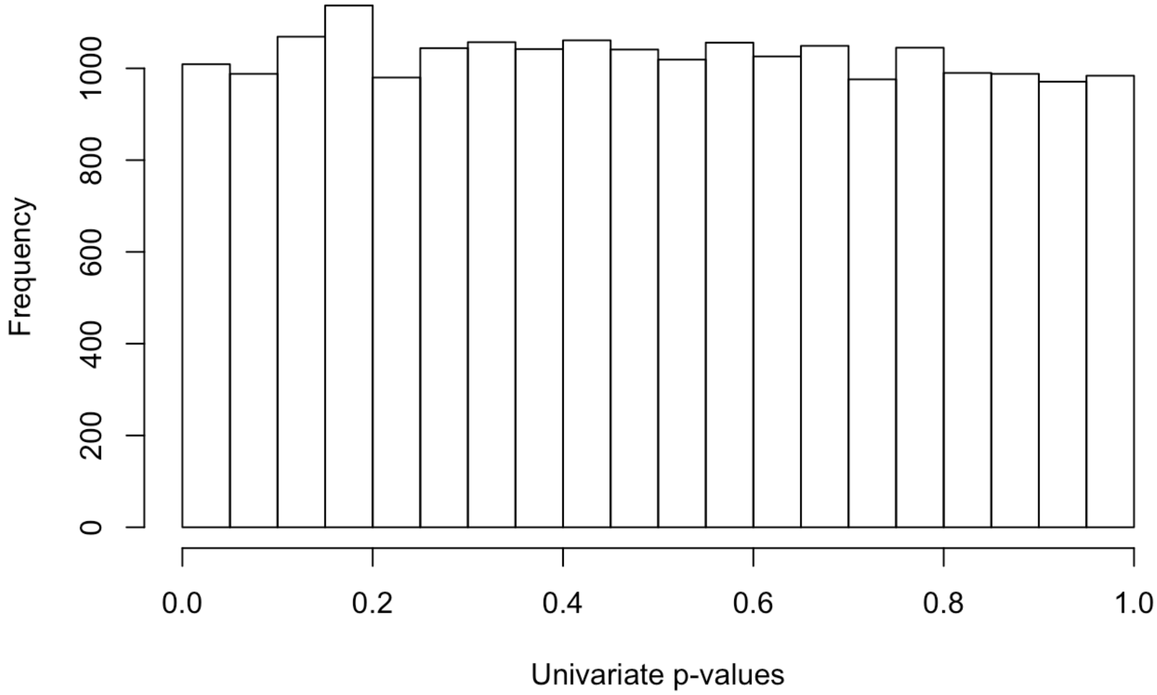


```
## Screen the top based on p-values, and required the adjusted p-value < 0.05
cox.uni.p <- sort(cox.uni.p,  decreasing=FALSE)  ## smallest first
sum(cox.uni.p < 0.05) # 994
## [1] 1009
cox.top.unadjusted <- names(cox.uni.p)[cox.uni.p < 0.05]
adj.cox.uni.p <- p.adjust(cox.uni.p, method = "BH") # YZ: adjusted p-values
sum(adj.cox.uni.p < 0.05) # 7 -- Check how many significant
## [1] 27
(cox.top <- names(adj.cox.uni.p)[adj.cox.uni.p < 0.05]) # IDs of significant genes
##  [1] "DEFB114|245928"     "SLITRK3|22865"      "SNORD114-9|767585"
##  [4] "SNORD114-27|767608" "GALP|85569"         "OR4C13|283092"
##  [7] "ZPBP2|124626"       "LOC143188|143188"   "KRT34|3885"
## [10] "GALNT9|50614"       "OR4C46|119749"      "SI|6476"
## [13] "LHFPL3|375612"      "VN1R4|317703"       "OR51F1|256892"
## [16] "PWRN1|791114"       "NDST4|64579"        "LCE1A|353131"
## [19] "LCE3C|353144"       "BCHE|590"           "GPR123|84435"
## [22] "FAM26D|221301"      "STXBP5|134957"      "LOC387646|387646"
## [25] "VSIG8|391123"       "SPRR4|163778"       "GRIA3|2892"
(cox.top.p.adj <- adj.cox.uni.p[adj.cox.uni.p < 0.05])  # adjusted p-values of
significant genes
##     DEFB114|245928      SLITRK3|22865  SNORD114-9|767585
##       0.000000e+00       0.000000e+00       0.000000e+00
## SNORD114-27|767608         GALP|85569      OR4C13|283092
##       2.347895e-10       2.461196e-07       6.340525e-07
##       ZPBP2|124626   LOC143188|143188         KRT34|3885
##       1.384887e-06       2.300982e-06       2.327375e-06
##       GALNT9|50614      OR4C46|119749            SI|6476
##       2.395467e-06       4.337979e-06       1.230575e-05
##      LHFPL3|375612       VN1R4|317703      OR51F1|256892
##       1.917262e-05       1.978473e-05       8.914688e-05
##       PWRN1|791114        NDST4|64579       LCE1A|353131
```

```
##       7.574123e-04       8.729197e-04       1.740293e-03
##       LCE3C|353144           BCHE|590       GPR123|84435
##       2.495561e-03       4.513411e-03       6.787162e-03
##      FAM26D|221301      STXBP5|134957   LOC387646|387646
##       7.668875e-03       1.156933e-02       1.546932e-02
##       VSIG8|391123       SPRR4|163778         GRIA3|2892
##       3.021503e-02       4.134338e-02       4.134338e-02
write.csv(cox.top.p.adj, file =
"~/Desktop/ICIBM_2019/SignificantGenesFromUnivariateCoxModel.csv")
gene.cox.top.unadjusted = cox.top.unadjusted
#####################################################################
## LASSO starting with top univariate genes
#####################################################################

## penalized likelihood method using glmnet
set.seed(1000)

## Restrict starting to all significant genes based on univariate Cox model
## cv.glmnet does CV to determine the optimal lambda value
cv.lasso <- cv.glmnet(X.train[,cox.top], S.train, family = "cox", alpha=1)
plot(cv.lasso)
```

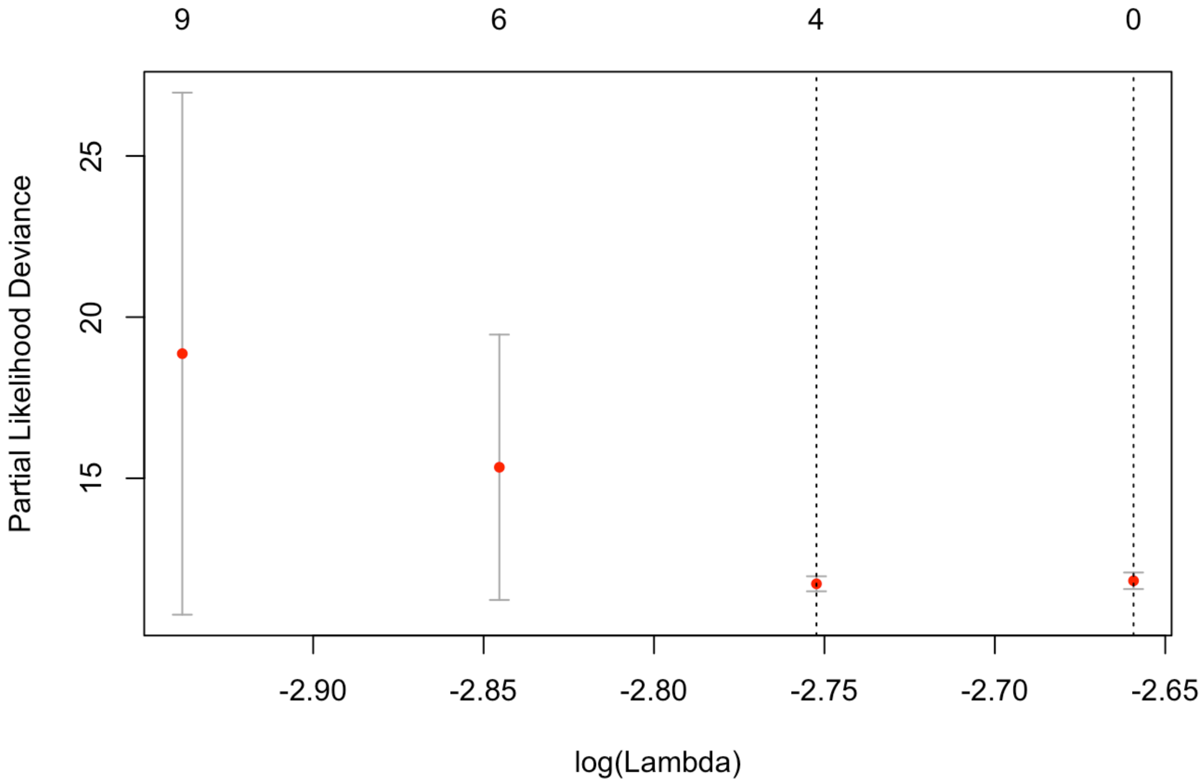


```
## How many predictors are selected at the optimal lambda value (lambda.min)?
coef.min <-  coef(cv.lasso, s = "lambda.min")
sum(coef.min != 0) # 3
## [1] 4
## lasso fit
coxfit.lasso <- glmnet(X.train[,cox.top], S.train, family = "cox", alpha=1)
plot(coxfit.lasso, xvar="lambda",label=TRUE)
abline(v=log(cv.lasso$lambda.min))
```

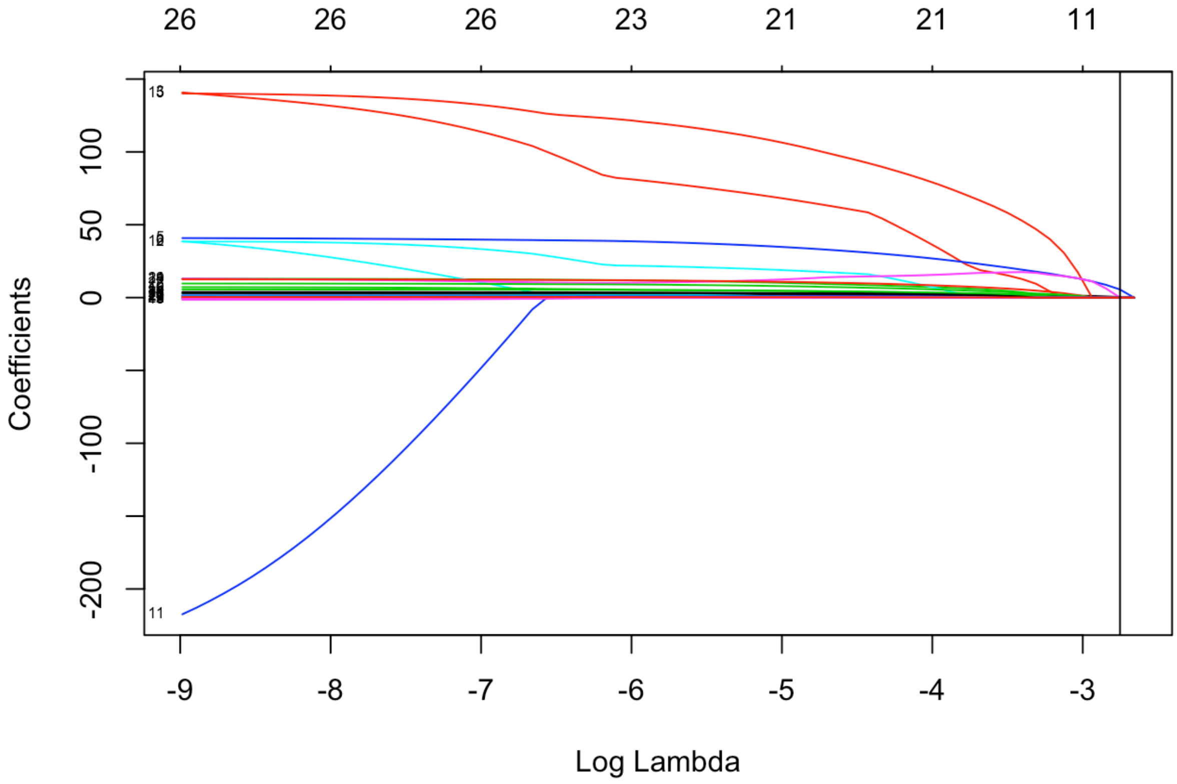


```
## genes selected with optimal model fitting
(coef.min <-  coef(cv.lasso, s = "lambda.min"))
## 27 x 1 sparse Matrix of class "dgCMatrix"
##                              1
## DEFB114|245928      .
## SLITRK3|22865       .
## SNORD114-9|767585   .
## SNORD114-27|767608 .
## GALP|85569          5.53109382
## OR4C13|283092       .
## ZPBP2|124626        .
## LOC143188|143188    0.40508713
## KRT34|3885          .
## GALNT9|50614        .
## OR4C46|119749       .
## SI|6476             .
## LHFPL3|375612       .
## VN1R4|317703        .
## OR51F1|256892       .
## PWRN1|791114        .
## NDST4|64579         .
## LCE1A|353131        .
## LCE3C|353144        .
## BCHE|590            .
## GPR123|84435        .
## FAM26D|221301       .
## STXBP5|134957       0.05918197
## LOC387646|387646    0.01009837
## VSIG8|391123        .
## SPRR4|163778        .
## GRIA3|2892          .
```

```
(active.min <- which(as.matrix(coef.min != 0)))
## [1]  5  8 23 24
(index.min <- coef.min[active.min])
## [1] 5.53109382 0.40508713 0.05918197 0.01009837
(sel.lasso <- rownames(coef.min)[active.min]) # Important
## [1] "GALP|85569"        "LOC143188|143188" "STXBP5|134957"
## [4] "LOC387646|387646"
#################################
## Test set validation - LASSO
#################################

## risk score
rs.lasso.train <- predict(cv.lasso, newx=X.train[,cox.top], s="lambda.min")
rs.lasso.test <- predict(cv.lasso, newx=X.test[,cox.top], s="lambda.min")

# ## median stratification
good.prog.lasso <- (rs.lasso.test < median(rs.lasso.train))
(fit.lasso <- survfit(S.test ~ good.prog.lasso))
## Call: survfit(formula = S.test ~ good.prog.lasso)
##
##                          n events median 0.95LCL 0.95UCL
## good.prog.lasso=FALSE 124     24   10.8    6.90      NA
## good.prog.lasso=TRUE  131     22   10.8    7.98      NA
# ## logrank test for validation set
# (logrank.lasso <- survdiff(S.test ~ good.prog.lasso))
(logrank.lasso <- survdiff(S.test ~ rs.lasso.test))
## Call:
## survdiff(formula = S.test ~ rs.lasso.test)
##
##                                   N Observed Expected (O-E)^2/E (O-E)^2/V
## rs.lasso.test=0.0489388987420405 1        0  0.03349  3.35e-02  3.37e-02
## rs.lasso.test=0.0500373697840996 1        1  0.28117  1.84e+00  1.86e+00
## rs.lasso.test=0.121008058209792  1        0  0.13125  1.31e-01  1.32e-01
## rs.lasso.test=0.12435890100359   1        0  0.13125  1.31e-01  1.32e-01
## rs.lasso.test=0.129204036018684  1        0  0.02797  2.80e-02  2.81e-02
## rs.lasso.test=0.129989019574271  1        0  0.19059  1.91e-01  1.92e-01
## rs.lasso.test=0.132810417571559  1        0  0.36818  3.68e-01  3.73e-01
## rs.lasso.test=0.133528769468514  1        0  0.89691  8.97e-01  9.41e-01
## rs.lasso.test=0.141667674697084  1        1  0.09008  9.19e+00  9.25e+00
## rs.lasso.test=0.142065122067192  1        0  1.09691  1.10e+00  1.19e+00
## rs.lasso.test=0.142466189411534  1        0  0.36818  3.68e-01  3.73e-01
## rs.lasso.test=0.14342180791951   1        0  0.00857  8.57e-03  8.60e-03
## rs.lasso.test=0.145372799014959  1        0  0.02259  2.26e-02  2.27e-02
## rs.lasso.test=0.145751837770487  1        0  0.00000       NaN       NaN
## rs.lasso.test=0.146011879706514  1        1  0.70600  1.22e-01  1.27e-01
## rs.lasso.test=0.146881242130203  1        0  0.26298  2.63e-01  2.66e-01
## rs.lasso.test=0.148591892428048  1        1  0.54574  3.78e-01  3.86e-01
## rs.lasso.test=0.149881862198661  1        0  0.00000       NaN       NaN
## rs.lasso.test=0.153040624015791  1        0  0.12224  1.22e-01  1.23e-01
## rs.lasso.test=0.153214671747257  1        1  0.09783  8.32e+00  8.37e+00
## rs.lasso.test=0.154588087846792  1        0  0.00000       NaN       NaN
## rs.lasso.test=0.155996238073375  1        0  0.03349  3.35e-02  3.37e-02
## rs.lasso.test=0.156213406265228  1        0  0.13125  1.31e-01  1.32e-01
## rs.lasso.test=0.157508520803106  1        0  0.10570  1.06e-01  1.06e-01
## rs.lasso.test=0.157735429886341  1        0  0.05338  5.34e-02  5.37e-02
## rs.lasso.test=0.157998997416851  1        0  0.03986  3.99e-02  4.01e-02
## rs.lasso.test=0.15922654425588   1        0  0.26298  2.63e-01  2.66e-01
## rs.lasso.test=0.16056236521749   1        0  0.00422  4.22e-03  4.24e-03
## rs.lasso.test=0.160567054700577  1        0  0.01764  1.76e-02  1.77e-02
## rs.lasso.test=0.162299510977209  1        0  0.46408  4.64e-01  4.72e-01
## rs.lasso.test=0.163214918650614  1        0  0.34318  3.43e-01  3.48e-01
## rs.lasso.test=0.165284020762894  1        1  0.02259  4.23e+01  4.25e+01
## rs.lasso.test=0.166342153552599  1        1  0.24760  2.29e+00  2.31e+00
## rs.lasso.test=0.16682253250407   1        0  0.02259  2.26e-02  2.27e-02
## rs.lasso.test=0.167738492671697  1        0  0.01764  1.76e-02  1.77e-02
```

```
## rs.lasso.test=0.169013882322792  1         0  0.00000       NaN       NaN
## rs.lasso.test=0.171691284493063  1         0  0.03986  3.99e-02  4.01e-02
## rs.lasso.test=0.172645203177344  1         0  0.29968  3.00e-01  3.03e-01
## rs.lasso.test=0.172855139884187  1         0  0.03349  3.35e-02  3.37e-02
## rs.lasso.test=0.174539190856266  1         0  0.13125  1.31e-01  1.32e-01
## rs.lasso.test=0.175805117580874  1         0  0.13125  1.31e-01  1.32e-01
## rs.lasso.test=0.175850434165723  1         0  0.15385  1.54e-01  1.55e-01
## rs.lasso.test=0.175970212044006  1         0  0.13125  1.31e-01  1.32e-01
## rs.lasso.test=0.176485273922827  1         0  0.26298  2.63e-01  2.66e-01
## rs.lasso.test=0.17746272275887   1         0  0.03349  3.35e-02  3.37e-02
## rs.lasso.test=0.178248888979254  1         0  0.13125  1.31e-01  1.32e-01
## rs.lasso.test=0.178544430386622  1         1  0.20429  3.10e+00  3.13e+00
## rs.lasso.test=0.178857784398616  1         0  0.39849  3.98e-01  4.04e-01
## rs.lasso.test=0.179058928448423  1         0  0.26298  2.63e-01  2.66e-01
## rs.lasso.test=0.179168924023883  1         0  0.01764  1.76e-02  1.77e-02
## rs.lasso.test=0.179648648653895  1         0  0.00857  8.57e-03  8.60e-03
## rs.lasso.test=0.179741876299884  1         0  0.03986  3.99e-02  4.01e-02
## rs.lasso.test=0.18042066143038   1         0  0.15385  1.54e-01  1.55e-01
## rs.lasso.test=0.180752509712253  1         0  0.89691  8.97e-01  9.41e-01
## rs.lasso.test=0.180818239948902  1         0  0.00000       NaN       NaN
## rs.lasso.test=0.18183612144346   1         0  0.03349  3.35e-02  3.37e-02
## rs.lasso.test=0.182041505473313  1         0  0.00422  4.22e-03  4.24e-03
## rs.lasso.test=0.185312762555332  1         0  0.02797  2.80e-02  2.81e-02
## rs.lasso.test=0.185367205306252  1         0  0.03349  3.35e-02  3.37e-02
## rs.lasso.test=0.185875178530404  1         0  1.09691  1.10e+00  1.19e+00
## rs.lasso.test=0.186661552771233  1         0  0.13125  1.31e-01  1.32e-01
## rs.lasso.test=0.188833890647933  1         0  0.00000       NaN       NaN
## rs.lasso.test=0.189649647064889  1         0  0.01764  1.76e-02  1.77e-02
## rs.lasso.test=0.189974973518205  1         0  0.08244  8.24e-02  8.30e-02
## rs.lasso.test=0.190338095034726  1         0  0.06746  6.75e-02  6.79e-02
## rs.lasso.test=0.190743399620788  1         0  0.13125  1.31e-01  1.32e-01
## rs.lasso.test=0.191937943501274  1         0  0.13125  1.31e-01  1.32e-01
## rs.lasso.test=0.192739827071659  1         0  0.13125  1.31e-01  1.32e-01
## rs.lasso.test=0.193468077849697  1         0  0.54574  5.46e-01  5.57e-01
## rs.lasso.test=0.193626929642892  1         0  0.00000       NaN       NaN
## rs.lasso.test=0.193943209205646  1         0  0.19059  1.91e-01  1.92e-01
## rs.lasso.test=0.194431391442882  1         1  0.14249  5.16e+00  5.20e+00
## rs.lasso.test=0.194486667631086  1         0  0.13125  1.31e-01  1.32e-01
## rs.lasso.test=0.195048869369794  1         0  0.23267  2.33e-01  2.35e-01
## rs.lasso.test=0.195113288388433  1         1  0.79691  5.18e-02  5.40e-02
## rs.lasso.test=0.195527506143973  1         0  0.00000       NaN       NaN
## rs.lasso.test=0.19622862003581   1         0  0.03986  3.99e-02  4.01e-02
## rs.lasso.test=0.196307458563523  1         0  0.46408  4.64e-01  4.72e-01
## rs.lasso.test=0.196527268813989  1         0  0.03349  3.35e-02  3.37e-02
## rs.lasso.test=0.197310950638654  1         0  0.03349  3.35e-02  3.37e-02
## rs.lasso.test=0.197459455500751  1         0  0.04653  4.65e-02  4.68e-02
## rs.lasso.test=0.197574831966904  1         1  0.00857  1.15e+02  1.15e+02
## rs.lasso.test=0.197576517881144  1         0  0.00422  4.22e-03  4.24e-03
## rs.lasso.test=0.198108657503884  1         1  0.08244  1.02e+01  1.03e+01
## rs.lasso.test=0.19927066769888   1         0  0.02259  2.26e-02  2.27e-02
## rs.lasso.test=0.199767840107871  1         0  0.02259  2.26e-02  2.27e-02
## rs.lasso.test=0.200192401346383  1         0  0.02259  2.26e-02  2.27e-02
## rs.lasso.test=0.200514665336794  1         0  0.19059  1.91e-01  1.92e-01
## rs.lasso.test=0.200522009845737  1         1  0.62267  2.29e-01  2.35e-01
## rs.lasso.test=0.200866156780956  1         1  0.36818  1.08e+00  1.10e+00
## rs.lasso.test=0.201048010970562  1         0  0.06746  6.75e-02  6.79e-02
## rs.lasso.test=0.201404914781368  1         0  0.04653  4.65e-02  4.68e-02
## rs.lasso.test=0.201519194374877  1         1  0.00422  2.35e+02  2.36e+02
## rs.lasso.test=0.201847211531919  1         0  0.01764  1.76e-02  1.77e-02
## rs.lasso.test=0.202210187429164  1         0  0.54574  5.46e-01  5.57e-01
## rs.lasso.test=0.203064201926967  1         0  0.26298  2.63e-01  2.66e-01
## rs.lasso.test=0.20332819223001   1         0  0.03349  3.35e-02  3.37e-02
## rs.lasso.test=0.203948661166735  1         0  0.19059  1.91e-01  1.92e-01
## rs.lasso.test=0.204733937116338  1         0  0.03349  3.35e-02  3.37e-02
## rs.lasso.test=0.20483758864659   1         0  0.01764  1.76e-02  1.77e-02
```

```
## rs.lasso.test=0.205420431148773  1         0  0.06746  6.75e-02  6.79e-02
## rs.lasso.test=0.205439792726026  1         0  0.36818  3.68e-01  3.73e-01
## rs.lasso.test=0.206591819133884  1         0  0.17809  1.78e-01  1.80e-01
## rs.lasso.test=0.206690331857409  1         0  0.19059  1.91e-01  1.92e-01
## rs.lasso.test=0.207187521486264  1         1  0.03986  2.31e+01  2.32e+01
## rs.lasso.test=0.20829793386136   1         0  0.54574  5.46e-01  5.57e-01
## rs.lasso.test=0.208837576913958  1         1  0.34318  1.26e+00  1.27e+00
## rs.lasso.test=0.210588636811225  1         0  0.26298  2.63e-01  2.66e-01
## rs.lasso.test=0.212265186939448  1         0  0.03986  3.99e-02  4.01e-02
## rs.lasso.test=0.212734078373254  1         0  0.01303  1.30e-02  1.31e-02
## rs.lasso.test=0.213581647202953  1         0  0.54574  5.46e-01  5.57e-01
## rs.lasso.test=0.21421820430229   1         0  0.01764  1.76e-02  1.77e-02
## rs.lasso.test=0.214751781511046  1         1  0.03349  2.79e+01  2.80e+01
## rs.lasso.test=0.214912712670891  1         0  0.00000       NaN       NaN
## rs.lasso.test=0.215219656663062  1         0  0.03349  3.35e-02  3.37e-02
## rs.lasso.test=0.215579477790333  1         0  0.19059  1.91e-01  1.92e-01
## rs.lasso.test=0.217121996001159  1         0  0.09783  9.78e-02  9.85e-02
## rs.lasso.test=0.217148700286935  1         0  0.00422  4.22e-03  4.24e-03
## rs.lasso.test=0.217176201798102  1         0  0.34318  3.43e-01  3.48e-01
## rs.lasso.test=0.21733475163208   1         0  0.00000       NaN       NaN
## rs.lasso.test=0.217472101192051  1         1  0.13125  5.75e+00  5.79e+00
## rs.lasso.test=0.217915141740206  1         0  0.12224  1.22e-01  1.23e-01
## rs.lasso.test=0.217983535899486  1         1  0.23267  2.53e+00  2.55e+00
## rs.lasso.test=0.218001120125837  1         0  0.89691  8.97e-01  9.41e-01
## rs.lasso.test=0.21803082118212   1         0  0.02259  2.26e-02  2.27e-02
## rs.lasso.test=0.218788929765042  1         0  0.13125  1.31e-01  1.32e-01
## rs.lasso.test=0.219095945903541  1         0  0.34318  3.43e-01  3.48e-01
## rs.lasso.test=0.219497420659798  1         1  0.05338  1.68e+01  1.69e+01
## rs.lasso.test=0.219575089055362  1         0  0.03986  3.99e-02  4.01e-02
## rs.lasso.test=0.219845817409558  1         1  0.06037  1.46e+01  1.47e+01
## rs.lasso.test=0.220273720737385  1         0  0.36818  3.68e-01  3.73e-01
## rs.lasso.test=0.220964823521175  1         0  0.02797  2.80e-02  2.81e-02
## rs.lasso.test=0.221214823451238  1         0  0.13125  1.31e-01  1.32e-01
## rs.lasso.test=0.221222034859794  1         0  0.04653  4.65e-02  4.68e-02
## rs.lasso.test=0.2217489602820 3  1         0  0.12224  1.22e-01  1.23e-01
## rs.lasso.test=0.221976419114532  1         1  0.50408  4.88e-01  4.97e-01
## rs.lasso.test=0.223292240438507  1         0  0.29968  3.00e-01  3.03e-01
## rs.lasso.test=0.223305142416043  1         1  0.02797  3.38e+01  3.39e+01
## rs.lasso.test=0.224433781915838  1         0  0.00857  8.57e-03  8.60e-03
## rs.lasso.test=0.225254409102524  1         0  0.01303  1.30e-02  1.31e-02
## rs.lasso.test=0.225329981194086  1         0  0.03349  3.35e-02  3.37e-02
## rs.lasso.test=0.225334476398482  1         0  0.03349  3.35e-02  3.37e-02
## rs.lasso.test=0.225400811213722  1         0  0.54574  5.46e-01  5.57e-01
## rs.lasso.test=0.226158536224011  1         0  0.13125  1.31e-01  1.32e-01
## rs.lasso.test=0.226220157489476  1         1  0.19059  3.44e+00  3.47e+00
## rs.lasso.test=0.226853914778223  1         0  0.02259  2.26e-02  2.27e-02
## rs.lasso.test=0.226952940347732  1         0  0.54574  5.46e-01  5.57e-01
## rs.lasso.test=0.227746959021586  1         0  0.00000       NaN       NaN
## rs.lasso.test=0.228052575087917  1         0  0.29968  3.00e-01  3.03e-01
## rs.lasso.test=0.228147416572592  1         0  0.54574  5.46e-01  5.57e-01
## rs.lasso.test=0.228434131286286  1         0  0.10570  1.06e-01  1.06e-01
## rs.lasso.test=0.228932909373242  1         0  0.21818  2.18e-01  2.20e-01
## rs.lasso.test=0.229389083540886  1         1  0.21818  2.80e+00  2.83e+00
## rs.lasso.test=0.231836848242816  1         0  0.12224  1.22e-01  1.23e-01
## rs.lasso.test=0.232132178054164  1         0  0.06746  6.75e-02  6.79e-02
## rs.lasso.test=0.232402749262684  1         0  0.89691  8.97e-01  9.41e-01
## rs.lasso.test=0.23485522560292   1         0  0.13125  1.31e-01  1.32e-01
## rs.lasso.test=0.236029137847206  1         0  0.13125  1.31e-01  1.32e-01
## rs.lasso.test=0.236047359685642  1         0  0.01764  1.76e-02  1.77e-02
## rs.lasso.test=0.236256938727861  1         0  0.11377  1.14e-01  1.15e-01
## rs.lasso.test=0.238809739665292  1         0  0.01764  1.76e-02  1.77e-02
## rs.lasso.test=0.239643497345054  1         1  0.26298  2.07e+00  2.09e+00
## rs.lasso.test=0.239654830515233  1         0  0.01764  1.76e-02  1.77e-02
## rs.lasso.test=0.240571226392332  1         1  0.07492  1.14e+01  1.15e+01
## rs.lasso.test=0.240587216414255  1         0  0.11377  1.14e-01  1.15e-01
```

```
## rs.lasso.test=0.241190531183302  1         0  0.05338  5.34e-02  5.37e-02
## rs.lasso.test=0.241533725720472  1         0  0.29968  3.00e-01  3.03e-01
## rs.lasso.test=0.241667948137617  1         0  0.03349  3.35e-02  3.37e-02
## rs.lasso.test=0.243857508184109  1         0  0.00422  4.22e-03  4.24e-03
## rs.lasso.test=0.246192962364062  1         0  0.03349  3.35e-02  3.37e-02
## rs.lasso.test=0.246616806123982  1         0  0.02259  2.26e-02  2.27e-02
## rs.lasso.test=0.246714916681564  1         1  2.09691  5.74e-01  1.19e+00
## rs.lasso.test=0.247194627396635  1         0  0.00000       NaN       NaN
## rs.lasso.test=0.248648377964609  1         0  0.06746  6.75e-02  6.79e-02
## rs.lasso.test=0.249300245552353  1         1  0.01764  5.47e+01  5.50e+01
## rs.lasso.test=0.249520094279397  1         0  0.01764  1.76e-02  1.77e-02
## rs.lasso.test=0.24962464993318   1         0  0.12224  1.22e-01  1.23e-01
## rs.lasso.test=0.251374261827493  1         0  0.00000       NaN       NaN
## rs.lasso.test=0.251419301578526  1         0  0.21818  2.18e-01  2.20e-01
## rs.lasso.test=0.251889952286334  1         0  0.00000       NaN       NaN
## rs.lasso.test=0.252707977520521  1         0  0.54574  5.46e-01  5.57e-01
## rs.lasso.test=0.254476521010903  1         0  0.29968  3.00e-01  3.03e-01
## rs.lasso.test=0.254481206965532  1         0  0.01764  1.76e-02  1.77e-02
## rs.lasso.test=0.254717410523303  1         0  0.01303  1.30e-02  1.31e-02
## rs.lasso.test=0.255755838311583  1         0  0.54574  5.46e-01  5.57e-01
## rs.lasso.test=0.256232692228291  1         1  0.39849  9.08e-01  9.21e-01
## rs.lasso.test=0.256246636892325  1         0  0.02259  2.26e-02  2.27e-02
## rs.lasso.test=0.256431218447177  1         0  0.46408  4.64e-01  4.72e-01
## rs.lasso.test=0.2567312964462    1         0  0.02259  2.26e-02  2.27e-02
## rs.lasso.test=0.256819576538945  1         0  0.01303  1.30e-02  1.31e-02
## rs.lasso.test=0.257128212805373  1         0  0.02259  2.26e-02  2.27e-02
## rs.lasso.test=0.258325910986679  1         0  0.15385  1.54e-01  1.55e-01
## rs.lasso.test=0.260918336591939  1         0  0.01303  1.30e-02  1.31e-02
## rs.lasso.test=0.263294672724629  1         0  0.46408  4.64e-01  4.72e-01
## rs.lasso.test=0.264820788592873  1         0  0.03349  3.35e-02  3.37e-02
## rs.lasso.test=0.265159737069219  1         1  0.46408  6.19e-01  6.30e-01
## rs.lasso.test=0.26653261223555   1         0  0.06037  6.04e-02  6.07e-02
## rs.lasso.test=0.268965293424704  1         0  0.03349  3.35e-02  3.37e-02
## rs.lasso.test=0.269570040410673  1         0  0.03349  3.35e-02  3.37e-02
## rs.lasso.test=0.270792175314182  1         0  0.36818  3.68e-01  3.73e-01
## rs.lasso.test=0.274146785009595  1         0  0.11377  1.14e-01  1.15e-01
## rs.lasso.test=0.274467711878335  1         0  0.01303  1.30e-02  1.31e-02
## rs.lasso.test=0.275528546584049  1         1  0.15385  4.65e+00  4.69e+00
## rs.lasso.test=0.276719038943319  1         0  0.03349  3.35e-02  3.37e-02
## rs.lasso.test=0.278083904620332  1         0  0.26298  2.63e-01  2.66e-01
## rs.lasso.test=0.278508413468172  1         0  0.12224  1.22e-01  1.23e-01
## rs.lasso.test=0.279319607710103  1         1  0.32096  1.44e+00  1.45e+00
## rs.lasso.test=0.279447090502177  1         0  0.15385  1.54e-01  1.55e-01
## rs.lasso.test=0.281613811795759  1         1  0.12224  6.30e+00  6.35e+00
## rs.lasso.test=0.281887182322233  1         0  0.03349  3.35e-02  3.37e-02
## rs.lasso.test=0.285983025682603  1         0  0.54574  5.46e-01  5.57e-01
## rs.lasso.test=0.286139687010455  1         0  0.13125  1.31e-01  1.32e-01
## rs.lasso.test=0.286331612461661  1         0  0.02797  2.80e-02  2.81e-02
## rs.lasso.test=0.287529868424565  1         0  0.00422  4.22e-03  4.24e-03
## rs.lasso.test=0.289095071517197  1         0  0.13125  1.31e-01  1.32e-01
## rs.lasso.test=0.289817123738598  1         0  0.13125  1.31e-01  1.32e-01
## rs.lasso.test=0.292060138365598  1         0  1.09691  1.10e+00  1.19e+00
## rs.lasso.test=0.292956749043778  1         1  0.06746  1.29e+01  1.30e+01
## rs.lasso.test=0.294170069671623  1         0  0.00857  8.57e-03  8.60e-03
## rs.lasso.test=0.294968355347607  1         0  0.13125  1.31e-01  1.32e-01
## rs.lasso.test=0.297174447798394  1         1  0.17809  3.79e+00  3.82e+00
## rs.lasso.test=0.297509530484678  1         0  0.00000       NaN       NaN
## rs.lasso.test=0.299345903044398  1         0  0.00857  8.57e-03  8.60e-03
## rs.lasso.test=0.300545888330455  1         1  0.01303  7.48e+01  7.51e+01
## rs.lasso.test=0.301509811208857  1         0  0.00000       NaN       NaN
## rs.lasso.test=0.303576246396432  1         0  0.11377  1.14e-01  1.15e-01
## rs.lasso.test=0.3047429489414    1         1  0.43074  7.52e-01  7.64e-01
## rs.lasso.test=0.30509067855275   1         0  0.03349  3.35e-02  3.37e-02
## rs.lasso.test=0.305996530674904  1         0  0.26298  2.63e-01  2.66e-01
## rs.lasso.test=0.306015700681064  1         0  0.00000       NaN       NaN
```

```
## rs.lasso.test=0.306628406624266  1        0  0.34318  3.43e-01  3.48e-01
## rs.lasso.test=0.307397290656214  1        1  0.16590  4.19e+00  4.23e+00
## rs.lasso.test=0.308461497958489  1        0  0.03349  3.35e-02  3.37e-02
## rs.lasso.test=0.310215353257311  1        0  0.03349  3.35e-02  3.37e-02
## rs.lasso.test=0.310347580513983  1        1  0.89691  1.18e-02  1.24e-02
## rs.lasso.test=0.323270484982868  1        1  1.09691  8.56e-03  9.26e-03
## rs.lasso.test=0.323961098550462  1        0  0.11377  1.14e-01  1.15e-01
## rs.lasso.test=0.327287957081033  1        1  0.29968  1.64e+00  1.65e+00
## rs.lasso.test=0.329586703421212  1        0  0.24760  2.48e-01  2.50e-01
## rs.lasso.test=0.343975402009102  1        0  0.00000       NaN       NaN
## rs.lasso.test=0.351306824221411  1        1  0.10570  7.57e+00  7.62e+00
## rs.lasso.test=0.356713456890969  1        0  0.02259  2.26e-02  2.27e-02
## rs.lasso.test=0.359021300612728  1        0  0.02259  2.26e-02  2.27e-02
## rs.lasso.test=0.362969256342261  1        0  0.32096  3.21e-01  3.25e-01
## rs.lasso.test=0.382700577969247  1        0  0.01764  1.76e-02  1.77e-02
## rs.lasso.test=0.383761756256206  1        0  0.26298  2.63e-01  2.66e-01
## rs.lasso.test=0.392229826184307  1        1  0.04653  1.95e+01  1.96e+01
## rs.lasso.test=0.404323048551692  1        0  0.09008  9.01e-02  9.06e-02
## rs.lasso.test=0.415573686914135  1        0  0.02259  2.26e-02  2.27e-02
## rs.lasso.test=0.439346612828938  1        0  0.06746  6.75e-02  6.79e-02
## rs.lasso.test=0.441491877740739  1        0  0.02259  2.26e-02  2.27e-02
## rs.lasso.test=0.581116410346827  1        0  0.29968  3.00e-01  3.03e-01
## rs.lasso.test=0.592898201018593  1        0  0.36818  3.68e-01  3.73e-01
## rs.lasso.test=2.02305430043759   1        1  0.11377  6.90e+00  6.95e+00
## rs.lasso.test=2.0639538979185    1        0  0.01764  1.76e-02  1.77e-02
##
##  Chisq= 853  on 236 degrees of freedom, p= <2e-16
(p.median.lasso <- 1-pchisq(logrank.lasso$chisq, 1))
## [1] 0
## cox regression on risk score
#cox.lasso <- coxph(S.test ~ good.prog.lasso)
cox.lasso <- coxph(S.test ~ rs.lasso.test)
summary(cox.lasso)
## Call:
## coxph(formula = S.test ~ rs.lasso.test)
##
##   n= 255, number of events= 46
##
##                 coef exp(coef) se(coef)     z Pr(>|z|)
## rs.lasso.test 1.1200    3.0648   0.5663 1.978   0.0479 *
## ---
## Signif. codes:  0 '***' 0.001 '**' 0.01 '*' 0.05 '.' 0.1 ' ' 1
##
##               exp(coef) exp(-coef) lower .95 upper .95
## rs.lasso.test     3.065     0.3263      1.01     9.298
##
## Concordance= 0.546  (se = 0.049 )
## Likelihood ratio test= 2.43  on 1 df,   p=0.1
## Wald test            = 3.91  on 1 df,   p=0.05
## Score (logrank) test = 4.55  on 1 df,   p=0.03
## C-index
## C-index assessment - for two selected individuals, the probability that the one
with the higher score (shorter survival) will actually have the shorter survival time.
Good biomarkers have indexes between 0.7 - 0.8.
#(c.lasso <- rcorrcens(S.test ~ good.prog.lasso)[,"C"])
(c.lasso <- rcorrcens(S.test ~ rs.lasso.test)[,"C"]) # 0.4539849
## [1] 0.4539849
## kaplan-meier curves
plot(fit.lasso, lwd = 2, lty = c(1,1), col = c("red","blue"), xlab = 'Time (years)',
     ylab = 'Estimated Survival Function')
legend("topright", legend=c('high risk', 'low risk'), lty = c(1,1),
       col = c("red", "blue"), lwd = 2, bty = "n")
text(6,0.9,paste("p =",round(p.median.lasso,3)))
text(6,0.85,paste("C =",round(c.lasso,3)))
title("Kaplan-Meier Curves, Lasso w/screen")
```

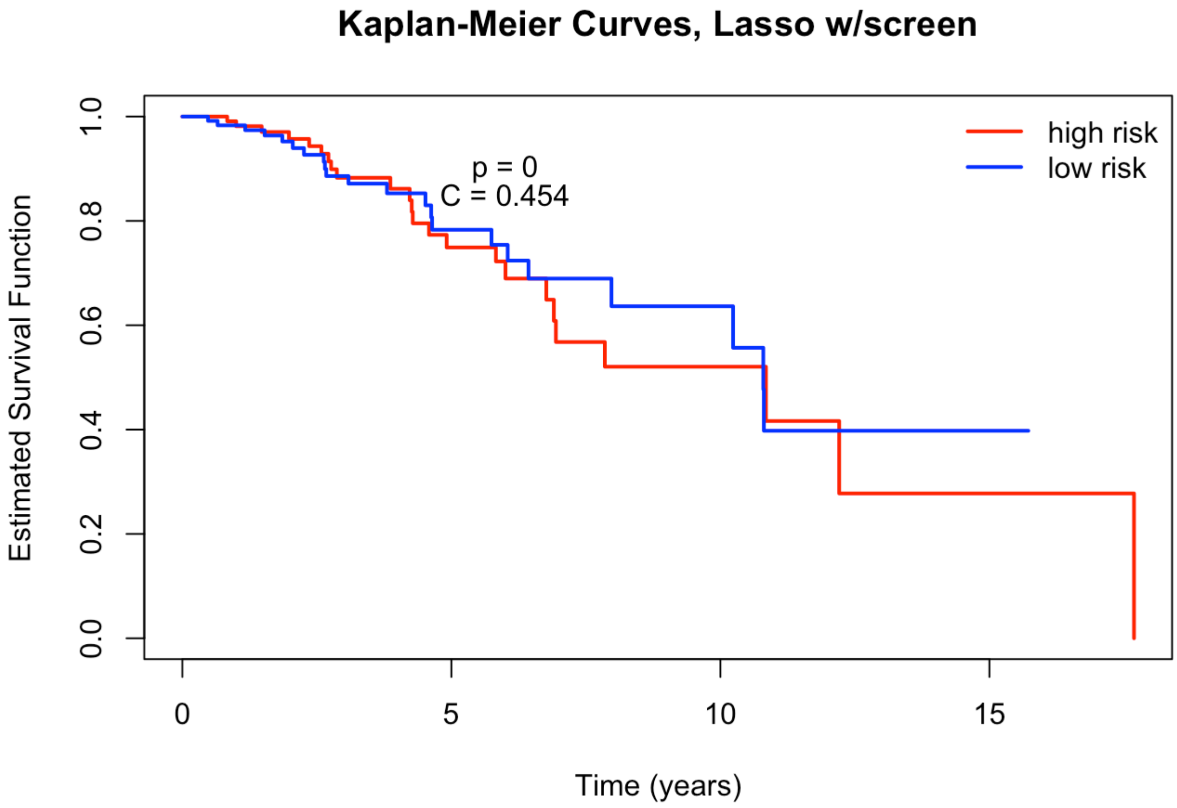


```
##########################################################
## YZ newly added code for ctree - YZ 20180628
##########################################################

#install.packages("partykit")
#install.packages("coin")
library("partykit")
## Loading required package: grid
## Loading required package: libcoin
## Loading required package: mvtnorm
##
## Attaching package: 'partykit'
## The following object is masked from 'package:IRanges':
##
##     width
## The following object is masked from 'package:S4Vectors':
##
##     width
## The following object is masked from 'package:BiocGenerics':
##
##     width
library("coin")

##Convert all data frame character columns to factors
temp <- surdata3
temp$pathologic_stage <- substring(surdata3$pathologic_stage, 7)
surdata4 <- as.data.frame(unclass(temp[, -1]))
rm(temp)

# BRCA_ctree1 <- ctree(Surv(survival.years, vital_status) ~ pathologic_stage +
pathology_T_stage + pathology_N_stage + pathology_M_stage
```

```
#                       + gender + radiation_therapy + histological_type +
number_of_lymph_nodes
#                       + race + ethnicity, data = surdata4)
# plot(BRCA_ctree1)

## Consider expression only
BRCA_ctree2 <- ctree(Surv(survival.years, vital_status) ~ .,
                     data = data.frame(survival.years=surdata4$survival.years,
vital_status=surdata4$vital_status, RNA.counts.01[, sel.lasso]))
plot(BRCA_ctree2)
```

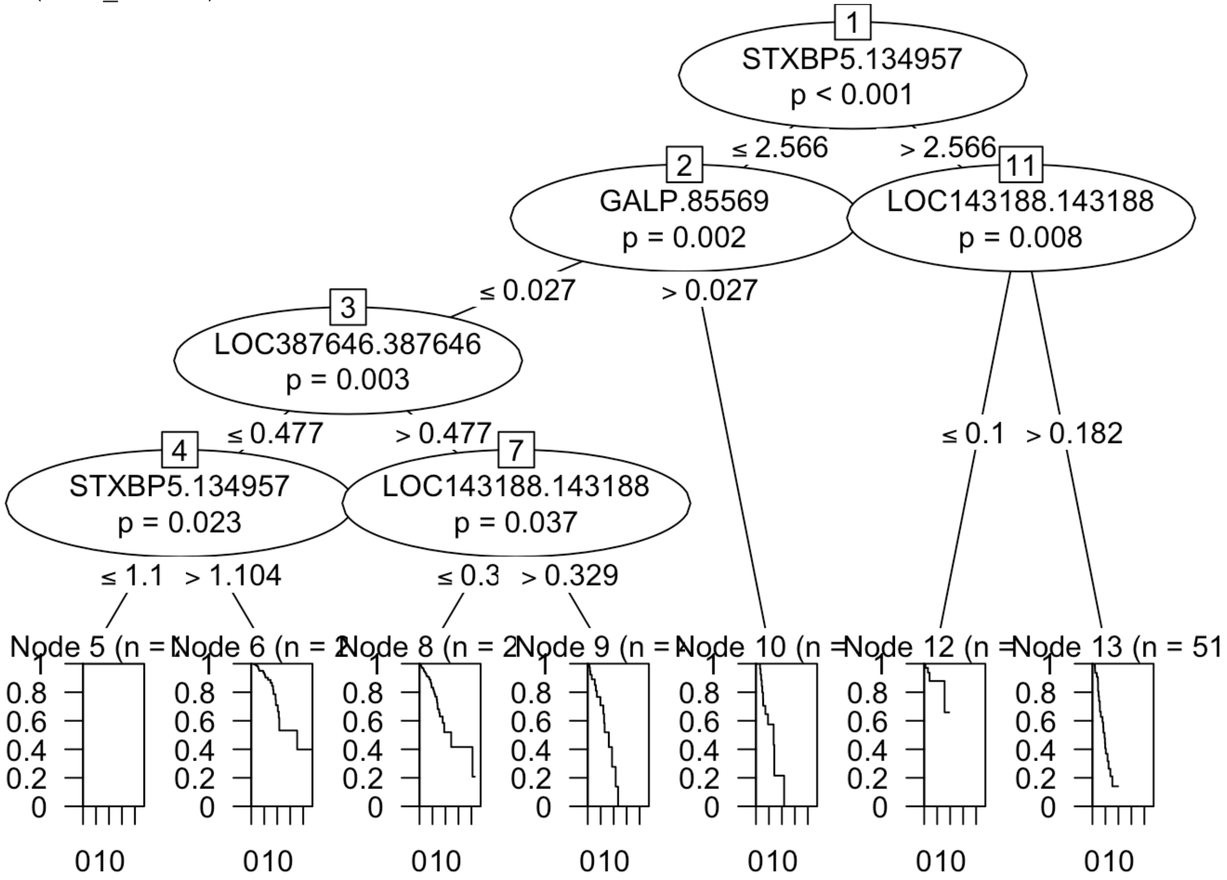


```
## Consider expression and clinical variables
BRCA_ctree3 <- ctree(Surv(survival.years, vital_status) ~ .,
                     data = data.frame(survival.years=surdata4$survival.years,
vital_status=surdata4$vital_status,
                                       pathologic_stage=surdata4$pathologic_stage,
gender=surdata4$gender,
                                       radiation_therapy=surdata4$radiation_therapy,
histological_type=surdata4$histological_type,
                                       race=surdata4$race,
ethnicity=surdata4$ethnicity,
                                       RNA.counts.01[, sel.lasso]))
plot(BRCA_ctree3)
```

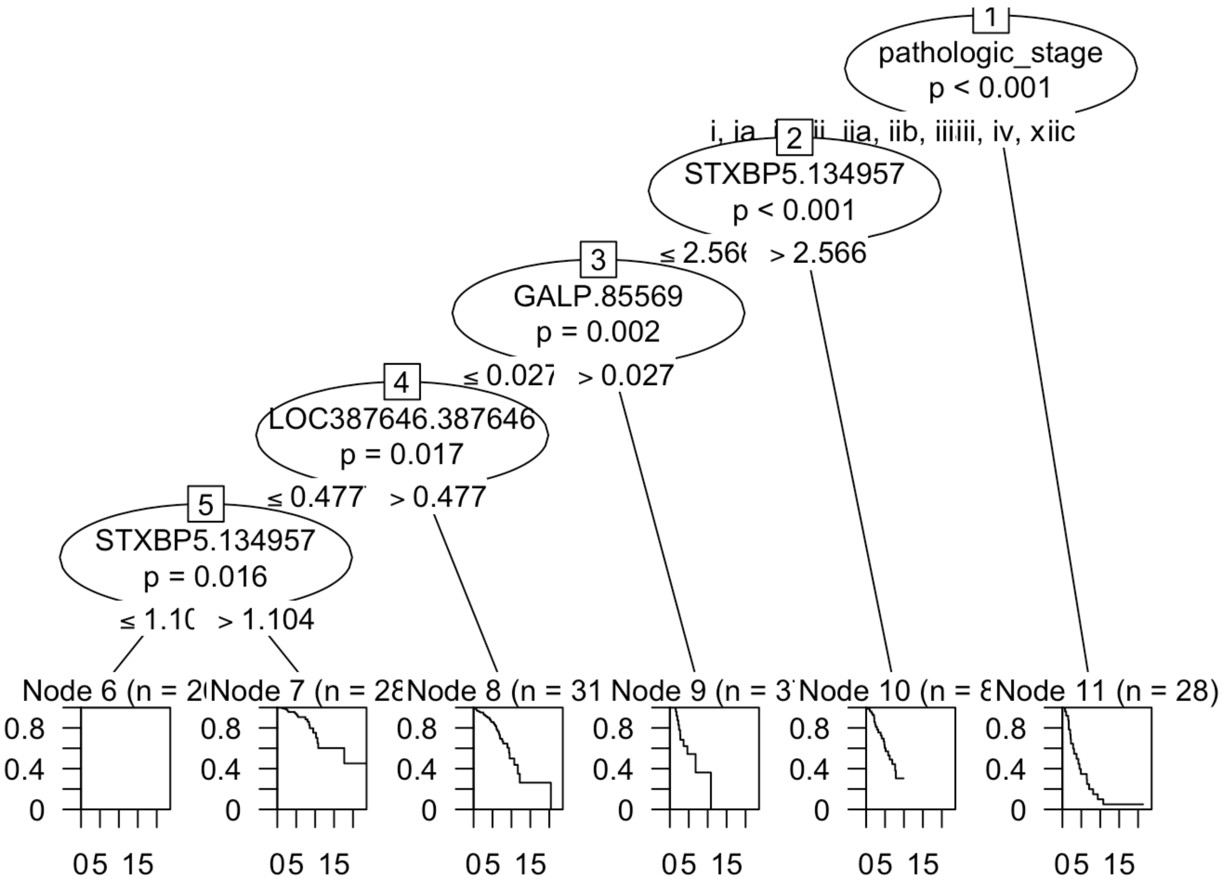

Visualize individual genes in TCGA BRCA

"STXBP5|134957", not significant, logrank test p=0.7

```
cox.STXBP5 <- coxph(Surv(survival.years, vital_status) ~
RNA.counts.01[,"STXBP5|134957"], data = surdata3)
summary(cox.STXBP5)
## Call:
## coxph(formula = Surv(survival.years, vital_status) ~ RNA.counts.01[,
##     "STXBP5|134957"], data = surdata3)
##
##   n= 765, number of events= 118
##
##                                   coef exp(coef) se(coef)     z Pr(>|z|)
## RNA.counts.01[, "STXBP5|134957"] 0.7575    2.1329   0.1608 4.711 2.47e-06
##
## RNA.counts.01[, "STXBP5|134957"] ***
## ---
## Signif. codes:  0 '***' 0.001 '**' 0.01 '*' 0.05 '.' 0.1 ' ' 1
##
##                                 exp(coef) exp(-coef) lower .95 upper .95
## RNA.counts.01[, "STXBP5|134957"]     2.133     0.4688     1.556     2.923
##
## Concordance= 0.598  (se = 0.034 )
## Likelihood ratio test= 20.8  on 1 df,   p=5e-06
## Wald test            = 22.19  on 1 df,   p=2e-06
## Score (logrank) test = 21.71  on 1 df,   p=3e-06
## Split at median expression of STXBP5
STXBP5.grps <- ifelse(RNA.counts.01[,"STXBP5|134957"] >
median(RNA.counts.01[,"STXBP5|134957"]), 1, 0)
table(STXBP5.grps)
## STXBP5.grps
```

```
##   0   1
## 383 382
km.STXBP5 <- survfit(Surv(survival.years, vital_status) ~ STXBP5.grps, data =
surdata3)
plot(km.STXBP5, col = 1:2, xlab = "Time (years)",  ylab = "Survival Probability",
     lwd = 2, main = paste("STXBP5 Expression and Survival"))
leg.txt <- c("Below median", "Above median")
legend("topright", leg.txt, lty = 1, col = 1:2, bty = "n", lwd = 2, title = "STXBP5
Expression")
```

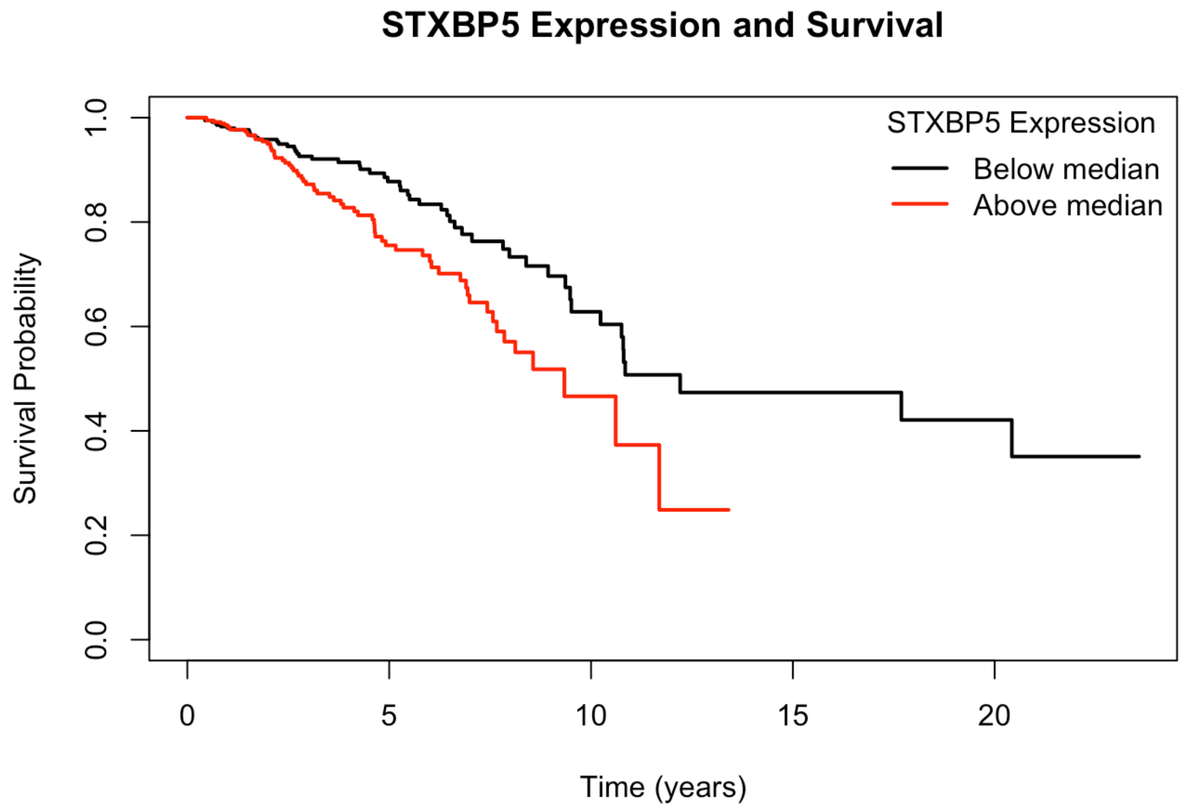

“GALP|85569”, very good separation, logrank test p=9e-04

```
cox.GALP <- coxph(Surv(survival.years, vital_status) ~ RNA.counts.01[,"GALP|85569"],
data = surdata3)
summary(cox.GALP)
## Call:
## coxph(formula = Surv(survival.years, vital_status) ~ RNA.counts.01[,
##     "GALP|85569"], data = surdata3)
##
##   n= 765, number of events= 118
##
##                                  coef exp(coef)  se(coef)     z Pr(>|z|)
## RNA.counts.01[, "GALP|85569"]     9.445 12643.208     2.015 4.688 2.76e-06
##
## RNA.counts.01[, "GALP|85569"] ***
## ---
## Signif. codes:  0 '***' 0.001 '**' 0.01 '*' 0.05 '.' 0.1 ' ' 1
##
##                               exp(coef) exp(-coef) lower .95 upper .95
## RNA.counts.01[, "GALP|85569"]     12643  7.909e-05     243.7    655819
##
## Concordance= 0.529  (se = 0.021 )
## Likelihood ratio test= 11.14  on 1 df,   p=8e-04
```

```
## Wald test            = 21.98  on 1 df,   p=3e-06
## Score (logrank) test = 30.33  on 1 df,   p=4e-08
## Split at median expression of VN1R4
GALP.grps <- ifelse(RNA.counts.01[,"GALP|85569"] >
median(RNA.counts.01[,"GALP|85569"]), 1, 0)
table(GALP.grps)
## GALP.grps
##   0   1
## 662 103
km.GALP <- survfit(Surv(survival.years, vital_status) ~ GALP.grps, data = surdata3)
plot(km.GALP, col = 1:2, xlab = "Time (years)",  ylab = "Survival Probability",
     lwd = 2, main = paste("GALP Expression and Survival"))
leg.txt <- c("Below median", "Above median")
legend("topright", leg.txt, lty = 1, col = 1:2, bty = "n", lwd = 2, title = "GALP
Expression")
```

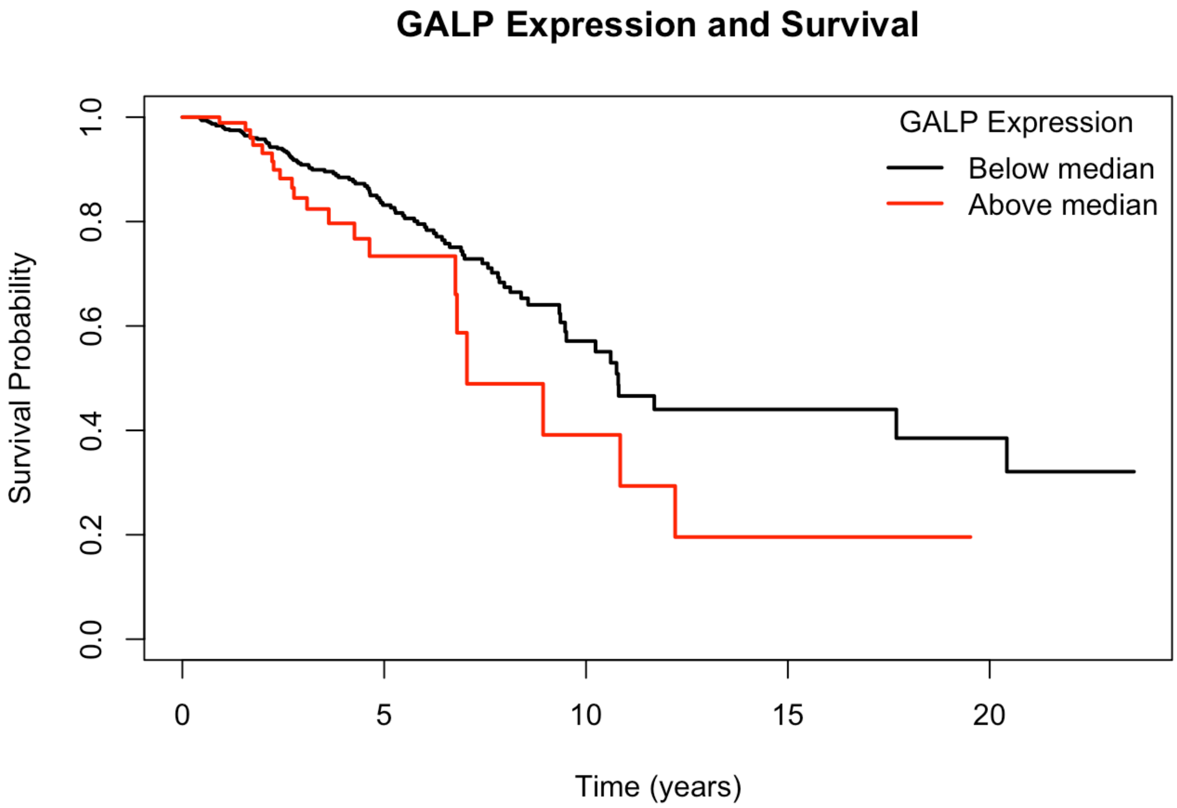

"LOC387646|387646", very good separation, logrank test p=6e-08

```
cox.LOC387646 <- coxph(Surv(survival.years, vital_status) ~
RNA.counts.01[,"LOC387646|387646"], data = surdata3)
summary(cox.LOC387646)
## Call:
## coxph(formula = Surv(survival.years, vital_status) ~ RNA.counts.01[,
##     "LOC387646|387646"], data = surdata3)
##
##   n= 765, number of events= 118
##
##                                        coef exp(coef) se(coef)     z
## RNA.counts.01[, "LOC387646|387646"] 0.5439    1.7226   0.1675 3.247
##                                    Pr(>|z|)
## RNA.counts.01[, "LOC387646|387646"]  0.00117 **
## ---
## Signif. codes:  0 '***' 0.001 '**' 0.01 '*' 0.05 '.' 0.1 ' ' 1
```

```
##
##                                    exp(coef) exp(-coef) lower .95
## RNA.counts.01[, "LOC387646|387646"]     1.723     0.5805     1.241
##                                    upper .95
## RNA.counts.01[, "LOC387646|387646"]     2.392
##
## Concordance= 0.612  (se = 0.028 )
## Likelihood ratio test= 8.96  on 1 df,   p=0.003
## Wald test            = 10.54  on 1 df,   p=0.001
## Score (logrank) test = 10.62  on 1 df,   p=0.001
## Split at median expression of LOC387646
LOC387646.grps <- ifelse(RNA.counts.01[,"LOC387646|387646"] >
median(RNA.counts.01[,"LOC387646|387646"]), 1, 0)
table(LOC387646.grps)
## LOC387646.grps
##   0   1
## 383 382
km.LOC387646 <- survfit(Surv(survival.years, vital_status) ~ LOC387646.grps, data =
surdata3)
plot(km.LOC387646, col = 1:2, xlab = "Time (years)",  ylab = "Survival Probability",
     lwd = 2, main = paste("LOC387646 Expression and Survival"))
leg.txt <- c("Below median", "Above median")
legend("topright", leg.txt, lty = 1, col = 1:2, bty = "n", lwd = 2, title = "LOC387646
Expression")
```

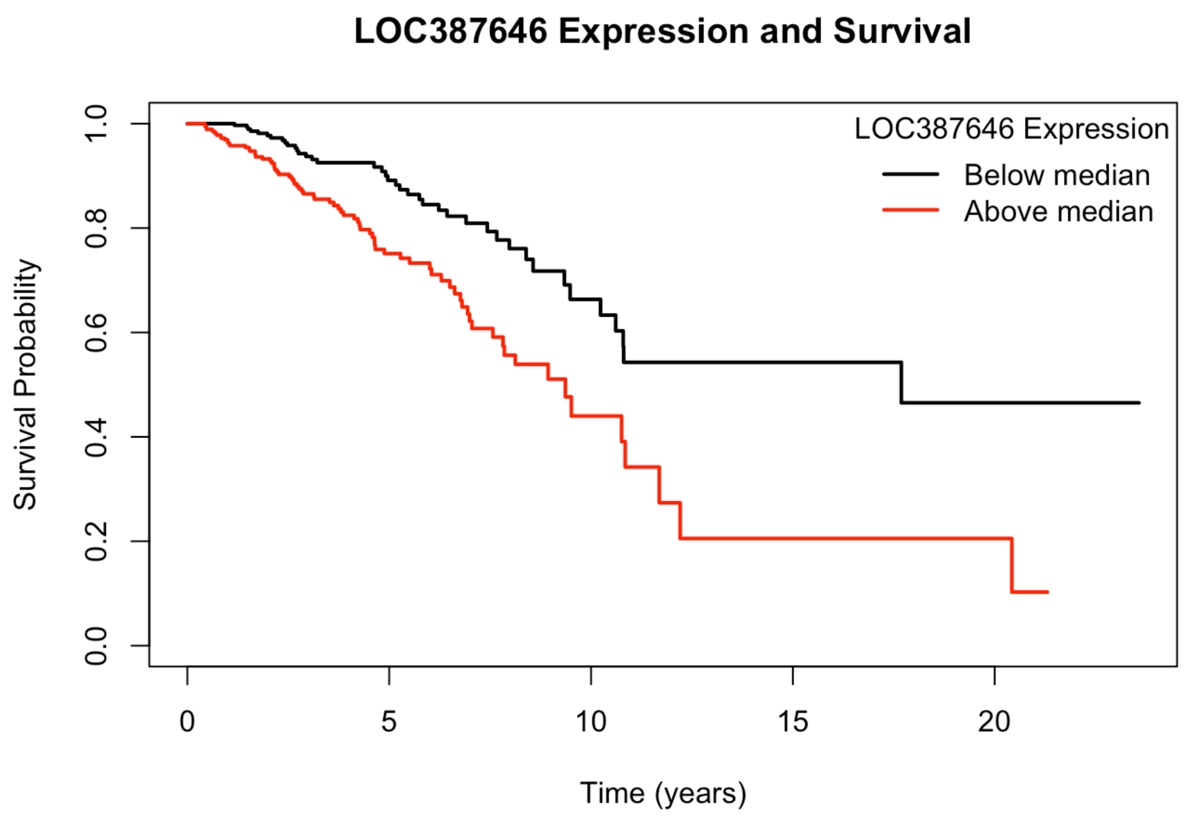

"LOC143188|143188", very good separation, logrank test p=6e-08

```
cox.LOC143188 <- coxph(Surv(survival.years, vital_status) ~
RNA.counts.01[,"LOC143188|143188"], data = surdata3)
summary(cox.LOC143188)
## Call:
## coxph(formula = Surv(survival.years, vital_status) ~ RNA.counts.01[,
##     "LOC143188|143188"], data = surdata3)
```

```
##
##   n= 765, number of events= 118
##
##                                      coef exp(coef) se(coef)     z
## RNA.counts.01[, "LOC143188|143188"] 2.0934    8.1121   0.4118 5.083
##                                    Pr(>|z|)
## RNA.counts.01[, "LOC143188|143188"] 3.71e-07 ***
## ---
## Signif. codes:  0 '***' 0.001 '**' 0.01 '*' 0.05 '.' 0.1 ' ' 1
##
##                                    exp(coef) exp(-coef) lower .95
## RNA.counts.01[, "LOC143188|143188"]     8.112     0.1233     3.619
##                                    upper .95
## RNA.counts.01[, "LOC143188|143188"]     18.18
##
## Concordance= 0.528  (se = 0.032 )
## Likelihood ratio test= 12.63  on 1 df,   p=4e-04
## Wald test            = 25.84  on 1 df,   p=4e-07
## Score (logrank) test = 20.53  on 1 df,   p=6e-06
## Split at median expression of LOC143188
LOC143188.grps <- ifelse(RNA.counts.01[,"LOC143188|143188"] >
median(RNA.counts.01[,"LOC143188|143188"]), 1, 0)
table(LOC143188.grps)
## LOC143188.grps
##   0   1
## 383 382
km.LOC143188 <- survfit(Surv(survival.years, vital_status) ~ LOC143188.grps, data =
surdata3)
plot(km.LOC143188, col = 1:2, xlab = "Time (years)",  ylab = "Survival Probability",
     lwd = 2, main = paste("LOC143188 Expression and Survival"))
leg.txt <- c("Below median", "Above median")
legend("topright", leg.txt, lty = 1, col = 1:2, bty = "n", lwd = 2, title = "LOC143188
Expression")
```

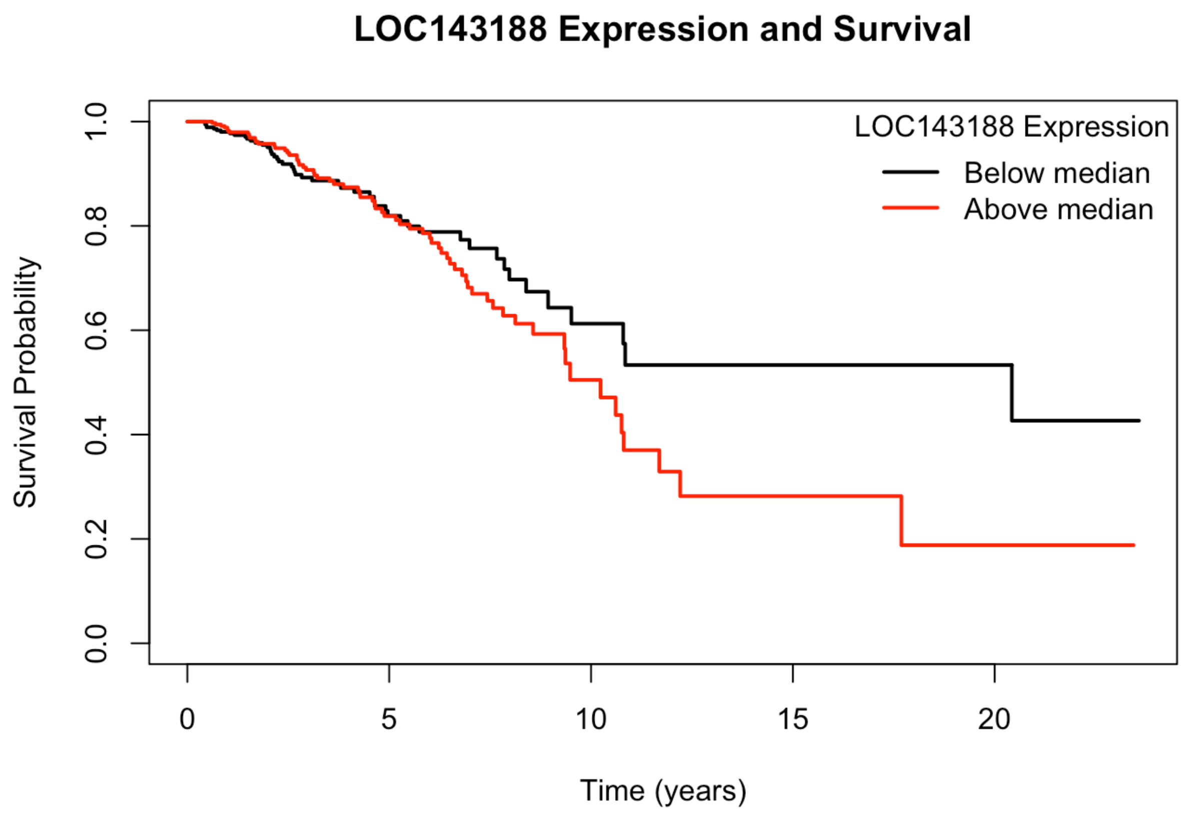


```
########################################### Check Broad results
############################
cox.PGK1 <- coxph(Surv(survival.years, vital_status) ~ RNA.counts.01[,"PGK1|5230"],
data = surdata3)
summary(cox.PGK1) # Logrank test p=0.007
## Call:
## coxph(formula = Surv(survival.years, vital_status) ~ RNA.counts.01[,
##     "PGK1|5230"], data = surdata3)
##
##   n= 765, number of events= 118
##
##                                coef exp(coef) se(coef)     z Pr(>|z|)
## RNA.counts.01[, "PGK1|5230"] 0.6589    1.9326   0.1336 4.933 8.08e-07 ***
## ---
## Signif. codes:  0 '***' 0.001 '**' 0.01 '*' 0.05 '.' 0.1 ' ' 1
##
##                              exp(coef) exp(-coef) lower .95 upper .95
## RNA.counts.01[, "PGK1|5230"]     1.933     0.5174     1.488     2.511
##
## Concordance= 0.681  (se = 0.032 )
## Likelihood ratio test= 23.55  on 1 df,   p=1e-06
## Wald test            = 24.34  on 1 df,   p=8e-07
## Score (logrank) test = 24.14  on 1 df,   p=9e-07
a <- RNA.counts.01[,"RASGEF1A|221002"]
b <- cut(a, quantile(a, c(0, 0.25, 0.5, 0.75, 1)), include.lowest = TRUE)
cox.PGK1 <- coxph(Surv(survival.years, vital_status) ~ b, data = surdata3)
summary(cox.PGK1) # Logrank test p=6e-06
## Call:
## coxph(formula = Surv(survival.years, vital_status) ~ b, data = surdata3)
##
##   n= 765, number of events= 118
```

```
##
##                    coef exp(coef) se(coef)      z Pr(>|z|)
## b(0.688,1.2] -0.9479    0.3875   0.2900 -3.269  0.00108 **
## b(1.2,1.86]  -0.5167    0.5965   0.2536 -2.038  0.04158 *
## b(1.86,4.97] -0.4605    0.6309   0.2391 -1.926  0.05406 .
## ---
## Signif. codes:  0 '***' 0.001 '**' 0.01 '*' 0.05 '.' 0.1 ' ' 1
##
##              exp(coef) exp(-coef) lower .95 upper .95
## b(0.688,1.2]    0.3875      2.580    0.2195    0.6842
## b(1.2,1.86]     0.5965      1.677    0.3629    0.9805
## b(1.86,4.97]    0.6309      1.585    0.3949    1.0081
##
## Concordance= 0.583  (se = 0.03 )
## Likelihood ratio test= 11.62  on 3 df,   p=0.009
## Wald test            = 11.5  on 3 df,   p=0.009
## Score (logrank) test = 11.99  on 3 df,   p=0.007
survdiff(Surv(survival.years, vital_status) ~ b, data = surdata3)
## Call:
## survdiff(formula = Surv(survival.years, vital_status) ~ b, data = surdata3)
##
##                     N Observed Expected (O-E)^2/E (O-E)^2/V
## b=[0.0587,0.688] 192       36     23.0  7.39e+00  9.320081
## b=(0.688,1.2]    191       18     29.4  4.40e+00  5.870440
## b=(1.2,1.86]     191       28     29.7  9.68e-02  0.129609
## b=(1.86,4.97]    191       36     36.0  4.74e-05  0.000069
##
##  Chisq= 12  on 3 degrees of freedom, p= 0.007
"PGK1|5230" %in% cox.top.unadjusted # TRUE
## [1] TRUE
"PGK1|5230" %in% cox.top # FALSE
## [1] FALSE
##################################Pseudogene data import
################################
load("~/Desktop/ICIBM_2019/annot.Rdata")
Pdat = readRDS("~/Desktop/ICIBM_2019/TCGA_BRCA.rds")
pseudogene_names_annot =
unique(annot$hgnc_symbol[grep("pseudogene",annot$gene_biotype)]);
pseudogene_names_annot = pseudogene_names_annot[!is.na(pseudogene_names_annot)]
# FIXED
pseudogene_names_annot = intersect(pseudogene_names_annot,row.names(Pdat))
Pdat = log2(Pdat[pseudogene_names_annot,substr(colnames(Pdat),14,15)=="01"]+1)
colnames(Pdat) = substr(colnames(Pdat),1,12)

## Common patients between pseudogene and clinical
common.patients <- base::intersect(colnames(Pdat), rownames(surdata2))

## Check the number of patients after filtering
nrow(surdata) # 1097
## [1] 1097
nrow(surdata2) # 1083
## [1] 1083
length(common.patients) # 1077
## [1] 1077
surdata3 <- surdata2[common.patients,]
dim(surdata3) # 1077  20 <------ Use surdata3
## [1] 1077   20
Pdat.01 = t(log2(Pdat[,common.patients]+1))
dim(Pdat.01) # 1077 7228
## [1] 1077 7228
############################################################################
## Kaplan-Meier Plot for Overall Survival
############################################################################
require(survival)
km <- survfit(Surv(survival.years, vital_status) ~ 1, data = surdata3)
```

```
plot(km, xlab = "Time (years)",  ylab = "Survival Probability",lwd = 2, main =
"Overall Survival")
```

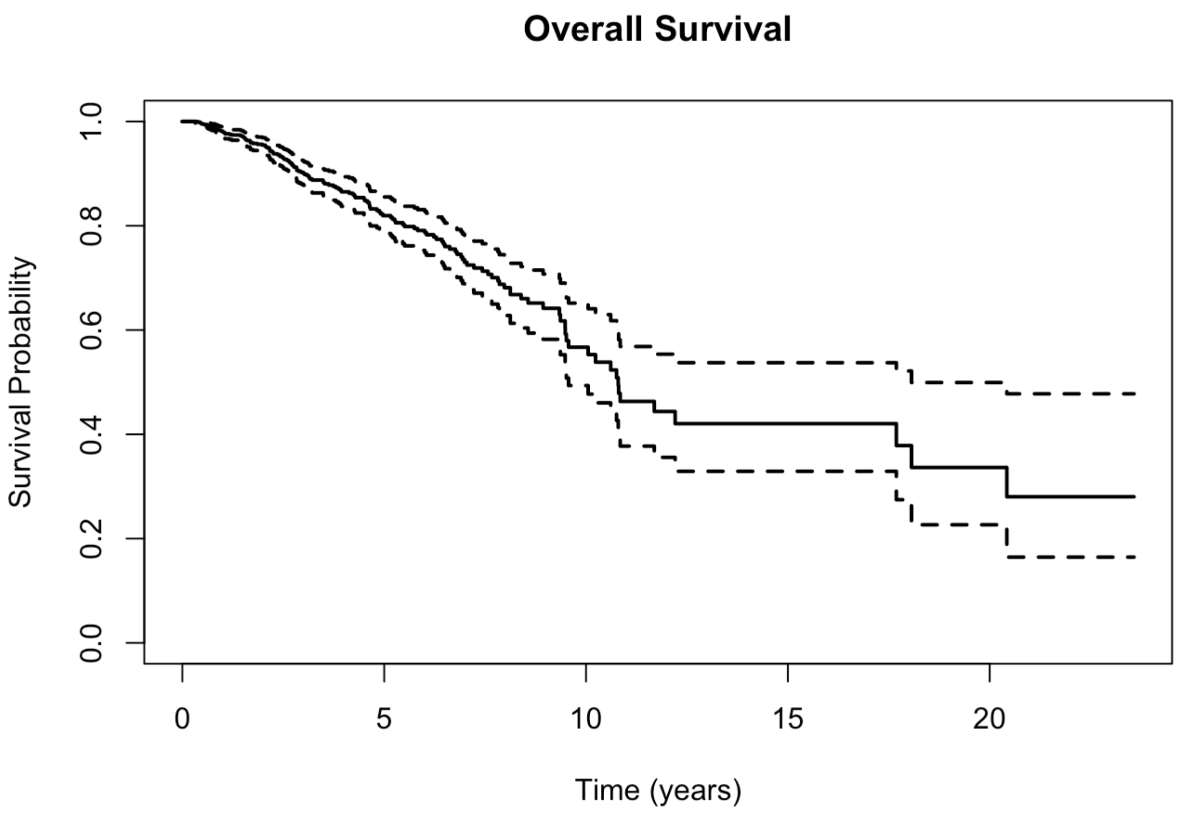


```
## double-check that rownames are equal
all.equal(rownames(Pdat.01), rownames(surdata3)) # TRUE
## [1] TRUE
length(rownames(Pdat.01)) # 1077
## [1] 1077
```

Multivariable model In order to create a multivariable model to assess multiple genes at once, we need to split the data into a training set and a test set. The model is built on the training data, and assessed using the test data, which enables us to avoid over-fitting. We then have a molecular signature which will tell us the prognosis of a patient based on a number of genes' expression levels.

```
## Split the data into 2/3 training and 1/3 test
## Fit model on 2/3 and evaluate on the test data

## X = expression data (patients in rows and genes in columns)
## S = Surv(survival.time, survival.status)

## GENE EXPRESSION DATA = RNA.counts1

## SURVIVAL
S <- Surv(surdata3$survival.years, surdata3$vital_status)

### training data and validation data
set.seed(1000) # set the seed to ensure reproducibility

## Traing set rows
## randomly sample 2/3 of subjects
train.idx <- sample(1:nrow(surdata3), round(nrow(surdata3)*2/3), replace = FALSE)
length(train.idx) # 718
## [1] 718
nrow(surdata3) # 1077
```

```
## [1] 1077
X.train <- Pdat.01[train.idx,]
S.train <- S[train.idx,]

## For test set rows use '-train.idx'
X.test <- Pdat.01[-train.idx,]
S.test <- S[-train.idx,]

## Check dimensions
dim(X.train) # 718 20532
## [1]  718 7228
dim(X.test) # 359 20532
## [1]  359 7228
```

We want to select the features for our model, which is the expression level of genes that are most associated with survival - using the filtering method.

```
## fitting univariate models
cox.uni.p <- rep(1,ncol(X.train))
m <- ncol(X.train)
for (i in 1:m)  {
  if (sum(X.train[,i])>=1){
    #print(i)
    cox0 <- coxph(S.train ~ X.train[,i])
    cox.uni.p[i] <- 1-pchisq(cox0$score, 1)
  }
}
names(cox.uni.p) <- colnames(X.train)
## Takes a minute but not bad
## YZ will double check Warning messages:
## 1: In fitter(X, Y, strats, offset, init, control, weights = weights,  ... :
##               Loglik converged before variable  1 ; beta may be infinite.

## histogram of univariate p-values
hist(cox.uni.p,main="",xlab="Univariate p-values")
```

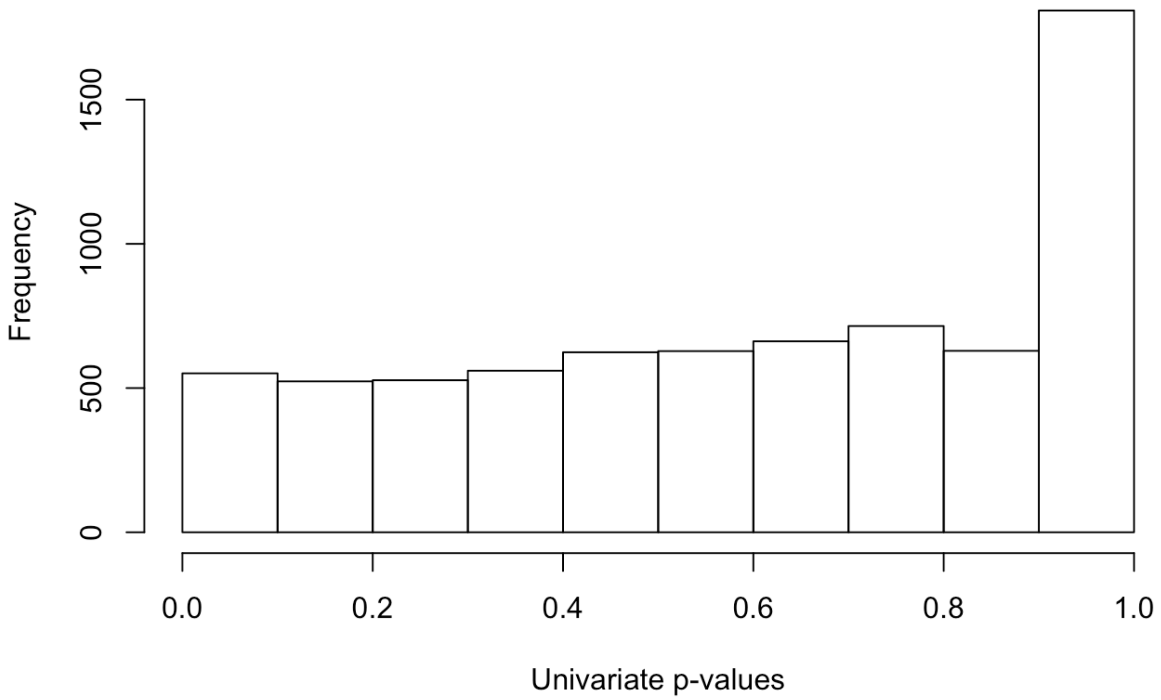


```
## Screen the top based on p-values, and required the adjusted p-value < 0.05
cox.uni.p <- sort(cox.uni.p,  decreasing=FALSE)  ## smallest first
sum(cox.uni.p < 0.05) # 994
## [1] 327
cox.top.unadjusted <- names(cox.uni.p)[cox.uni.p < 0.05]
adj.cox.uni.p <- p.adjust(cox.uni.p, method = "BH") # YZ: adjusted p-values
sum(adj.cox.uni.p < 0.05) # 7 -- Check how many significant
## [1] 14
(cox.top <- names(adj.cox.uni.p)[adj.cox.uni.p < 0.05]) # IDs of significant genes
##  [1] "HNRNPCP9" "OR4C7P"   "CTSLP8"   "VN1R6P"   "EEF1GP4"  "SUMO2P2"
##  [7] "BNIP3P23" "FAM60DP"  "MEMO1P3"  "PRSS3P4"  "HLA-K"    "CBX1P3"
## [13] "GVINP2"   "RPS10P20"
(cox.top.p.adj <- adj.cox.uni.p[adj.cox.uni.p < 0.05])  # adjusted p-values of
significant genes
##     HNRNPCP9       OR4C7P       CTSLP8       VN1R6P      EEF1GP4
## 6.291359e-10 7.702246e-06 4.257021e-05 6.700468e-04 8.691520e-04
##      SUMO2P2     BNIP3P23      FAM60DP      MEMO1P3      PRSS3P4
## 1.826371e-03 3.181924e-03 7.641224e-03 7.641224e-03 8.561481e-03
##        HLA-K       CBX1P3       GVINP2     RPS10P20
## 2.577825e-02 2.582926e-02 3.005881e-02 3.807989e-02
write.csv(cox.top.p.adj, file =
"~/Desktop/ICIBM_2019/SignificantPseudoFromUnivariateCoxModel.csv")
pseudo.cox.top.unadjusted = cox.top.unadjusted
####################################################################
## LASSO starting with top univariate genes
####################################################################

## penalized likelihood method using glmnet
set.seed(1000)

## Restrict starting to all significant genes based on univariate Cox model
```

```
## cv.glmnet does CV to determine the optimal lambda value
cv.lasso <- cv.glmnet(X.train[,cox.top], S.train, family = "cox", alpha=1)
plot(cv.lasso)
```

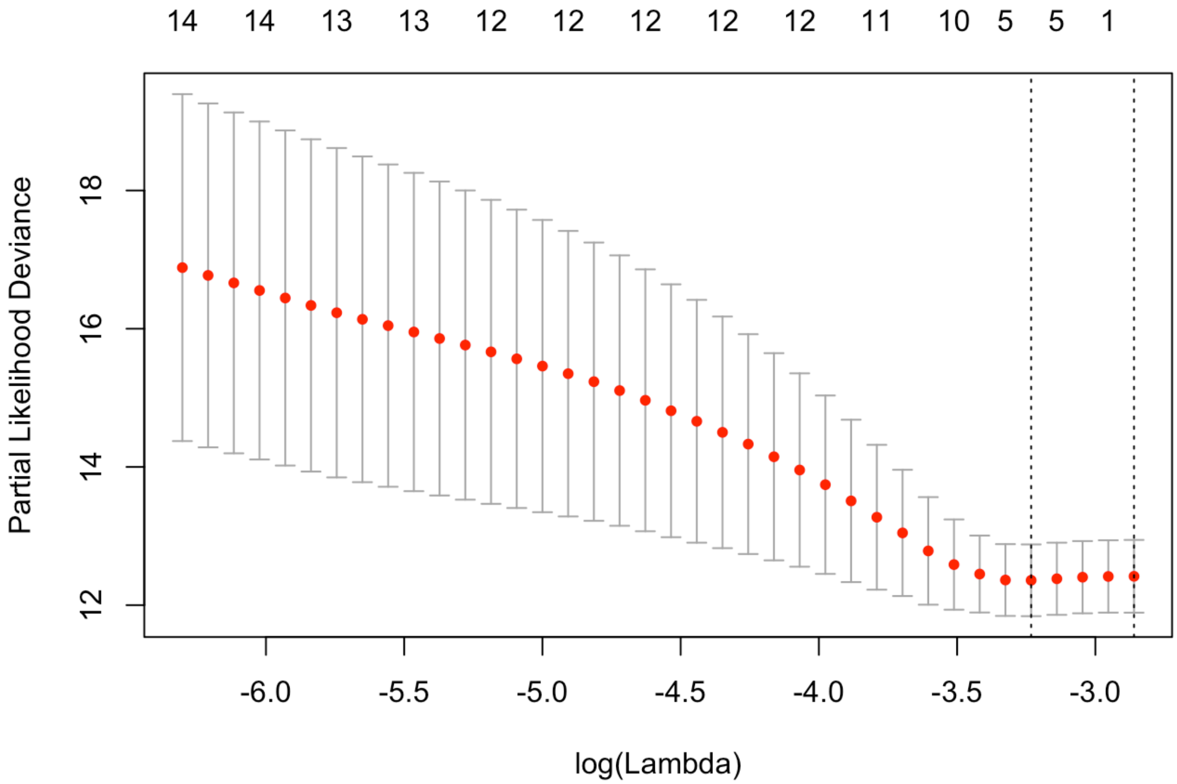


```
## How many predictors are selected at the optimal lambda value (lambda.min)?
coef.min <-  coef(cv.lasso, s = "lambda.min")
sum(coef.min != 0) # 3
## [1] 5
## lasso fit
coxfit.lasso <- glmnet(X.train[,cox.top], S.train, family = "cox", alpha=1)
plot(coxfit.lasso, xvar="lambda",label=TRUE)
abline(v=log(cv.lasso$lambda.min))
```

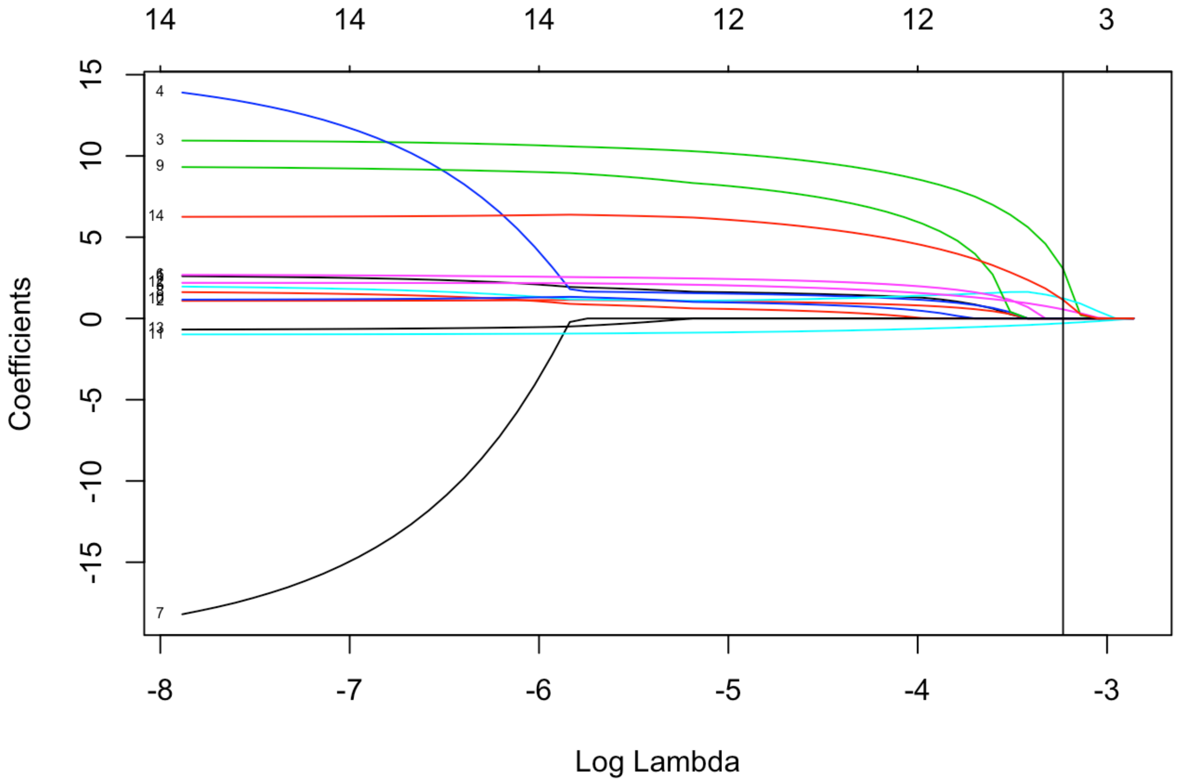


```
## genes selected with optimal model fitting
(coef.min <-  coef(cv.lasso, s = "lambda.min"))
## 14 x 1 sparse Matrix of class "dgCMatrix"
##                    1
## HNRNPCP9   .
## OR4C7P     .
## CTSLP8     3.0588287
## VN1R6P     .
## EEF1GP4    1.2414364
## SUMO2P2    .
## BNIP3P23   .
## FAM60DP    .
## MEMO1P3    .
## PRSS3P4    .
## HLA-K     -0.2952129
## CBX1P3     0.5596213
## GVINP2     .
## RPS10P20   1.1442176
(active.min <- which(as.matrix(coef.min != 0)))
## [1]  3  5 11 12 14
(index.min <- coef.min[active.min])
## [1]  3.0588287  1.2414364 -0.2952129  0.5596213  1.1442176
(sel.lasso <- rownames(coef.min)[active.min]) # Important
## [1] "CTSLP8"   "EEF1GP4"  "HLA-K"    "CBX1P3"   "RPS10P20"
#################################
## Test set validation - LASSO
#################################

## risk score
rs.lasso.train <- predict(cv.lasso, newx=X.train[,cox.top], s="lambda.min")
rs.lasso.test <- predict(cv.lasso, newx=X.test[,cox.top], s="lambda.min")
```

```
# ## median stratification
good.prog.lasso <- (rs.lasso.test < median(rs.lasso.train))
(fit.lasso <- survfit(S.test ~ good.prog.lasso))
## Call: survfit(formula = S.test ~ good.prog.lasso)
##
##                         n events median 0.95LCL 0.95UCL
## good.prog.lasso=FALSE 162     20   10.8    9.34      NA
## good.prog.lasso=TRUE  197     23   20.4    9.51      NA
# ## logrank test for validation set
# (logrank.lasso <- survdiff(S.test ~ good.prog.lasso))
(logrank.lasso <- survdiff(S.test ~ rs.lasso.test))
## Call:
## survdiff(formula = S.test ~ rs.lasso.test)
##
##                                    N Observed Expected (O-E)^2/E (O-E)^2/V
## rs.lasso.test=-0.573039701658486   1        0  0.35577  3.56e-01  3.63e-01
## rs.lasso.test=-0.556503671435327   1        0  0.55075  5.51e-01  5.72e-01
## rs.lasso.test=-0.555216391104874   1        0  0.05607  5.61e-02  5.63e-02
## rs.lasso.test=-0.52327573358892    1        0  0.20609  2.06e-01  2.08e-01
## rs.lasso.test=-0.50092516445794    1        0  0.23863  2.39e-01  2.41e-01
## rs.lasso.test=-0.494773091323389   1        0  0.01527  1.53e-02  1.53e-02
## rs.lasso.test=-0.493926508496288   1        0  0.06788  6.79e-02  6.82e-02
## rs.lasso.test=-0.486542489737652   1        0  0.04055  4.06e-02  4.07e-02
## rs.lasso.test=-0.483379587881337   1        0  0.02517  2.52e-02  2.52e-02
## rs.lasso.test=-0.480505464033639   1        0  0.09619  9.62e-02  9.67e-02
## rs.lasso.test=-0.475531495219321   1        0  0.11314  1.13e-01  1.14e-01
## rs.lasso.test=-0.467383321926992   1        0  0.08844  8.84e-02  8.89e-02
## rs.lasso.test=-0.460541212345434   1        1  0.03242  2.89e+01  2.90e+01
## rs.lasso.test=-0.458364954832669   1        0  0.35577  3.56e-01  3.63e-01
## rs.lasso.test=-0.455917019627899   1        0  0.12240  1.22e-01  1.23e-01
## rs.lasso.test=-0.454464408535339   1        0  0.02517  2.52e-02  2.52e-02
## rs.lasso.test=-0.447542764314065   1        0  0.16705  1.67e-01  1.68e-01
## rs.lasso.test=-0.444546394938532   1        0  0.05081  5.08e-02  5.11e-02
## rs.lasso.test=-0.440879812571304   1        0  0.02874  2.87e-02  2.88e-02
## rs.lasso.test=-0.438378380451008   1        0  0.31230  3.12e-01  3.18e-01
## rs.lasso.test=-0.436479926873024   1        0  0.08844  8.84e-02  8.89e-02
## rs.lasso.test=-0.435832993062095   1        0  0.04055  4.06e-02  4.07e-02
## rs.lasso.test=-0.432587322827643   1        0  0.03242  3.24e-02  3.25e-02
## rs.lasso.test=-0.425871819079074   2        0  0.13138  1.31e-01  1.32e-01
## rs.lasso.test=-0.425190010996266   1        0  0.04055  4.06e-02  4.07e-02
## rs.lasso.test=-0.419562244344757   1        0  0.01848  1.85e-02  1.85e-02
## rs.lasso.test=-0.41665922196144    1        0  0.23863  2.39e-01  2.41e-01
## rs.lasso.test=-0.415658812848873   1        1  0.00897  1.09e+02  1.10e+02
## rs.lasso.test=-0.415190008407567   1        0  0.08844  8.84e-02  8.89e-02
## rs.lasso.test=-0.410694199565869   1        0  0.04055  4.06e-02  4.07e-02
## rs.lasso.test=-0.409157647768273   1        0  0.00593  5.93e-03  5.95e-03
## rs.lasso.test=-0.407620874712374   1        1  0.01848  5.21e+01  5.23e+01
## rs.lasso.test=-0.404484056630443   1        0  0.15514  1.55e-01  1.56e-01
## rs.lasso.test=-0.401281385502131   1        1  0.22197  2.73e+00  2.75e+00
## rs.lasso.test=-0.396356781343206   1        0  0.02874  2.87e-02  2.88e-02
## rs.lasso.test=-0.395021252320514   1        0  0.04055  4.06e-02  4.07e-02
## rs.lasso.test=-0.393524108419035   1        0  0.02178  2.18e-02  2.18e-02
## rs.lasso.test=-0.390843276340406   1        0  0.02517  2.52e-02  2.52e-02
## rs.lasso.test=-0.389516280391522   1        0  0.20609  2.06e-01  2.08e-01
## rs.lasso.test=-0.382371219442064   1        0  0.15514  1.55e-01  1.56e-01
## rs.lasso.test=-0.380523807761067   1        0  0.04055  4.06e-02  4.07e-02
## rs.lasso.test=-0.378673263320635   1        0  0.25824  2.58e-01  2.61e-01
## rs.lasso.test=-0.377735119179011   1        0  0.09619  9.62e-02  9.67e-02
## rs.lasso.test=-0.37679139838113    1        1  0.47383  5.84e-01  6.03e-01
## rs.lasso.test=-0.37584204211279    1        0  0.06788  6.79e-02  6.82e-02
## rs.lasso.test=-0.374896569574627   1        1  0.04055  2.27e+01  2.28e+01
## rs.lasso.test=-0.37140002178719    1        0  0.03637  3.64e-02  3.65e-02
## rs.lasso.test=-0.370024151777944   1        0  0.02517  2.52e-02  2.52e-02
## rs.lasso.test=-0.369033623485926   2        1  0.01527  6.35e+01  6.37e+01
```

```
## rs.lasso.test=-0.36803695568134    1         0  0.02874  2.87e-02  2.88e-02
## rs.lasso.test=-0.365009435028821   1         0  0.08844  8.84e-02  8.89e-02
## rs.lasso.test=-0.363987523607648   2         1  0.09305  8.84e+00  8.90e+00
## rs.lasso.test=-0.362959124409413   1         0  0.00593  5.93e-03  5.95e-03
## rs.lasso.test=-0.362144135419367   1         0  0.01848  1.85e-02  1.85e-02
## rs.lasso.test=-0.36192416441244    1         0  0.03637  3.64e-02  3.65e-02
## rs.lasso.test=-0.359834264021335   3         1  0.72420  1.05e-01  1.10e-01
## rs.lasso.test=-0.355572386852237   1         0  0.03637  3.64e-02  3.65e-02
## rs.lasso.test=-0.353399132625847   2         0  0.66390  6.64e-01  6.87e-01
## rs.lasso.test=-0.349995614420077   1         0  0.25824  2.58e-01  2.61e-01
## rs.lasso.test=-0.347110955618751   1         0  0.25824  2.58e-01  2.61e-01
## rs.lasso.test=-0.346701983376261   1         0  0.19220  1.92e-01  1.94e-01
## rs.lasso.test=-0.345558984617359   2         1  0.19164  3.41e+00  3.44e+00
## rs.lasso.test=-0.343249166637824   1         0  0.02874  2.87e-02  2.88e-02
## rs.lasso.test=-0.34090693345582    4         1  0.43809  7.21e-01  7.32e-01
## rs.lasso.test=-0.338531475074181   1         0  0.00000       NaN       NaN
## rs.lasso.test=-0.33455606417855    1         0  0.04055  4.06e-02  4.07e-02
## rs.lasso.test=-0.332026207141044   1         1  0.20609  3.06e+00  3.08e+00
## rs.lasso.test=-0.331197202867518   2         1  0.36104  1.13e+00  1.15e+00
## rs.lasso.test=-0.329943327208801   1         0  0.25824  2.58e-01  2.61e-01
## rs.lasso.test=-0.328680139162713   1         0  0.25824  2.58e-01  2.61e-01
## rs.lasso.test=-0.328521810065612   1         0  0.03637  3.64e-02  3.65e-02
## rs.lasso.test=-0.32836354149611    1         0  0.00000       NaN       NaN
## rs.lasso.test=-0.327407516308771   3         0  0.10321  1.03e-01  1.04e-01
## rs.lasso.test=-0.326125333919278   2         1  0.39369  9.34e-01  9.50e-01
## rs.lasso.test=-0.324331320593922   1         0  0.08140  8.14e-02  8.18e-02
## rs.lasso.test=-0.323544467552721   1         0  0.10452  1.05e-01  1.05e-01
## rs.lasso.test=-0.323531779744518   1         0  0.86186  8.62e-01  9.41e-01
## rs.lasso.test=-0.322546796722853   1         0  0.25824  2.58e-01  2.61e-01
## rs.lasso.test=-0.320898430472181   1         0  0.06182  6.18e-02  6.21e-02
## rs.lasso.test=-0.318880339463611   1         0  0.00000       NaN       NaN
## rs.lasso.test=-0.318224199203019   1         0  0.03637  3.64e-02  3.65e-02
## rs.lasso.test=-0.316871401515824   1         0  0.01848  1.85e-02  1.85e-02
## rs.lasso.test=-0.315355750391469   1         0  0.04055  4.06e-02  4.07e-02
## rs.lasso.test=-0.314133715054142   2         0  0.12195  1.22e-01  1.23e-01
## rs.lasso.test=-0.31274852678362    1         0  0.12240  1.22e-01  1.23e-01
## rs.lasso.test=-0.31135223714443    2         1  0.15370  4.66e+00  4.69e+00
## rs.lasso.test=-0.309944688752979   1         0  0.23863  2.39e-01  2.41e-01
## rs.lasso.test=-0.308525721025923   1         0  0.15514  1.55e-01  1.56e-01
## rs.lasso.test=-0.305652868720293   2         0  0.14556  1.46e-01  1.47e-01
## rs.lasso.test=-0.302732328266523   1         0  0.01209  1.21e-02  1.21e-02
## rs.lasso.test=-0.300575763432529   1         0  0.06788  6.79e-02  6.82e-02
## rs.lasso.test=-0.299762690769809   2         0  0.32127  3.21e-01  3.27e-01
## rs.lasso.test=-0.299556187022124   1         0  0.31230  3.12e-01  3.18e-01
## rs.lasso.test=-0.298726318246479   1         0  0.02517  2.52e-02  2.52e-02
## rs.lasso.test=-0.298259004011559   1         0  0.02178  2.18e-02  2.18e-02
## rs.lasso.test=-0.296742487249454   1         0  0.16705  1.67e-01  1.68e-01
## rs.lasso.test=-0.296227759148057   1         0  0.01209  1.21e-02  1.21e-02
## rs.lasso.test=-0.295285660325704   1         0  0.06788  6.79e-02  6.82e-02
## rs.lasso.test=-0.295212946919721   2         0  0.08699  8.70e-02  8.75e-02
## rs.lasso.test=-0.292114000237302   2         0  0.86796  8.68e-01  9.08e-01
## rs.lasso.test=-0.289120664345145   1         0  0.12240  1.22e-01  1.23e-01
## rs.lasso.test=-0.2876715535204     1         0  0.13304  1.33e-01  1.34e-01
## rs.lasso.test=-0.284124334858782   2         0  0.05264  5.26e-02  5.29e-02
## rs.lasso.test=-0.282483109012264   1         0  0.01848  1.85e-02  1.85e-02
## rs.lasso.test=-0.280826913870952   4         0  0.41556  4.16e-01  4.21e-01
## rs.lasso.test=-0.280125130177501   2         1  0.12899  5.88e+00  5.92e+00
## rs.lasso.test=-0.278589680615426   1         1  0.10452  7.67e+00  7.72e+00
## rs.lasso.test=-0.278229664631834   1         0  0.22197  2.22e-01  2.24e-01
## rs.lasso.test=-0.277468644819747   1         1  0.03637  2.55e+01  2.56e+01
## rs.lasso.test=-0.276193249362555   1         0  0.00000       NaN       NaN
## rs.lasso.test=-0.275766071339726   1         0  0.08844  8.84e-02  8.89e-02
## rs.lasso.test=-0.274047529080454   1         0  0.05607  5.61e-02  5.63e-02
## rs.lasso.test=-0.273761348081554   1         0  0.31230  3.12e-01  3.18e-01
## rs.lasso.test=-0.27107020738436 3   1         0  0.04055  4.06e-02  4.07e-02
```

```
## rs.lasso.test=-0.270561473129949   1         0  0.04055  4.06e-02  4.07e-02
## rs.lasso.test=-0.268793410949222   1         0  0.31230  3.12e-01  3.18e-01
## rs.lasso.test=-0.268509438128029   1         0  0.04055  4.06e-02  4.07e-02
## rs.lasso.test=-0.267692400966011   1         0  0.15514  1.55e-01  1.56e-01
## rs.lasso.test=-0.267642137011109   1         0  0.04055  4.06e-02  4.07e-02
## rs.lasso.test=-0.267008282610611   4         1  0.38477  9.84e-01  9.97e-01
## rs.lasso.test=-0.266914488306689   1         0  0.05607  5.61e-02  5.63e-02
## rs.lasso.test=-0.265446593669637   1         0  0.08844  8.84e-02  8.89e-02
## rs.lasso.test=-0.265205797014178   1         0  0.03242  3.24e-02  3.25e-02
## rs.lasso.test=-0.263385655930469   2         0  0.22302  2.23e-01  2.25e-01
## rs.lasso.test=-0.263257157798456   1         0  0.01209  1.21e-02  1.21e-02
## rs.lasso.test=-0.261547553772031   1         0  0.00000       NaN       NaN
## rs.lasso.test=-0.260737325380419   1         0  0.03637  3.64e-02  3.65e-02
## rs.lasso.test=-0.259691177355848   1         0  0.05607  5.61e-02  5.63e-02
## rs.lasso.test=-0.258075246495044   1         0  0.03242  3.24e-02  3.25e-02
## rs.lasso.test=-0.257916109312289   1         0  0.04055  4.06e-02  4.07e-02
## rs.lasso.test=-0.255244961445562   1         0  0.02517  2.52e-02  2.52e-02
## rs.lasso.test=-0.254009151538379   2         0  0.08111  8.11e-02  8.17e-02
## rs.lasso.test=-0.252076385489371   1         1  0.05081  1.77e+01  1.78e+01
## rs.lasso.test=-0.251505956031338   1         0  0.05607  5.61e-02  5.63e-02
## rs.lasso.test=-0.25077519808198    1         0  0.02178  2.18e-02  2.18e-02
## rs.lasso.test=-0.25012365632714    2         0  0.05264  5.26e-02  5.29e-02
## rs.lasso.test=-0.249279855116387   1         0  1.19520  1.20e+00  1.41e+00
## rs.lasso.test=-0.249232436431545   1         1  0.13304  5.65e+00  5.69e+00
## rs.lasso.test=-0.24867131533142    1         0  0.00593  5.93e-03  5.95e-03
## rs.lasso.test=-0.248150599740246   1         0  0.02874  2.87e-02  2.88e-02
## rs.lasso.test=-0.246292012718576   1         0  0.04055  4.06e-02  4.07e-02
## rs.lasso.test=-0.246156841864897   2         1  0.03110  3.02e+01  3.03e+01
## rs.lasso.test=-0.244141998956925   1         0  0.00000       NaN       NaN
## rs.lasso.test=-0.242105677049791   4         0  0.54103  5.41e-01  5.54e-01
## rs.lasso.test=-0.240751907531795   1         0  0.25824  2.58e-01  2.61e-01
## rs.lasso.test=-0.240093710801636   1         0  0.01848  1.85e-02  1.85e-02
## rs.lasso.test=-0.240047471597882   1         0  0.05081  5.08e-02  5.11e-02
## rs.lasso.test=-0.239686586021041   1         0  0.06182  6.18e-02  6.21e-02
## rs.lasso.test=-0.235863736732732   2         0  0.30535  3.05e-01  3.09e-01
## rs.lasso.test=-0.233737341901346   1         0  0.03637  3.64e-02  3.65e-02
## rs.lasso.test=-0.231587331859823   1         0  0.12240  1.22e-01  1.23e-01
## rs.lasso.test=-0.229413243246553   1         0  0.06182  6.18e-02  6.21e-02
## rs.lasso.test=-0.227900278216102   1         0  0.31230  3.12e-01  3.18e-01
## rs.lasso.test=-0.227214599626171   1         1  1.19520  3.19e-02  3.77e-02
## rs.lasso.test=-0.225908122789793   1         0  0.00295  2.95e-03  2.96e-03
## rs.lasso.test=-0.222741673330045   1         0  0.25824  2.58e-01  2.61e-01
## rs.lasso.test=-0.222394880956144   1         0  0.03637  3.64e-02  3.65e-02
## rs.lasso.test=-0.220466367950055   2         0  0.07455  7.46e-02  7.50e-02
## rs.lasso.test=-0.218299789841005   1         0  0.20609  2.06e-01  2.08e-01
## rs.lasso.test=-0.218164461071113   1         0  0.00000       NaN       NaN
## rs.lasso.test=-0.216577938506998   1         0  0.08844  8.84e-02  8.89e-02
## rs.lasso.test=-0.215835403171606   2         0  0.15026  1.50e-01  1.52e-01
## rs.lasso.test=-0.213478628395332   2         0  0.16060  1.61e-01  1.62e-01
## rs.lasso.test=-0.212935181399591   1         0  0.31230  3.12e-01  3.18e-01
## rs.lasso.test=-0.208679579261377   2         1  0.02812  3.36e+01  3.37e+01
## rs.lasso.test=-0.207365731125389   1         0  0.04055  4.06e-02  4.07e-02
## rs.lasso.test=-0.206236085628346   1         0  0.04055  4.06e-02  4.07e-02
## rs.lasso.test=-0.203762435131826   1         0  0.20609  2.06e-01  2.08e-01
## rs.lasso.test=-0.202897414575109   1         0  0.00295  2.95e-03  2.96e-03
## rs.lasso.test=-0.201257970040637   2         0  0.28839  2.88e-01  2.92e-01
## rs.lasso.test=-0.198515183153665   1         0  0.05607  5.61e-02  5.63e-02
## rs.lasso.test=-0.196153861004462   2         0  0.01491  1.49e-02  1.50e-02
## rs.lasso.test=-0.193552794851825   3         0  0.08111  8.11e-02  8.17e-02
## rs.lasso.test=-0.190918067850911   1         0  0.03242  3.24e-02  3.25e-02
## rs.lasso.test=-0.190754252178355   1         0  0.02517  2.52e-02  2.52e-02
## rs.lasso.test=-0.189153065642723   1         0  0.04055  4.06e-02  4.07e-02
## rs.lasso.test=-0.188248910004723   1         0  0.08844  8.84e-02  8.89e-02
## rs.lasso.test=-0.187950889852539   1         0  0.03637  3.64e-02  3.65e-02
## rs.lasso.test=-0.185555292963742   1         1  0.86186  2.21e-02  2.42e-02
```

```
## rs.lasso.test=-0.184979731721417   1         0  0.08844  8.84e-02  8.89e-02
## rs.lasso.test=-0.184898216077565   1         0  0.08140  8.14e-02  8.18e-02
## rs.lasso.test=-0.183151840718585   1         0  0.00000       NaN       NaN
## rs.lasso.test=-0.182804093763327   1         0  0.06182  6.18e-02  6.21e-02
## rs.lasso.test=-0.18002676398933    1         0  0.00897  8.97e-03  9.00e-03
## rs.lasso.test=-0.178962768715674   1         0  0.25824  2.58e-01  2.61e-01
## rs.lasso.test=-0.177211658230288   2         0  0.16407  1.64e-01  1.66e-01
## rs.lasso.test=-0.177087457159456   1         0  0.12240  1.22e-01  1.23e-01
## rs.lasso.test=-0.176668540778664   1         0  0.11314  1.13e-01  1.14e-01
## rs.lasso.test=-0.174347109200533   1         0  0.66186  6.62e-01  6.96e-01
## rs.lasso.test=-0.174131389047507   1         0  0.16705  1.67e-01  1.68e-01
## rs.lasso.test=-0.172291198799167   1         0  0.02517  2.52e-02  2.52e-02
## rs.lasso.test=-0.171877444839756   1         0  0.12240  1.22e-01  1.23e-01
## rs.lasso.test=-0.170942336486921   1         0  0.03242  3.24e-02  3.25e-02
## rs.lasso.test=-0.169040879557591   1         0  0.05607  5.61e-02  5.63e-02
## rs.lasso.test=-0.16853044064267    1         0  0.47383  4.74e-01  4.89e-01
## rs.lasso.test=-0.166866982600332   1         0  0.03637  3.64e-02  3.65e-02
## rs.lasso.test=-0.163477545830352   1         0  0.20609  2.06e-01  2.08e-01
## rs.lasso.test=-0.163245669989954   1         0  0.09619  9.62e-02  9.67e-02
## rs.lasso.test=-0.162536556909636   1         0  0.03637  3.64e-02  3.65e-02
## rs.lasso.test=-0.159474560675998   2         0  0.04026  4.03e-02  4.05e-02
## rs.lasso.test=-0.159135818790718   1         0  0.03242  3.24e-02  3.25e-02
## rs.lasso.test=-0.158229104730499   1         0  0.25824  2.58e-01  2.61e-01
## rs.lasso.test=-0.156820221202896   1         0  0.04055  4.06e-02  4.07e-02
## rs.lasso.test=-0.156367713447068   1         0  0.00593  5.93e-03  5.95e-03
## rs.lasso.test=-0.154499025446537   1         0  0.47383  4.74e-01  4.89e-01
## rs.lasso.test=-0.153214852761606   1         0  0.22197  2.22e-01  2.24e-01
## rs.lasso.test=-0.151950789780722   1         0  0.09619  9.62e-02  9.67e-02
## rs.lasso.test=-0.151140802235871   1         0  0.19220  1.92e-01  1.94e-01
## rs.lasso.test=-0.150807495509758   1         0  0.25824  2.58e-01  2.61e-01
## rs.lasso.test=-0.148731161735139   1         1  0.55075  3.66e-01  3.81e-01
## rs.lasso.test=-0.147610673865583   1         0  0.03242  3.24e-02  3.25e-02
## rs.lasso.test=-0.146766221404007   1         1  0.06788  1.28e+01  1.29e+01
## rs.lasso.test=-0.146480319013169   1         0  0.04055  4.06e-02  4.07e-02
## rs.lasso.test=-0.143885992517152   1         0  0.00000       NaN       NaN
## rs.lasso.test=-0.143811161166982   1         1  0.08140  1.04e+01  1.04e+01
## rs.lasso.test=-0.143467899461781   2         0  0.12396  1.24e-01  1.25e-01
## rs.lasso.test=-0.143102320320947   1         0  0.06788  6.79e-02  6.82e-02
## rs.lasso.test=-0.141106777093494   1         0  0.02178  2.18e-02  2.18e-02
## rs.lasso.test=-0.140467593320032   1         0  0.11314  1.13e-01  1.14e-01
## rs.lasso.test=-0.140118456820392   2         0  0.19220  1.92e-01  1.94e-01
## rs.lasso.test=-0.137008818366843   1         1  0.06182  1.42e+01  1.43e+01
## rs.lasso.test=-0.136716490543072   3         0  0.17730  1.77e-01  1.79e-01
## rs.lasso.test=-0.136138629986289   1         0  0.12240  1.22e-01  1.23e-01
## rs.lasso.test=-0.134795274503196   1         0  0.11314  1.13e-01  1.14e-01
## rs.lasso.test=-0.131292482660103   1         0  0.06788  6.79e-02  6.82e-02
## rs.lasso.test=-0.130190748870352   1         0  0.03242  3.24e-02  3.25e-02
## rs.lasso.test=-0.129749093964887   1         0  0.04055  4.06e-02  4.07e-02
## rs.lasso.test=-0.126446709874246   1         0  0.00295  2.95e-03  2.96e-03
## rs.lasso.test=-0.124092691804929   1         0  0.04055  4.06e-02  4.07e-02
## rs.lasso.test=-0.123929050686733   1         0  0.31230  3.12e-01  3.18e-01
## rs.lasso.test=-0.123907393418927   1         0  0.00000       NaN       NaN
## rs.lasso.test=-0.122553318563459   2         1  0.13326  5.64e+00  5.67e+00
## rs.lasso.test=-0.121117074270945   1         0  0.00000       NaN       NaN
## rs.lasso.test=-0.118865634669113   1         1  0.35577  1.17e+00  1.19e+00
## rs.lasso.test=-0.111929964609327   1         0  0.55075  5.51e-01  5.72e-01
## rs.lasso.test=-0.111301745972121   2         0  0.06788  6.79e-02  6.82e-02
## rs.lasso.test=-0.110061276608796   1         0  0.12240  1.22e-01  1.23e-01
## rs.lasso.test=-0.107421737896597   1         1  0.31230  1.51e+00  1.54e+00
## rs.lasso.test=-0.105911887684704   1         0  0.02517  2.52e-02  2.52e-02
## rs.lasso.test=-0.105729596142554   1         0  0.04055  4.06e-02  4.07e-02
## rs.lasso.test=-0.104832367413141   1         1  0.09619  8.49e+00  8.54e+00
## rs.lasso.test=-0.0953648447053152  2         0  0.35285  3.53e-01  3.59e-01
## rs.lasso.test=-0.0897828275108606  1         0  0.05081  5.08e-02  5.11e-02
## rs.lasso.test=-0.0895879923566933  1         0  0.31230  3.12e-01  3.18e-01
```

```
## rs.lasso.test=-0.0885491083806218  1        0  0.20609  2.06e-01  2.08e-01
## rs.lasso.test=-0.0858842705176928  1        0  0.35577  3.56e-01  3.63e-01
## rs.lasso.test=-0.0842801640998094  1        1  0.05081  1.77e+01  1.78e+01
## rs.lasso.test=-0.0818939993538045  1        1  0.23863  2.43e+00  2.45e+00
## rs.lasso.test=-0.0793014832636207  1        0  0.04055  4.06e-02  4.07e-02
## rs.lasso.test=-0.0792986194340084  1        0  0.23863  2.39e-01  2.41e-01
## rs.lasso.test=-0.078845024047849   1        0  0.25824  2.58e-01  2.61e-01
## rs.lasso.test=-0.078230655301941   1        0  0.03637  3.64e-02  3.65e-02
## rs.lasso.test=-0.0780065568428514  1        0  0.00000       NaN       NaN
## rs.lasso.test=-0.0760477395570614  1        0  0.23863  2.39e-01  2.41e-01
## rs.lasso.test=-0.0748303028837278  1        0  0.35577  3.56e-01  3.63e-01
## rs.lasso.test=-0.0743247105246957  1        0  0.12240  1.22e-01  1.23e-01
## rs.lasso.test=-0.0706779820617448  1        0  0.00000       NaN       NaN
## rs.lasso.test=-0.0690561782382691  1        0  0.02517  2.52e-02  2.52e-02
## rs.lasso.test=-0.0689130086601495  1        0  0.03242  3.24e-02  3.25e-02
## rs.lasso.test=-0.0686749381834987  1        1  0.41133  8.42e-01  8.65e-01
## rs.lasso.test=-0.0644950658891775  1        1  0.25824  2.13e+00  2.15e+00
## rs.lasso.test=-0.0615374475740059  1        0  0.07459  7.46e-02  7.50e-02
## rs.lasso.test=-0.061522093966884   1        0  0.04055  4.06e-02  4.07e-02
## rs.lasso.test=-0.0590359305085     1        0  0.04055  4.06e-02  4.07e-02
## rs.lasso.test=-0.054871529934019   1        1  0.05607  1.59e+01  1.60e+01
## rs.lasso.test=-0.0536173063032456  1        0  0.04055  4.06e-02  4.07e-02
## rs.lasso.test=-0.048856564093652   1        0  0.66186  6.62e-01  6.96e-01
## rs.lasso.test=-0.0447149471155024  1        0  0.06788  6.79e-02  6.82e-02
## rs.lasso.test=-0.041315269094273   1        0  0.31230  3.12e-01  3.18e-01
## rs.lasso.test=-0.0396667533803059  1        0  0.03242  3.24e-02  3.25e-02
## rs.lasso.test=-0.0386410620209016  1        1  0.14403  5.09e+00  5.12e+00
## rs.lasso.test=-0.0369548766373633  1        0  0.02874  2.87e-02  2.88e-02
## rs.lasso.test=-0.0337929064641999  1        0  0.12240  1.22e-01  1.23e-01
## rs.lasso.test=-0.0234419225924188  1        0  0.00000       NaN       NaN
## rs.lasso.test=-0.0226640417755382  1        0  0.06788  6.79e-02  6.82e-02
## rs.lasso.test=-0.0195135420281172  1        0  0.23863  2.39e-01  2.41e-01
## rs.lasso.test=-0.0184463516771626  1        0  0.12240  1.22e-01  1.23e-01
## rs.lasso.test=-0.0177857285148555  1        0  0.12240  1.22e-01  1.23e-01
## rs.lasso.test=-0.0164582910158544  1        0  0.01848  1.85e-02  1.85e-02
## rs.lasso.test=-0.0141390148375133  1        0  0.25824  2.58e-01  2.61e-01
## rs.lasso.test=-0.00799060244170041 1        1  0.19220  3.39e+00  3.42e+00
## rs.lasso.test=0.00355793699927923  1        1  0.16705  4.15e+00  4.19e+00
## rs.lasso.test=0.0048667876127769   1        1  0.02178  4.39e+01  4.41e+01
## rs.lasso.test=0.00510395564949259  1        0  0.04055  4.06e-02  4.07e-02
## rs.lasso.test=0.00697044386003459  1        0  0.02517  2.52e-02  2.52e-02
## rs.lasso.test=0.0117920693280938   1        0  0.05607  5.61e-02  5.63e-02
## rs.lasso.test=0.0219805797613409   1        0  0.05607  5.61e-02  5.63e-02
## rs.lasso.test=0.0247220588729118   1        0  0.04055  4.06e-02  4.07e-02
## rs.lasso.test=0.0297522240387684   1        0  0.03242  3.24e-02  3.25e-02
## rs.lasso.test=0.0333730288404643   1        0  0.04055  4.06e-02  4.07e-02
## rs.lasso.test=0.0399107591156489   1        0  0.13304  1.33e-01  1.34e-01
## rs.lasso.test=0.0401634879180114   1        0  0.04055  4.06e-02  4.07e-02
## rs.lasso.test=0.0523338422943921   1        0  1.19520  1.20e+00  1.41e+00
## rs.lasso.test=0.0591746832864844   1        0  0.03242  3.24e-02  3.25e-02
## rs.lasso.test=0.0654609372252478   1        0  0.03637  3.64e-02  3.65e-02
## rs.lasso.test=0.0672204516513513   1        0  0.00000       NaN       NaN
## rs.lasso.test=0.076362919726225l   1        0  0.02874  2.87e-02  2.88e-02
## rs.lasso.test=0.0884703765933298   1        0  0.31230  3.12e-01  3.18e-01
## rs.lasso.test=0.0946342691038461   1        0  0.03242  3.24e-02  3.25e-02
## rs.lasso.test=0.106717300741457    1        1  0.01209  8.07e+01  8.10e+01
## rs.lasso.test=0.134128352232123    1        0  0.03242  3.24e-02  3.25e-02
## rs.lasso.test=0.161634185215968    1        0  0.00897  8.97e-03  9.00e-03
## rs.lasso.test=0.188020343695311    1        0  0.05607  5.61e-02  5.63e-02
## rs.lasso.test=0.249638114492578    1        0  0.05607  5.61e-02  5.63e-02
## rs.lasso.test=0.425095509586208    1        0  0.08844  8.84e-02  8.89e-02
## rs.lasso.test=0.536880942214881    1        0  0.03242  3.24e-02  3.25e-02
##
##  Chisq= 723  on 290 degrees of freedom, p= <2e-16
(p.median.lasso <- 1-pchisq(logrank.lasso$chisq, 1))
```

```
## [1] 0
## cox regression on risk score
#cox.lasso <- coxph(S.test ~ good.prog.lasso)
cox.lasso <- coxph(S.test ~ rs.lasso.test)
summary(cox.lasso)
## Call:
## coxph(formula = S.test ~ rs.lasso.test)
##
##   n= 359, number of events= 43
##
##                  coef exp(coef) se(coef)     z Pr(>|z|)
## rs.lasso.test 0.2955    1.3438   1.0509 0.281    0.779
##
##               exp(coef) exp(-coef) lower .95 upper .95
## rs.lasso.test     1.344     0.7442    0.1713     10.54
##
## Concordance= 0.488  (se = 0.062 )
## Likelihood ratio test= 0.08  on 1 df,   p=0.8
## Wald test            = 0.08  on 1 df,   p=0.8
## Score (logrank) test = 0.08  on 1 df,   p=0.8
## C-index
## C-index assessment - for two selected individuals, the probability that the one
with the higher score (shorter survival) will actually have the shorter survival time.
Good biomarkers have indexes between 0.7 - 0.8.
#(c.lasso <- rcorrcens(S.test ~ good.prog.lasso)[,"C"])
(c.lasso <- rcorrcens(S.test ~ rs.lasso.test)[,"C"]) # 0.4100251
## [1] 0.5115873
## kaplan-meier curves
plot(fit.lasso, lwd = 2, lty = c(1,1), col = c("red","blue"), xlab = 'Time (years)',
     ylab = 'Estimated Survival Function')
legend("topright", legend=c('high risk', 'low risk'), lty = c(1,1),
       col = c("red", "blue"), lwd = 2, bty = "n")
text(6,0.9,paste("p =",round(p.median.lasso,3)))
text(6,0.85,paste("C =",round(c.lasso,3)))
title("Kaplan-Meier Curves, Lasso w/screen")
```

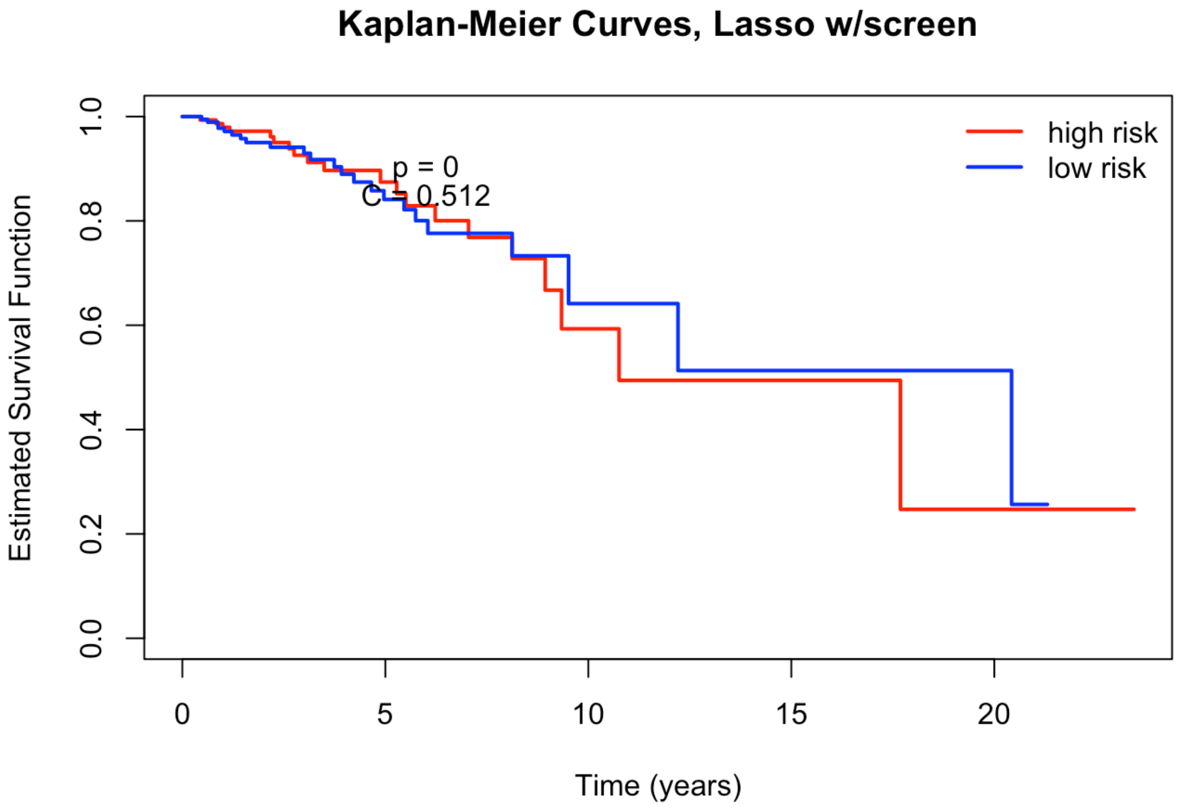


```
########################################################
## YZ newly added code for ctree - YZ 20180628
########################################################

#install.packages("partykit")
#install.packages("coin")
library("partykit")
library("coin")

##Convert all data frame character columns to factors
temp <- surdata3
temp$pathologic_stage <- substring(surdata3$pathologic_stage, 7)
surdata4 <- as.data.frame(unclass(temp[, -1]))
rm(temp)

# BRCA_ctree1 <- ctree(Surv(survival.years, vital_status) ~ pathologic_stage +
pathology_T_stage + pathology_N_stage + pathology_M_stage
#                       + gender + radiation_therapy + histological_type +
number_of_lymph_nodes
#                       + race + ethnicity, data = surdata4)
# plot(BRCA_ctree1)

## Consider expression only
BRCA_ctree2 <- ctree(Surv(survival.years, vital_status) ~ .,
                     data = data.frame(survival.years=surdata4$survival.years,
vital_status=surdata4$vital_status, Pdat.01[, sel.lasso]))
plot(BRCA_ctree2)
```

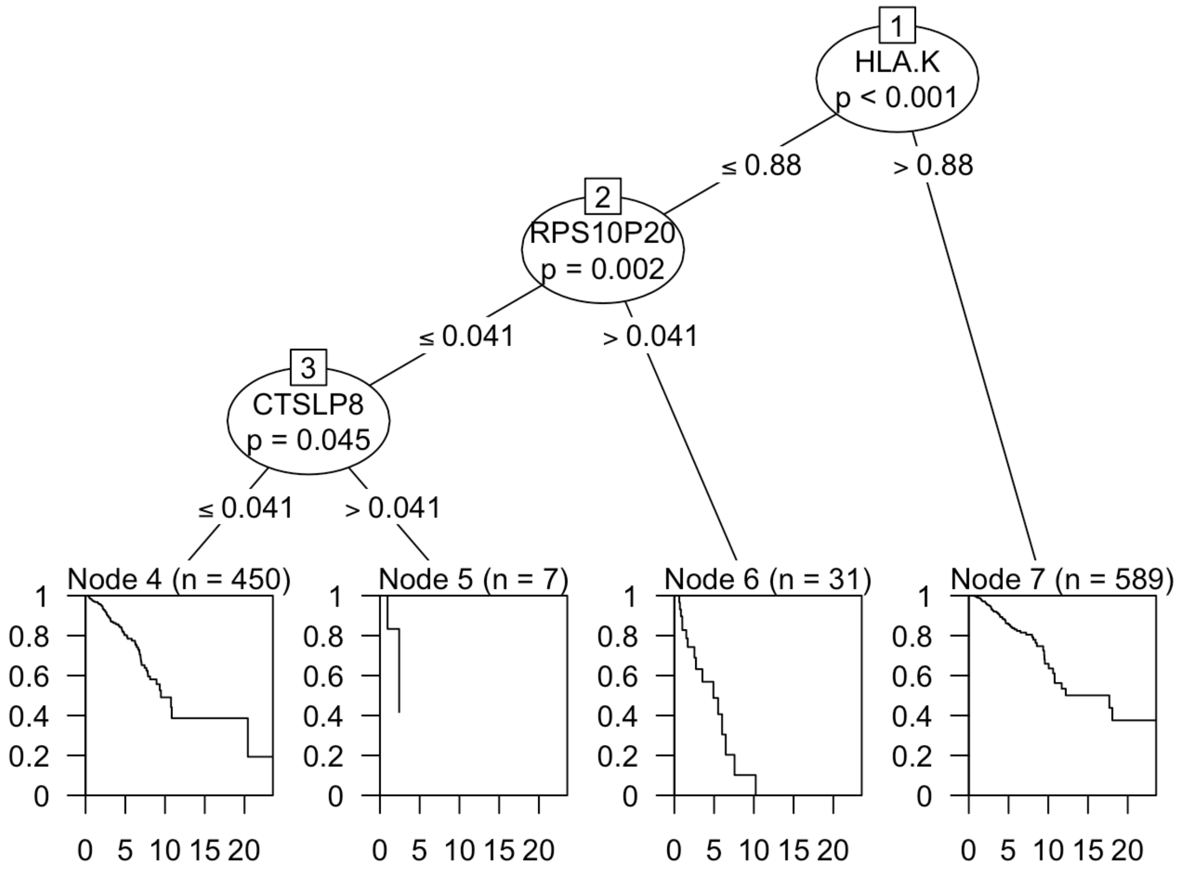


```
## Consider expression and clinical variables
BRCA_ctree3 <- ctree(Surv(survival.years, vital_status) ~ .,
                     data = data.frame(survival.years=surdata4$survival.years,
vital_status=surdata4$vital_status,
                                       pathologic_stage=surdata4$pathologic_stage,
gender=surdata4$gender,
                                       radiation_therapy=surdata4$radiation_therapy,
histological_type=surdata4$histological_type,
                                       race=surdata4$race,
ethnicity=surdata4$ethnicity,
                                       Pdat.01[, sel.lasso]))
plot(BRCA_ctree3)
```

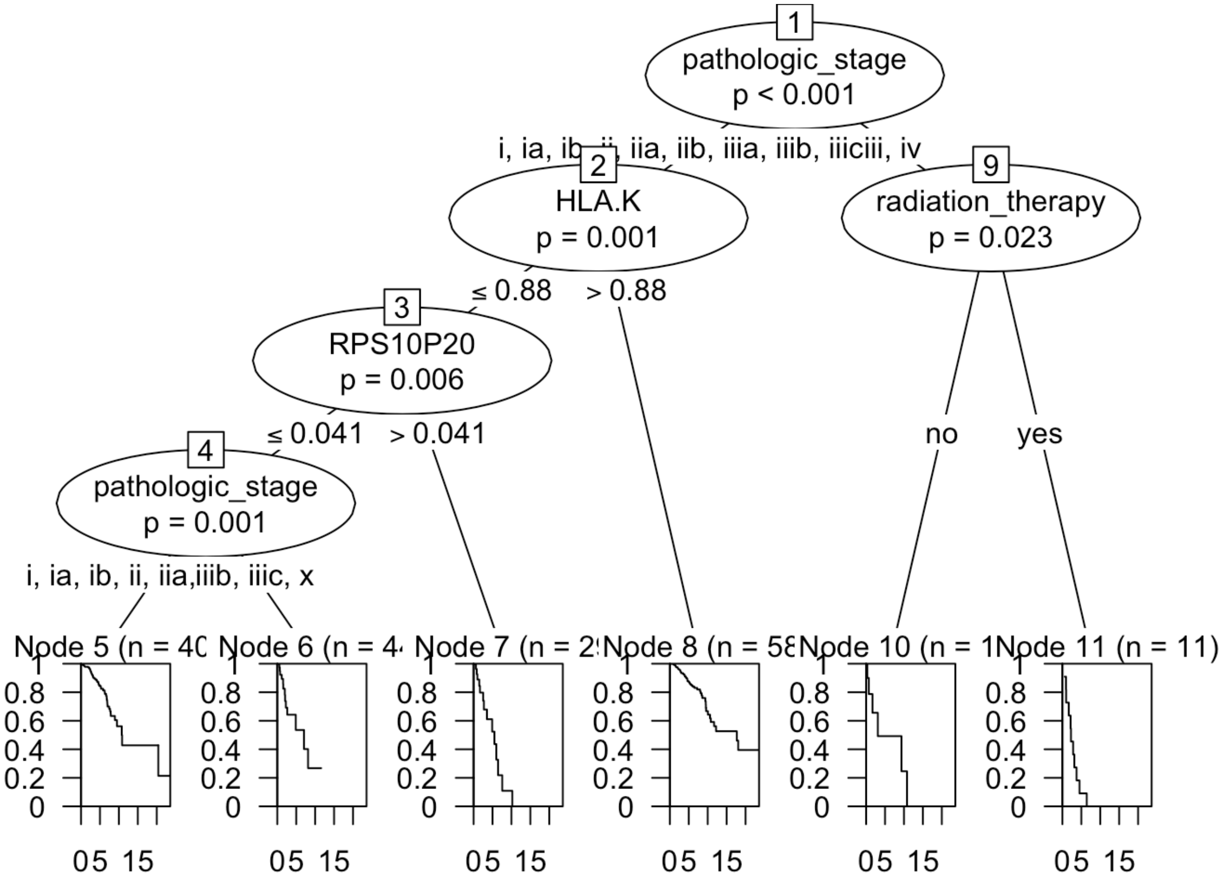


```
########################################
# Individual Pseudogene expression
########################################

## Split at median expression of CTSLP8
cox.CTSLP8 <- coxph(Surv(survival.years, vital_status) ~ Pdat.01[,"CTSLP8"], data =
surdata3)
summary(cox.CTSLP8)
## Call:
## coxph(formula = Surv(survival.years, vital_status) ~ Pdat.01[,
##     "CTSLP8"], data = surdata3)
##
##   n= 1077, number of events= 151
##
##                            coef exp(coef)  se(coef)     z Pr(>|z|)
## Pdat.01[, "CTSLP8"]     9.463 12878.231     2.342 4.041 5.33e-05 ***
## ---
## Signif. codes:  0 '***' 0.001 '**' 0.01 '*' 0.05 '.' 0.1 ' ' 1
##
##                     exp(coef) exp(-coef) lower .95 upper .95
## Pdat.01[, "CTSLP8"]     12878  7.765e-05     130.7   1268879
##
## Concordance= 0.514  (se = 0.011 )
## Likelihood ratio test= 8.14  on 1 df,   p=0.004
## Wald test            = 16.33  on 1 df,   p=5e-05
## Score (logrank) test = 23.44  on 1 df,   p=1e-06
CTSLP8.grps <- ifelse(Pdat.01[,"CTSLP8"] > median(Pdat.01[,"CTSLP8"]), 1, 0)
table(CTSLP8.grps)
## CTSLP8.grps
##    0    1
## 1045   32
```

```
km.CTSLP8 <- survfit(Surv(survival.years, vital_status) ~ CTSLP8.grps, data =
surdata3)
plot(km.CTSLP8, col = 1:2, xlab = "Time (years)",  ylab = "Survival Probability",
     lwd = 2, main = paste("CTSLP8 Expression and Survival"))
leg.txt <- c("Below median", "Above median")
legend("topright", leg.txt, lty = 1, col = 1:2, bty = "n", lwd = 2, title = "CTSLP8
Expression")
```

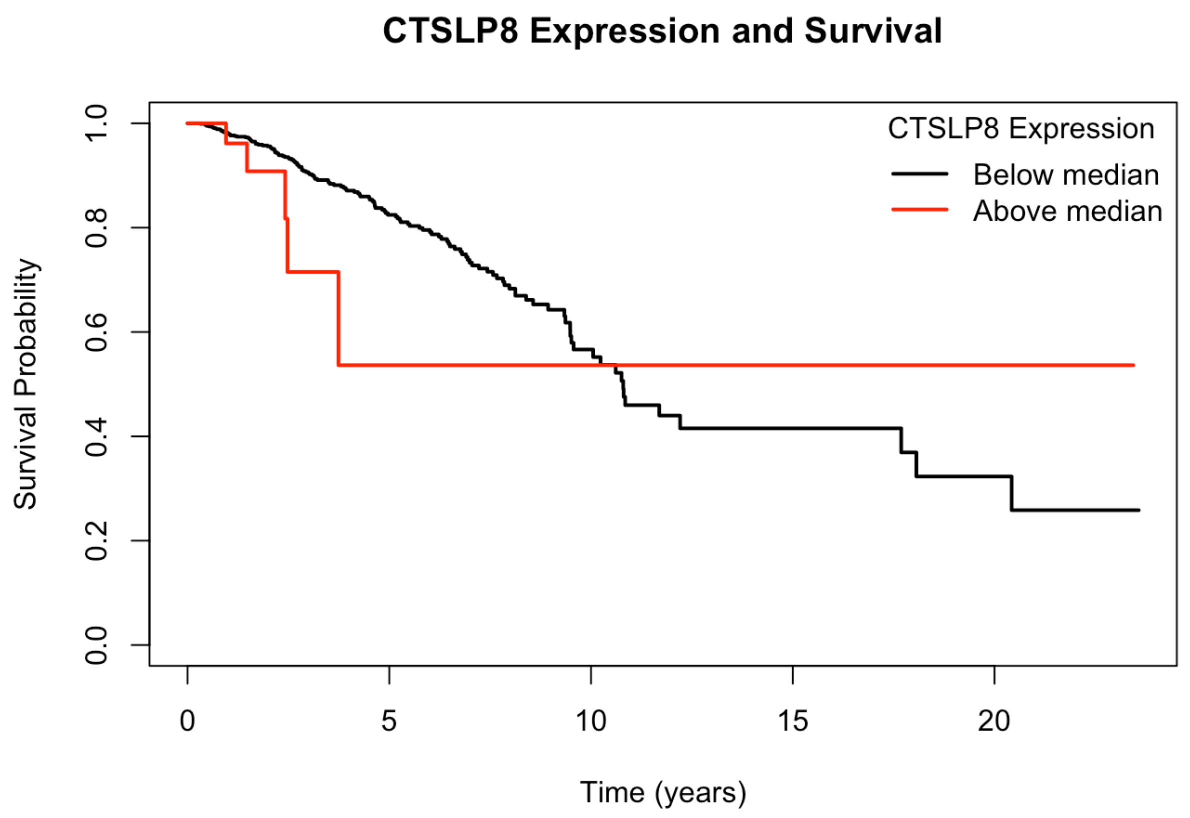


```
## Split at median expression of EEF1GP4
cox.EEF1GP4 <- coxph(Surv(survival.years, vital_status) ~ Pdat.01[,"EEF1GP4"], data =
surdata3)
summary(cox.EEF1GP4)
## Call:
## coxph(formula = Surv(survival.years, vital_status) ~ Pdat.01[,
##     "EEF1GP4"], data = surdata3)
##
##   n= 1077, number of events= 151
##
##                          coef exp(coef) se(coef)     z Pr(>|z|)
## Pdat.01[, "EEF1GP4"]  3.8838   48.6066   0.8013 4.847 1.25e-06 ***
## ---
## Signif. codes:  0 '***' 0.001 '**' 0.01 '*' 0.05 '.' 0.1 ' ' 1
##
##                      exp(coef) exp(-coef) lower .95 upper .95
## Pdat.01[, "EEF1GP4"]     48.61    0.02057     10.11     233.7
##
## Concordance= 0.519  (se = 0.024 )
## Likelihood ratio test= 14.09  on 1 df,   p=2e-04
## Wald test            = 23.49  on 1 df,   p=1e-06
## Score (logrank) test = 25.17  on 1 df,   p=5e-07
EEF1GP4.grps <- ifelse(Pdat.01[,"EEF1GP4"] > median(Pdat.01[,"EEF1GP4"]), 1, 0)
table(EEF1GP4.grps)
```

```
## EEF1GP4.grps
##   0   1
## 812 265
km.EEF1GP4 <- survfit(Surv(survival.years, vital_status) ~ EEF1GP4.grps, data =
surdata3)
plot(km.EEF1GP4, col = 1:2, xlab = "Time (years)",  ylab = "Survival Probability",
     lwd = 2, main = paste("EEF1GP4 Expression and Survival"))
leg.txt <- c("Below median", "Above median")
legend("topright", leg.txt, lty = 1, col = 1:2, bty = "n", lwd = 2, title = "EEF1GP4
Expression")
```

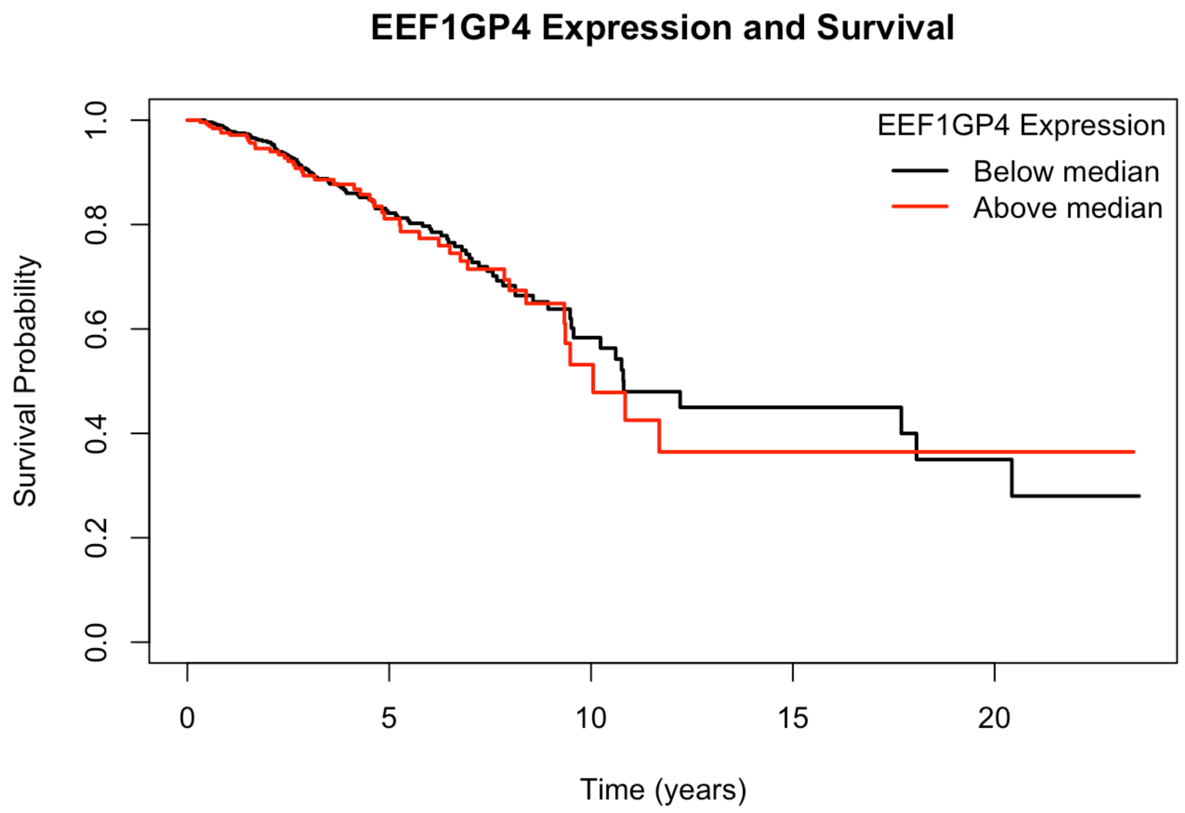


```
## Split at median expression of HLA-K
cox.HLAK <- coxph(Surv(survival.years, vital_status) ~ Pdat.01[,"HLA-K"], data =
surdata3)
summary(cox.HLAK)
## Call:
## coxph(formula = Surv(survival.years, vital_status) ~ Pdat.01[,
##     "HLA-K"], data = surdata3)
##
##   n= 1077, number of events= 151
##
##                         coef exp(coef) se(coef)      z Pr(>|z|)
## Pdat.01[, "HLA-K"] -0.8370    0.4330   0.2119 -3.95 7.82e-05 ***
## ---
## Signif. codes:  0 '***' 0.001 '**' 0.01 '*' 0.05 '.' 0.1 ' ' 1
##
##                    exp(coef) exp(-coef) lower .95 upper .95
## Pdat.01[, "HLA-K"]     0.433      2.309    0.2858    0.6559
##
## Concordance= 0.592  (se = 0.028 )
## Likelihood ratio test= 15.96  on 1 df,   p=6e-05
## Wald test            = 15.6  on 1 df,   p=8e-05
```

```
## Score (logrank) test = 15.75  on 1 df,   p=7e-05
HLAK.grps <- ifelse(Pdat.01[,"HLA-K"] > median(Pdat.01[,"HLA-K"]), 1, 0)
table(HLAK.grps)
## HLAK.grps
##   0   1
## 540 537
km.HLAK <- survfit(Surv(survival.years, vital_status) ~ HLAK.grps, data = surdata3)
plot(km.HLAK, col = 1:2, xlab = "Time (years)",  ylab = "Survival Probability",
     lwd = 2, main = paste("HLA-K Expression and Survival"))
leg.txt <- c("Below median", "Above median")
legend("topright", leg.txt, lty = 1, col = 1:2, bty = "n", lwd = 2, title = "HLA-K
Expression")
```

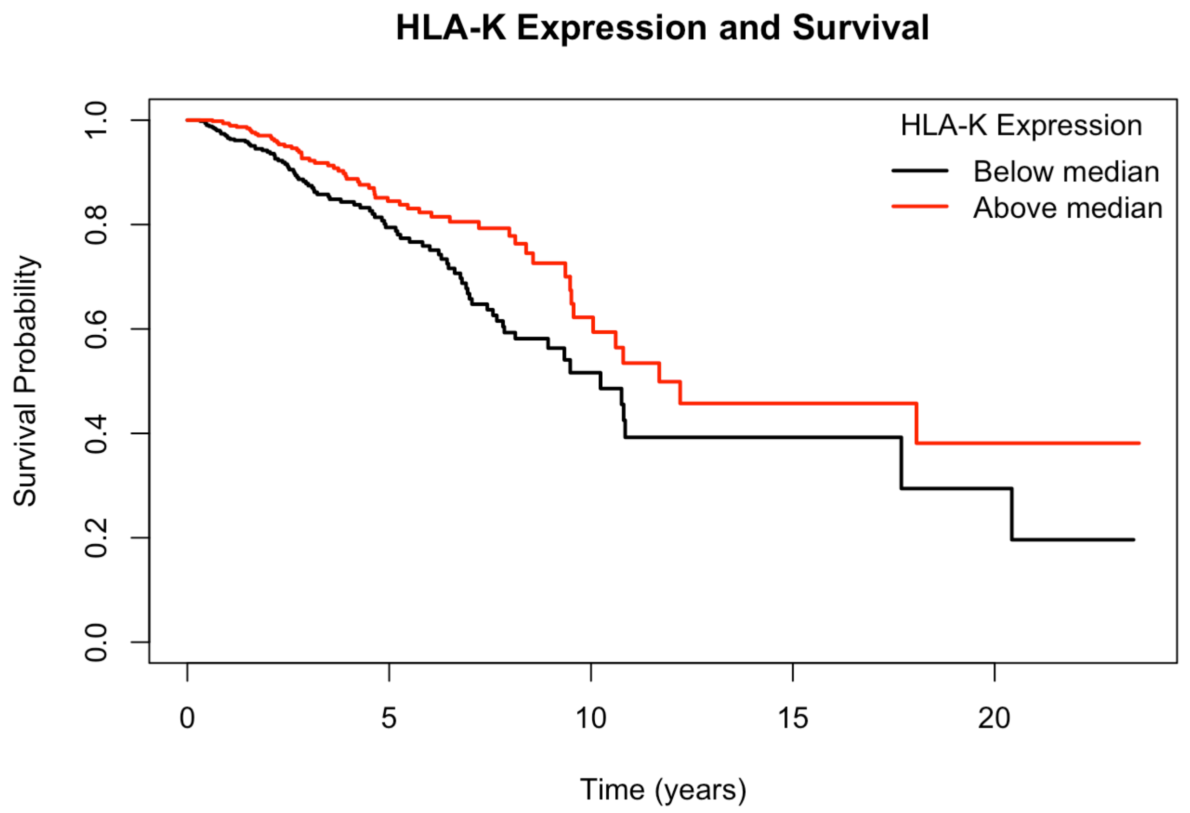


```
## Split at median expression of CBX1P3
cox.CBX1P3 <- coxph(Surv(survival.years, vital_status) ~ Pdat.01[,"CBX1P3"], data =
surdata3)
summary(cox.CBX1P3)
## Call:
## coxph(formula = Surv(survival.years, vital_status) ~ Pdat.01[,
##     "CBX1P3"], data = surdata3)
##
##   n= 1077, number of events= 151
##
##                          coef exp(coef) se(coef)     z Pr(>|z|)
## Pdat.01[, "CBX1P3"] 1.9693    7.1657   0.6069 3.245  0.00118 **
## ---
## Signif. codes:  0 '***' 0.001 '**' 0.01 '*' 0.05 '.' 0.1 ' ' 1
##
##                        exp(coef) exp(-coef) lower .95 upper .95
## Pdat.01[, "CBX1P3"]     7.166     0.1396     2.181     23.54
##
## Concordance= 0.544  (se = 0.026 )
```

```
## Likelihood ratio test= 8.36  on 1 df,   p=0.004
## Wald test            = 10.53  on 1 df,   p=0.001
## Score (logrank) test = 10.7  on 1 df,   p=0.001
CBX1P3.grps <- ifelse(Pdat.01[,"CBX1P3"] > median(Pdat.01[,"CBX1P3"]), 1, 0)
table(CBX1P3.grps)
## CBX1P3.grps
##   0   1
## 767 310
km.CBX1P3 <- survfit(Surv(survival.years, vital_status) ~ CBX1P3.grps, data =
surdata3)
plot(km.CBX1P3, col = 1:2, xlab = "Time (years)",  ylab = "Survival Probability",
     lwd = 2, main = paste("CBX1P3 Expression and Survival"))
leg.txt <- c("Below median", "Above median")
legend("topright", leg.txt, lty = 1, col = 1:2, bty = "n", lwd = 2, title = "CBX1P3
Expression")
```

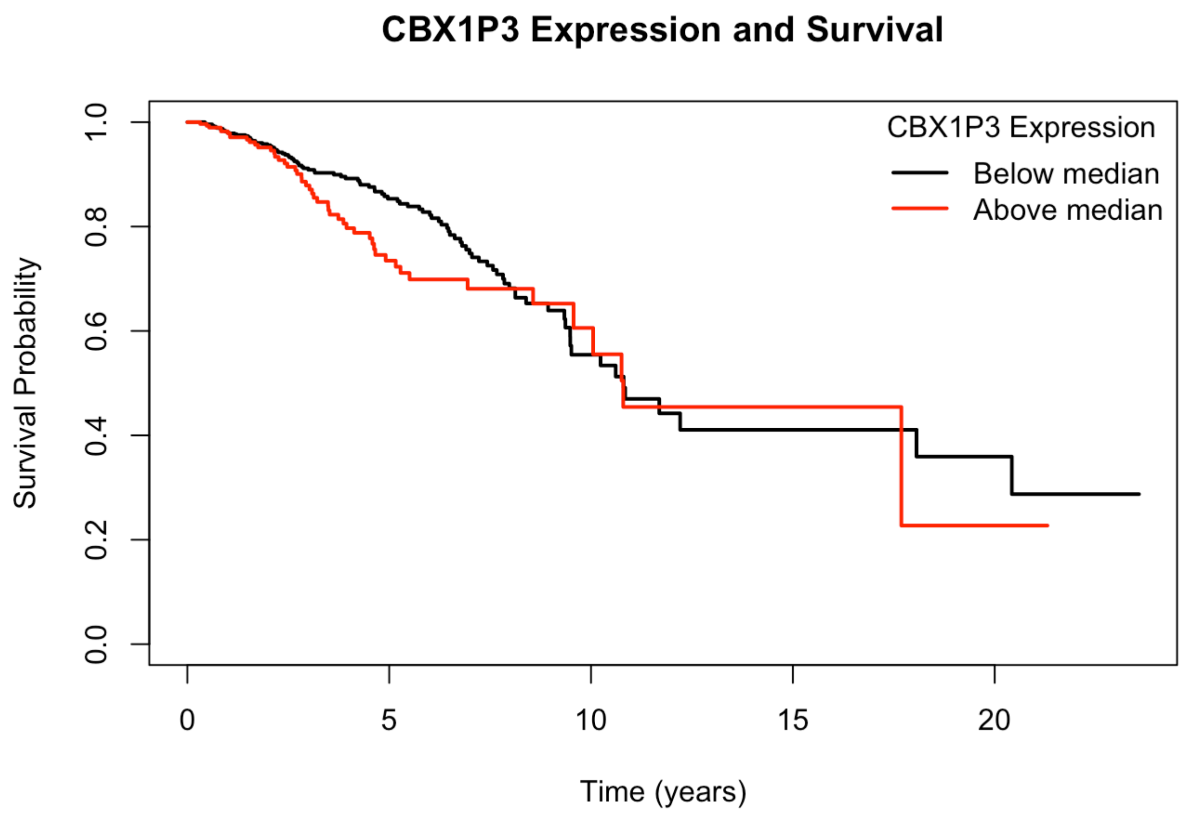


```
## Split at median expression of RPS10P20
cox.RPS10P20 <- coxph(Surv(survival.years, vital_status) ~ Pdat.01[,"RPS10P20"], data
= surdata3)
summary(cox.RPS10P20)
## Call:
## coxph(formula = Surv(survival.years, vital_status) ~ Pdat.01[,
##     "RPS10P20"], data = surdata3)
##
##   n= 1077, number of events= 151
##
##                          coef exp(coef) se(coef)     z Pr(>|z|)
## Pdat.01[, "RPS10P20"]   4.784   119.569    1.513 3.162  0.00157 **
## ---
## Signif. codes:  0 '***' 0.001 '**' 0.01 '*' 0.05 '.' 0.1 ' ' 1
##
##                       exp(coef) exp(-coef) lower .95 upper .95
```

```
## Pdat.01[, "RPS10P20"]      119.6   0.008363     6.164      2319
##
## Concordance= 0.538  (se = 0.018 )
## Likelihood ratio test= 7.48  on 1 df,   p=0.006
## Wald test            = 10  on 1 df,   p=0.002
## Score (logrank) test = 10.29  on 1 df,   p=0.001
RPS10P20.grps <- ifelse(Pdat.01[,"RPS10P20"] > median(Pdat.01[,"RPS10P20"]), 1, 0)
table(RPS10P20.grps)
## RPS10P20.grps
##   0   1
## 991  86
km.RPS10P20 <- survfit(Surv(survival.years, vital_status) ~ RPS10P20.grps, data =
surdata3)
plot(km.RPS10P20, col = 1:2, xlab = "Time (years)",  ylab = "Survival Probability",
     lwd = 2, main = paste("RPS10P20 Expression and Survival"))
leg.txt <- c("Below median", "Above median")
legend("topright", leg.txt, lty = 1, col = 1:2, bty = "n", lwd = 2, title = "RPS10P20
Expression")
```

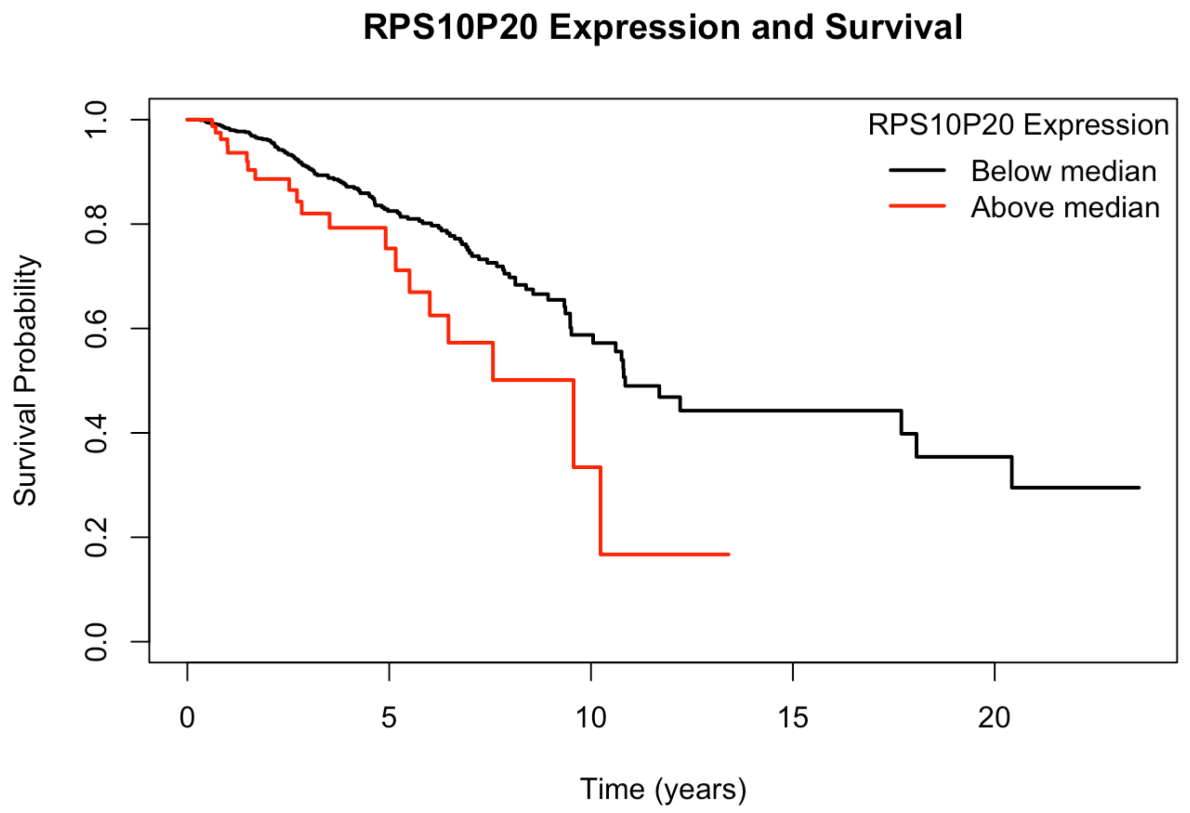


```
#########################################
# Combining Pseudogenes and genes
#########################################
common.patients = intersect(row.names(Pdat.01),row.names(RNA.counts.01))
mapping = read.csv("~/Desktop/ICIBM_2019/edgesblast_formatted.csv",header = FALSE,
stringsAsFactors = FALSE)
#load("~/Desktop/ICIBM_2019/annot.Rdata")

## Checking for overlap
cnt = 0
for (pseudo in pseudo.cox.top.unadjusted){
  if(pseudo %in% colnames(RNA.counts.01)){cnt=cnt+1;message("found")}
}
```

```
cnt # should be 0
## [1] 0
## Checking for overlap
cnt = 0
for (gene in gene.cox.top.unadjusted){
  if(gene %in% colnames(Pdat.01)){cnt=cnt+1;message("found")}
}
cnt # should be 0
## [1] 0
## Combining datasets with associated pseudogenes
tmp = RNA.counts.01[common.patients,gene.cox.top.unadjusted]
colnames(tmp) = sub("[|].*","",colnames(tmp))
RNA.g.pg.counts.01 = cbind(tmp,Pdat.01[common.patients,pseudo.cox.top.unadjusted])
for (pseudo in pseudo.cox.top.unadjusted){
  tmp = annot[annot$hgnc_symbol==pseudo,"ensembl_transcript_id"]
  if (!identical(tmp,character(0))){
    tmp = mapping[mapping[,2]==tmp,1]
    if (!identical(tmp,character(0))){
      tmp = unique(annot[annot$ensembl_gene_id %in% tmp,"hgnc_symbol"])
      if (!identical(tmp,character(0))){
        tmp = tmp[tmp %in% colnames(RNA.g.pg.counts.01)]
        if (!identical(tmp,character(0))){
          tmpmat = t(t(RNA.g.pg.counts.01[,tmp])*RNA.g.pg.counts.01[,pseudo])
          colnames(tmpmat) = paste(tmp,pseudo,sep="*");
          RNA.g.pg.counts.01 = cbind(RNA.g.pg.counts.01,tmpmat)
        }
      }
    }
  }
}

for (gene in sub("[|].*","",gene.cox.top.unadjusted)){
  tmp = annot[annot$hgnc_symbol==gene,"ensembl_gene_id"]
  if (!identical(tmp,character(0))){
    tmp = mapping[mapping[,1]==tmp,2]
    if (!identical(tmp,character(0))){
      tmp = unique(annot[annot$ensembl_transcript_id %in% tmp,"hgnc_symbol"])
      if (!identical(tmp,character(0))){
        tmp = tmp[tmp %in% colnames(RNA.g.pg.counts.01)]
        if (!identical(tmp,character(0))){
          tmpmat = t(t(RNA.g.pg.counts.01[,tmp])*RNA.g.pg.counts.01[,gene])
          colnames(tmpmat) = paste(gene,tmp,sep="*");
          RNA.g.pg.counts.01 = cbind(RNA.g.pg.counts.01,tmpmat)
        }
      }
    }
  }
}

RNA.g.pg.counts.01 = RNA.g.pg.counts.01[,unique(colnames(RNA.g.pg.counts.01))]
####################################################################
# fitting lasso cox model w/ gene pseudogene and interactions
####################################################################
surdata4 = surdata3[common.patients,]
S <- Surv(surdata4$survival.years, surdata4$vital_status)

### training data and validation data
set.seed(1000) # set the seed to ensure reproducibility

## Traing set rows
## randomly sample 2/3 of subjects
train.idx <- sample(1:nrow(surdata4), round(nrow(surdata4)*2/3), replace = FALSE)
length(train.idx) # 508
## [1] 508
nrow(surdata4) # 762
```

```
## [1] 762
X.train <- RNA.g.pg.counts.01[train.idx,]
S.train <- S[train.idx,]

## For test set rows use '-train.idx'
X.test <- RNA.g.pg.counts.01[-train.idx,]
S.test <- S[-train.idx,]

colnames(X.train) = sub("-","_",colnames(X.train))
colnames(X.test) = sub("-","_",colnames(X.test))
## penalized likelihood method using glmnet
set.seed(1000)

## Restrict starting to all significant genes based on univariate Cox model
## cv.glmnet does CV to determine the optimal lambda value
cv.lasso <- cv.glmnet(X.train, S.train, family = "cox", alpha=1)
plot(cv.lasso)
```

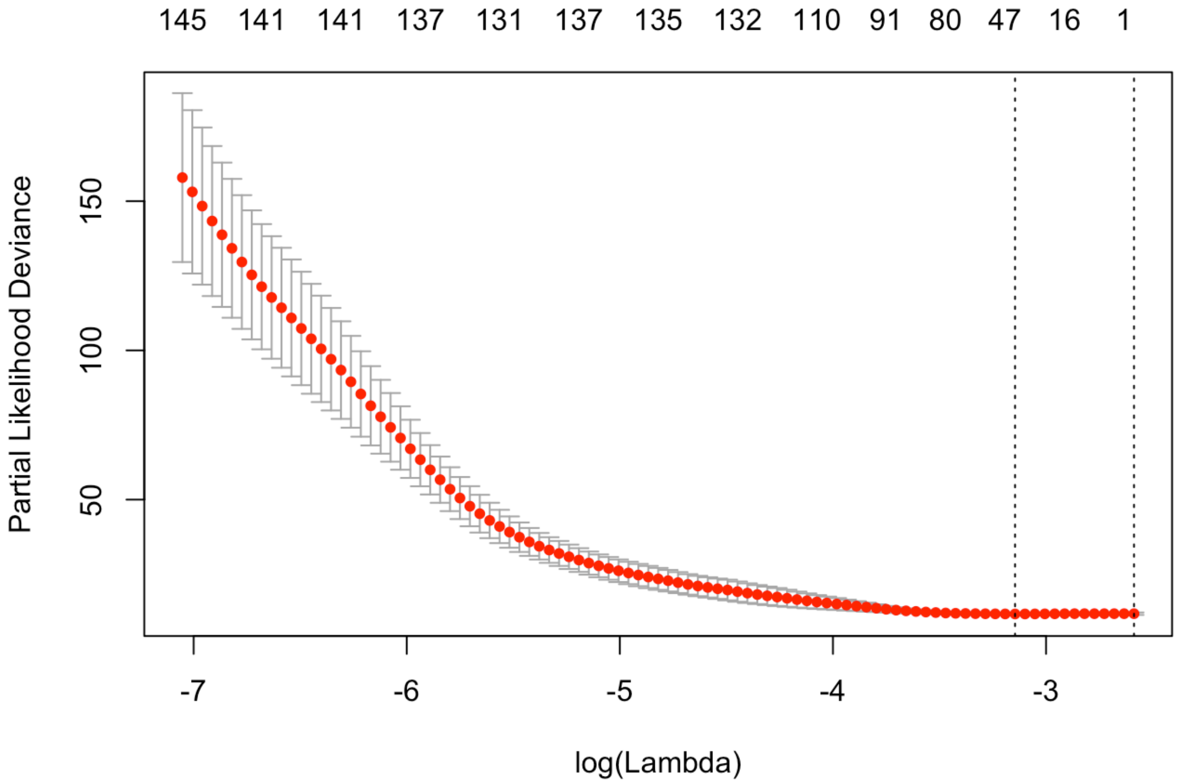


```
## How many predictors are selected at the optimal lambda value (lambda.min)?
coef.min <-  coef(cv.lasso, s = "lambda.min")
sum(coef.min != 0) # 39
## [1] 40
## lasso fit
coxfit.lasso <- glmnet(X.train, S.train, family = "cox", alpha=1)
plot(coxfit.lasso, xvar="lambda",label=TRUE)
abline(v=log(cv.lasso$lambda.min))
```

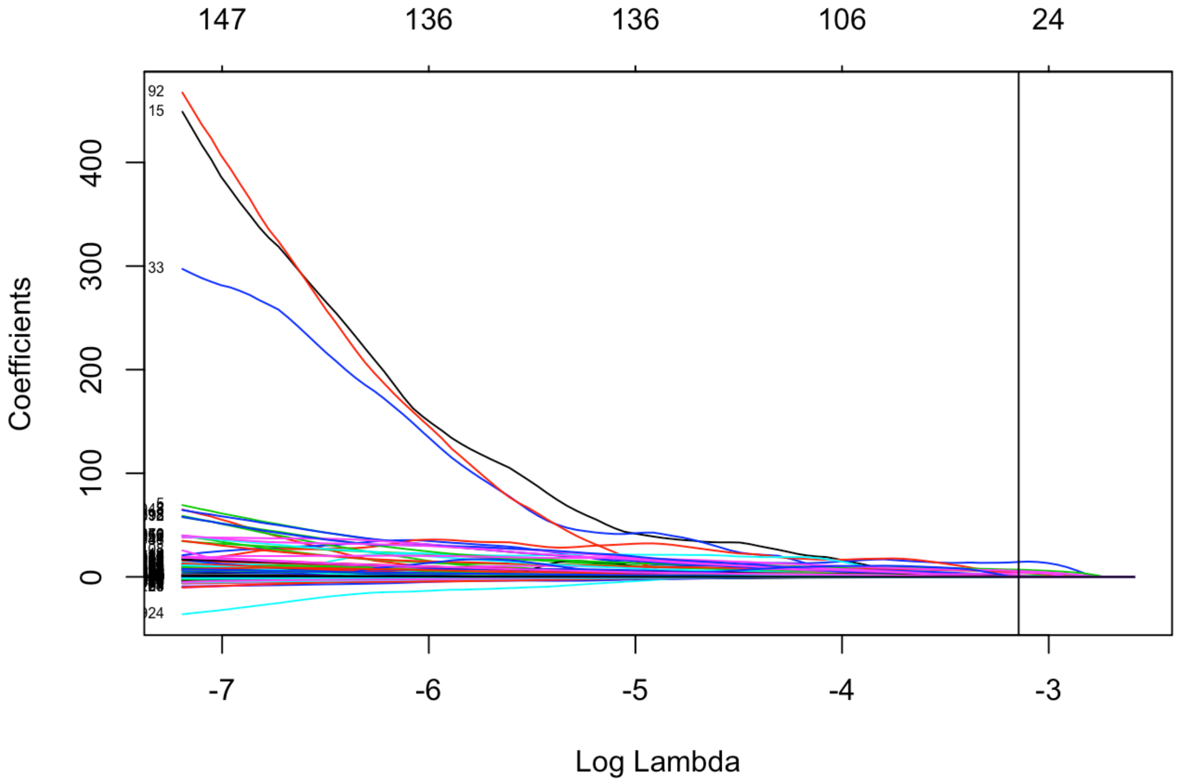


```
## genes selected with optimal model fitting
(coef.min <-  coef(cv.lasso, s = "lambda.min"))
## 1409 x 1 sparse Matrix of class "dgCMatrix"
##                                   1
## DEFB114                .
## SLITRK3                0.495993368
## SNORD114_9             .
## SNORD114_27            .
## GALP                   4.702272237
## OR4C13                 0.136788020
## ZPBP2                  .
## LOC143188              .
## KRT34                  .
## GALNT9                 .
## OR4C46                 .
## SI                     .
## LHFPL3                 .
## VN1R4                  0.219953349
## OR51F1                 .
## PWRN1                  .
## NDST4                  .
## LCE1A                  .
## LCE3C                 14.373617853
## BCHE                   .
## GPR123                 .
## FAM26D                 .
## STXBP5                 0.032217373
## LOC387646              .
## VSIG8                  .
## SPRR4                  .
## GRIA3                  .
```

```
## LOR                     .
## MUC21                   .
## GALNTL5                 .
## C13orf28                .
## SAA3P                   .
## OR8H3                   .
## TBC1D28                 .
## GLRA1                   .
## OR51A4                  .
## C4orf35                 .
## SP7                     .
## KCNMB2                  .
## ABCG4                   .
## CGB5                    .
## OR10Q1                  .
## NFKBIA                  .
## C10orf47                .
## SLC16A2                 .
## OR5B3                   .
## SNORD116_11             .
## SCG2                    .
## CFHR5                   .
## LCE3D                   .
## RAG2                    .
## MAPKAPK3                .
## IL1F10                  .
## LOC100128811            .
## TUBGCP5                 .
## CXorf66                 .
## LIPH                    .
## PRB3                    .
## ZNF25                   .
## SLCO6A1                 .
## IYD                     0.035734487
## SMR3B                   .
## HYAL3                   .
## CSNK1A1L                .
## C9orf79                 .
## CACNG8                  .
## CEBPD                   .
## PHKB                    .
## FMO9P                   .
## TRMT2B                  .
## LOC151658               .
## APOOL                   .
## CYP4F11                 .
## SERPINA1                .
## CLEC3A                  .
## GUCA2A                  .
## RPGRIP1L                .
## REG1B                   .
## ARID1B                  0.159598829
## GSTA3                   .
## TMEM31                  .
## TRDN                    .
## EEF1B2                  .
## EDA2R                   .
## FLJ34503                .
## C8orf75                 .
## CEACAM5                 .
## UPRT                    .
## FAM114A1                .
## OR5D18                  .
## RSPRY1                  .
## CRYGB                   .
```

```
## RPTN                    .
## C15orf50                .
## FLJ25328                .
## PTPMT1                  .
## POLR2C                  .
## SULT4A1                 .
## TREML2P1                .
## IFRD2                   .
## ATP2C2                  .
## TMEM8C                  .
## PHLDB2                  .
## KIAA2018                .
## DCD                     .
## SATL1                   .
## OR4L1                   .
## SNX9                    .
## PDSS2                   .
## CARS2                   .
## CHD9                    .
## OR56B4                  .
## TIGD7                   .
## ARL11                   .
## CCDC83                  .
## MRPL16                  .
## RBM15B                  .
## TAS2R38                 .
## ATXN8OS                 .
## RAPGEFL1                .
## CAB39L                  .
## XG                      .
## COPS6                   .
## LACE1                   .
## BAMBI                   .
## OR8I2                   .
## SNORA80                 .
## CLTA                    .
## MAP2K6                  .
## RPL27A                  .
## RPL29                   .
## CA1                     .
## LOC388946               .
## SMR3A                   0.047178844
## XGPY2                   .
## DIP2B                   0.002768274
## RYR2                    .
## TBC1D8B                 .
## SYNJ2                   .
## MIA2                    .
## NOS2                    .
## VWA5B2                  .
## TULP4                   .
## OTX2                    .
## HNRNPC                  .
## MAP3K13                 .
## TMEM188                 .
## C6orf35                 .
## ONECUT1                 .
## GPR152                  .
## RPL14                   .
## CYP4B1                  .
## LELP1                   .
## PCNAP1                  .
## PIK3CA                  .
## ADAM32                  .
## C1orf49                 .
```

```
## PINK1                  .
## CISH                   .
## FGA                    .
## BCAS3                  .
## TAAR2                  .
## LPAL2                  .
## ERLIN2                 .
## ZNF443                 .
## MCTS1                  .
## PSME2                  .
## PRR4                   .
## LOC100101266           0.008372821
## C2orf39                .
## PROSC                  .
## SDAD1                  .
## PCDHA6                 .
## SEC63                  .
## APOH                   .
## TAS2R7                 .
## MORC4                  .
## LOC100190940           .
## CTXN1                  .
## ZNF611                 .
## C1orf127               .
## PCDHA5                 .
## XRCC4                  .
## SCARB2                 .
## KIR3DL3                .
## MEA1                   .
## KIF5B                  .
## C16orf70               .
## MORF4L2                .
## TIMM10                 .
## PLCL1                  .
## FOXA3                  .
## FAM192A                .
## RPS24                  .
## ERRFI1                 .
## RPL27                  .
## GABRG3                 .
## OTUD3                  .
## FER1L5                 .
## RCL1                   .
## FKBP5                  .
## CPT1A                  .
## TTPA                   .
## C3orf75                .
## ADORA1                 .
## C3orf45                .
## PCDHA8                 .
## OCRL                   .
## SH2B2                  .
## AAK1                   .
## SRGAP1                 .
## MAPK10                 .
## C20orf194              .
## PRMT8                  .
## PWRN2                  .
## CDK19                  .
## KCNJ13                 .
## PIRT                   .
## SNORD116_14            .
## POLR1D                 .
## SECISBP2L              .
## ITFG1                  .
```

```
## C19orf66            -0.008675519
## FOXJ1                 .
## OR1M1                 .
## OR4F21                .
## C12orf12              .
## STX7                  .
## C4orf12               .
## B4GALT1               .
## SNAR_E                .
## UNKL                  .
## RAPSN                 .
## PCDHA13               .
## ZNF75D                .
## HINT2                 .
## SNORD115_14           .
## TTC21B                .
## ARMC3                 .
## NENF                  .
## ZNF449                .
## TRMT2A                .
## GRHPR                 .
## WWOX                  .
## PCDHAC2               .
## GLOD5                 .
## CPSF4                 .
## ACBD5                 .
## SLC35A5               .
## PCDHGA2               .
## SLC38A7               .
## DPY19L3               .
## EIF4G3                .
## OR5A2                 .
## ROPN1L                .
## RPSA                  .
## C1orf226              .
## RRN3P2                .
## ZMAT3                 .
## MBTPS1                .
## C21orf57              .
## BST2                  .
## ZNF137                .
## CCDC72                .
## CGB8                  .
## RPL38                 .
## PCDHAC1               .
## GPR27                 .
## YIPF6                 .
## ASB15                 .
## SPRR2B                .
## TMEM45A               .
## C11orf85              .
## LSG1                  .
## PARP12               -0.114099946
## C6orf108              .
## WDSUB1                .
## C4orf45               .
## ANO6                  .
## XIAP                  .
## GALK2                 .
## ARL2BP                .
## HIST2H2AC             .
## C8orf12               .
## DNAJB5                .
## ZNF490                .
## ENPP5                 .
```

```
## C9orf163          .
## PEX10             .
## NCOA2             .
## GOT2              .
## CYP2C9            .
## EXOC1             0.058222957
## SH3BP1            .
## FAM120B           .
## OSBPL2            .
## SNORD115_22       .
## ?                 .
## CEL               0.012217098
## CEACAM6           .
## SLC9A1            0.146633398
## C12orf66          .
## NRK               .
## ELMOD3            .
## SS18L2            .
## OR6X1             .
## RB1               .
## C4orf37           .
## MORN3             .
## BBX               .
## UBE4B             .
## ZNF791            .
## TEKT2             .
## TUBA3D            .
## QARS              .
## PCDHGB2           .
## CCDC28B          -0.015727118
## ATP5G1            .
## GLT6D1            .
## SLC45A2           .
## SEMA3B            .
## ZNF480            .
## IGFBPL1           .
## MANF              .
## BRD7              .
## KCNMB3            .
## KRTAP19_2         .
## ADAMTS8           .
## AKTIP             .
## POU5F1B           .
## IFI27L1           .
## MTRF1L            .
## JRKL              .
## HRC               .
## RAP1GAP2          .
## RGS3              .
## SNORD37           .
## PPFIBP2           .
## RPS18             .
## IFITM1            .
## C1orf228          .
## SIAH2             .
## PCDHGA5           .
## POF1B             .
## NSUN3             .
## ZNF37A            .
## C9orf84           .
## ATG4A             .
## ATP5J2            .
## RBM41             .
## SNORD38A          .
## PLEKHA4           .
```

```
## N4BP1                    .
## ATF7IP                   .
## BAP1                     .
## NMUR2                    .
## BCL8                     .
## FAIM3                    .
## REEP2                    .
## AP1G1                    .
## OR52A4                   .
## MFAP3L                   .
## UBE2L6                   .
## YWHAB                    .
## OPHN1                    .
## ZNF33A                   .
## TAF3                     .
## KRT78                    .
## LACRT                    .
## ZMYND8                   .
## ACP2                     .
## C11orf30                 .
## TUBA4B                   .
## PUS7L                    .
## C20orf94                 .
## NKAIN1                   .
## SHISA5                   .
## TAF7                     .
## PNPLA3                   .
## BPIL3                    .
## EP400NL                  .
## GUCY1B2                  .
## SNORD62A                 .
## MBD5                     .
## GUCA1C                   .
## DCDC2B                   .
## THAP7                    .
## ACSS3                    .
## DNAJC27                  .
## GTPBP1                   .
## MGC4473                  .
## PAK6                     .
## RPL23A                   .
## ZNF28                    .
## TM4SF18                  .
## SRPK3                    .
## LIMCH1                   .
## TAF10                    .
## CLU                      .
## C10orf18                 .
## C4orf22                  .
## NIPA2                    .
## RRN3P3                   .
## ZNF493                   .
## HSP90AB2P                .
## OR13C8                   .
## NUP43                    .
## LOC732275                .
## RBL2                     .
## C1orf53                  .
## INSIG2                   .
## FLJ35220                 .
## BTBD6                    .
## GUCY2E                   .
## PTMA                     .
## R3HDM2                   .
## SMARCD3                  .
```

```
## BRWD3                  .
## DGAT2L6                .
## ZNF248                 .
## CMTM4                  .
## C6orf142               .
## HINT3                  .
## HERPUD1                .
## DLG3                   .
## LOC728323              .
## FAU                    .
## TOMM22                 .
## GPR132                 .
## PCDHA9                 .
## OR9K2                  .
## LOC651250              .
## SLC1A1                 .
## DKK2                   .
## MT1E                   .
## PICALM                 .
## PCMT1                  0.088607253
## TBCEL                  .
## GRIP1                  .
## CSN3                   .
## SBF1P1                 .
## C10orf131              0.015948551
## GABARAPL2              .
## NGDN                   .
## PABPN1                 .
## HARS2                  0.077180043
## MT1G                   .
## FAM63B                 .
## PPIB                   .
## PNKD                   .
## CA5B                   .
## FAM172A                .
## EIF2AK3                .
## LRRC69                 .
## FAM128A                .
## VANGL1                 .
## YIPF5                  .
## KRTAP12_1              .
## SGCA                   .
## CHCHD1                 .
## LOC284900              .
## TBK1                   .
## CFHR3                  .
## SAMD1                  .
## PCDHGA12               .
## TMC7                   .
## FAM199X                .
## ZFP3                   .
## CXCL13                 .
## MORN5                  .
## RPS8                   .
## SDCBP2                 .
## PCSK9                  .
## RPL4                   .
## VPS24                  .
## C6orf138               .
## MED28                  .
## ZNF33B                 .
## POM121L9P              .
## FGFBP1                 .
## WDR38                  .
## FAM179A                .
```

```
## ANAPC11                .
## DDX4                   .
## PSMC3                  .
## TAS2R10                .
## MRO                    .
## NKAIN2                 .
## PRB4                   .
## CEBPB                  .
## PTGES3                 .
## SYTL2                  .
## CELP                   0.369951542
## S1PR2                  .
## HHLA3                  .
## PCDHGB3                .
## TMEM184C               .
## SERPINA12              .
## PCDHGB1                .
## ACRC                   .
## SCRN3                  .
## S100A5                 .
## EZR                    .
## LONP2                  .
## BPIL1                  .
## PKD2L2                 .
## STAC2                  .
## NAB2                   .
## C7orf53                0.071648040
## SNRPA                  .
## NDP                    .
## TOMM5                  .
## TFDP1                  .
## SPINK8                 .
## IRS2                   .
## ABHD14A                .
## ZNF646                 .
## DNAJA2                 .
## RPL31                  .
## B4GALNT4               .
## CCDC74B                .
## ENPP7                  .
## PLEKHG4                .
## POLE4                  .
## KRTAP10_1              .
## RPS13                  .
## STOML2                 .
## PDCD7                  .
## PRKG1                  .
## LRRC10B                .
## HADHB                  .
## RPS9                   .
## HNRNPD                 .
## PCDHB16                .
## ZNF160                 .
## SH3BGRL                .
## ZNF672                 .
## RSPH3                  .
## MGC57346               .
## CCDC116                .
## C6orf81                .
## CCL3L1                 .
## C11orf31               .
## UBQLN3                 .
## DPPA5                  .
## CLOCK                  .
## NFKBIE                 .
```

```
## RFT1                .
## DKFZp566F0947       .
## CHD6                .
## MTP18               .
## XKR7                .
## FLG2                .
## KCNH3               .
## MAGEB18             .
## KIAA1328            .
## SLC17A5             .
## RPLP2               .
## ZNF75A              .
## GPR157              .
## SPAG8               .
## IL27RA              .
## MGA                 .
## SLC20A2             .
## SERINC3             .
## FRMD7               .
## FAM184B             .
## PEX5L               .
## GOLGA2              .
## IL24                .
## C9orf103            .
## NOS1                .
## OR5AN1              .
## RPL19               .
## STAG2               .
## TFPI2               .
## RPL8                .
## RPS17               .
## TCEAL6              .
## PIGR                .
## APC                 .
## VPS8                .
## PCDHA10             .
## ZNF843              .
## TRY6                .
## NT5C1A              .
## ZNF431              .
## ERCC6               .
## ZNF182              .
## PDP1                .
## RBM45               .
## PELO                .
## GOPC                .
## MCART2              .
## PDZD2               .
## PCDHB2              .
## NCRNA00182          .
## RABL3               .
## DAZAP2              .
## HMGCS2              .
## ZNF564              .
## NR1H3               .
## AIG1                .
## MIOS                .
## LOC646214           .
## ZNF638              .
## ZNF81               .
## TCEAL1              .
## KCND2               .
## C17orf72            .
## LAMP2               .
## TRIM21              .
```

```
## KRTAP10_2          .
## NDUFAF3            .
## CTSF               .
## OR5D14             .
## ATP7A              .
## CCDC107            .
## LRRN3              .
## TJP1               .
## DUSP5P             .
## KLHDC7B            .
## CYP2A6             .
## TM2D2              .
## LDB3               .
## SLC30A5            .
## BEND5              .
## SPATS1             .
## ACTC1              .
## GYPA               .
## WDR44              .
## ADAMTSL3           .
## REM2               .
## MKLN1              .
## FGGY               .
## C10orf118          .
## VPS35              .
## VEGFB              .
## MTM1               .
## TP73               .
## KDELR1             .
## GPS2               .
## PLA2G1B            .
## C20orf85           .
## NKAP               .
## HRAS               .
## SNORA16A           .
## SLC38A2            .
## SPINK9             .
## PGK1               .
## OSBPL10            .
## RIMBP2             .
## ZFHX3              .
## LOC100130238       .
## ENOX2              .
## LOC100216001       .
## C1orf161           .
## CCDC125            .
## EIF4E3             .
## NHSL2              .
## LOC100128977       .
## PXMP4              .
## PCYOX1             .
## RAB18              .
## ZBTB2              .
## SOCS6              .
## PLEKHO1            .
## UBXN11             .
## SETD7              .
## C3orf51            .
## SLC24A4            .
## CARD14             .
## ANKS1B             .
## C16orf82           .
## MAP7D2             .
## PDLIM4             .
## CST9L              .
```

```
## SLC45A1                .
## SPHK1                  .
## MDK                    .
## MED17                  .
## STK24                  .
## RPS27                  .
## HES5                   .
## TMEM22                 .
## ACSL1                  .
## HNRNPA1L2              .
## DPYSL4                 .
## BAZ2B                  .
## PDHB                   .
## INTS4L1                .
## SEPT5                  .
## ACAP2                  .
## C1orf101               .
## OSTM1                  .
## ODZ3                   .
## TAF1                   .
## MRPL23                 .
## SNORD57                .
## CSNK1A1                .
## ZNF765                 .
## USH1G                  .
## PRH1                   .
## FADS3                  .
## SHISA9                 .
## PRRC1                  .
## CITED4                 .
## LPAR4                  .
## OR5B17                 .
## ALDH3A1                .
## ZNF506                 .
## SAMD8                  .
## SIAH1                  .
## FAM98B                 .
## C12orf50               .
## GNG5                   .
## HEPHL1                 .
## ZNF254                 .
## DHDPSL                 .
## NDUFB1                 .
## CPO                    .
## FAM171B                .
## FAM127C                .
## HCCS                   .
## ZNF720                 .
## GABRG2                 .
## CGB2                   .
## SNX2                   .
## NAT6                   .
## STRA6                  .
## INO80                  .
## MED19                  .
## PRKAB2                 .
## PAFAH1B1               .
## RANBP1                 .
## GNS                    .
## RAB5B                  .
## AP4E1                  .
## PGM5                   .
## TPTE2                  .
## SUSD3                  .
## HSPB7                  .
```

```
## ZNF320             .
## CDH17              .
## CDHR4              .
## CSPG5              .
## LOC729467          .
## CACNA1H            .
## TUT1               .
## SERINC1            .
## CNIH2              .
## PGBD3              .
## DKFZP686I15217     .
## LOC642587          .
## RABGAP1            .
## OR3A1              .
## ZCCHC17            .
## SLC2A10            .
## RAP1GAP            .
## SPOPL              .
## OR4D2              .
## B4GALT2            .
## ZNF41              .
## TTLL2              .
## ST8SIA3            .
## CUX1               .
## PTPRR              .
## SEPT3              .
## ZNF468             .
## MAGT1              .
## AMTN               .
## PLBD2              .
## CHIC1              .
## ZCCHC14            .
## COG6               .
## OXA1L              .
## PCDHA1             .
## KRTAP4_9           .
## CDKN2C             .
## C2orf42            .
## USP8               .
## MAP1LC3B           .
## TRAPPC10           .
## DSG4               .
## SBNO1              .
## PARK2              .
## MEGF10             .
## EPC1               .
## C1GALT1C1          .
## PTPLA              .
## GPR153             .
## DIP2C              .
## KIF9               .
## GCNT7              .
## OR8U1              .
## ADAMTSL1           .
## C20orf165          .
## NME2P1             .
## C11orf49           .
## PCDHGB5            .
## MRPL34             .
## ZNF766             .
## ZBBX               .
## PPP1R1C            .
## LOC153910          .
## PCSK2              .
## GABARAPL3          .
```

```
## MECP2                    .
## ATP9B                    .
## RSC1A1                   .
## LOC100129055             .
## ZNF674                   .
## TBC1D20                  .
## HTR7P1                   .
## CNTN6                    .
## UBE2G1                   .
## ZNF347                   .
## LRRC48                   .
## NQO2                     .
## THOC2                    .
## NEK10                    .
## PIGN                     .
## LOC100133920             .
## CCDC74A                  .
## JMY                      .
## AHCY                     .
## ZC3H13                   .
## KIAA0494                 .
## ADAM9                    .
## UBE3A                    .
## ZNF836                   .
## SNORA9                   .
## MATK                     .
## RAPGEF4                  .
## EGR3                     .
## TMEM11                   .
## TMED6                    .
## SPA17                    .
## TIRAP                    .
## ASB4                     .
## NMU                      .
## LMBR1L                   .
## DSTN                     .
## PCDHGA1                  .
## HTA                      .
## NBEAL1                   .
## MYEOV2                   .
## DDI2                     .
## GLG1                     .
## ICAM4                    .
## DDI1                     .
## ARHGAP12                 .
## PSG4                     .
## EN2                      .
## TMCO7                    .
## C15orf21                 .
## CASC4                    .
## KCNH1                    .
## TMEM14B                  .
## MDGA1                    .
## OR52E6                   0.119397462
## KRTCAP3                  .
## PRDXDD1P                 .
## SRF                      .
## CLIP1                    .
## NPRL2                    .
## C1orf91                  .
## ZNF321                   .
## SELK                     .
## DEFB121                  .
## SNORD116_24              .
## RGS9BP                   .
```

```
## SLC25A43          .
## KCNK9             .
## PDE10A            .
## ZNF263            .
## KIAA1024          .
## FLJ37307          .
## PER4              .
## EIF5AL1           .
## GPR151            .
## LOC100132724      .
## DDIT4             .
## FIBCD1            .
## SNORD11B          .
## ACTN2             .
## BBS2              .
## RSF1              .
## POU3F3            .
## SNORD34           .
## RAD50             .
## RNF183            .
## TMEM9             .
## PDE11A            .
## C1orf86           .
## PCDHGA6           .
## FBXW12            .
## HIPK2             .
## ZNF808            .
## SNX3              .
## OR13C2            .
## PCDHA4            .
## OSCP1             .
## PTPDC1            .
## FAM9C             .
## CDR2              .
## SNX22             .
## PCDHA2            .
## CCDC33            .
## CALCR             .
## CATSPER3          .
## CDH6              .
## DGKZ              .
## SDR39U1           .
## RPL37             .
## OR5AC2            .
## YPEL5             .
## SLC25A36          .
## CDH4              .
## DHRS13            .
## MANSC1            .
## TACC2             .
## PXDNL             .
## HBII_52-45        .
## JAKMIP3           .
## AK2               .
## EIF3F             .
## IGFALS            .
## TNFAIP2           .
## ENTPD5            .
## OR1D2             .
## PHF20             .
## ATF6B             .
## RGS17             .
## ZNF845            .
## ARC               .
## MOBKL1A           .
```

```
## BFSP1                    .
## USP9X                    .
## NT5DC3                   .
## HBS1L                    0.016464583
## RPL29P2                  .
## FADS1                    .
## LOC145837                .
## PGM5P2                   .
## GPR34                    .
## RPLP0                    .
## C1orf194                 .
## LOC644172                .
## IER3                     .
## USP46                    .
## MID2                     .
## OSTalpha                 .
## INO80D                   .
## PSPC1                    .
## ZNF429                   .
## ZNF323                   .
## FASN                     .
## TMEM223                  .
## GPHA2                    .
## PPP1R11                  .
## ZNF525                   .
## DND1                     .
## CPAMD8                   .
## FTSJD1                   .
## CPSF7                    .
## ZNF350                   .
## CYFIP1                   .
## ING2                     .
## TUBA3E                   .
## PTPN20A                  .
## PRDM10                   .
## GLYATL1                  .
## PRDM2                    .
## GPR3                     .
## IL22RA1                  .
## CARHSP1                  .
## COX6A2                   .
## ABCA1                    .
## GNRH2                    .
## TMEM69                   .
## KCNH6                    .
## GPR81                    .
## FAM178A                  .
## TEX264                   .
## C9orf23                  .
## FSD1                     .
## DNAJC13                  .
## ZNF510                   .
## FMN2                     .
## TBC1D3P2                 .
## ATG2B                    .
## ELP2P                    .
## C2CD2                    .
## SDC3                     .
## DCUN1D4                  .
## FAM47A                   .
## DPY19L1                  .
## MAP1LC3B2                .
## ATRN                     .
## SCFD2                    .
## EREG                     .
```

```
## CYP2A7                 .
## HERC2                  .
## CXXC4                  .
## MAFF                   .
## MXI1                   .
## LDLRAD1                .
## HEATR3                 .
## HNRNPCP9               .
## OR4C7P                 .
## CTSLP8                 .
## VN1R6P                 .
## EEF1GP4                .
## SUMO2P2                .
## BNIP3P23               .
## FAM60DP                .
## MEMO1P3                6.917183970
## PRSS3P4                .
## HLA_K                 -0.085305142
## CBX1P3                 .
## GVINP2                 0.127259216
## RPS10P20               1.043166033
## MTND4LP1               .
## POM121L4P              .
## ISCA1P4                .
## CD24P4                 .
## CCDC74BP1              .
## BTF3P15                .
## HSPA8P1                1.238712267
## GAPDHP45               1.550053891
## HSP90AA2P              .
## TLE1P1                 .
## ANKRD30BP2             0.805817560
## MLLT10P2               .
## LAPTM4BP2              .
## HIGD1AP1               .
## RPL23AP28              .
## GOLGA8VP               .
## RPL13P12               .
## BANF1P5                .
## SLC25A24P2             .
## BNIP3P1                .
## TERF1P4                .
## UBE2L2                 .
## RPS20P25               4.134368532
## RPSAP39                .
## KRT18P62               0.260231002
## HSP90AA6P              .
## SEPT14P13              .
## TDGF1P7                .
## VN1R3                  .
## NCOR1P1                .
## MRPS18AP1              .
## TAF9BP1                .
## SEPT7P9                .
## PES1P2                 .
## TPRXL                  .
## DNAJC19P1              .
## IGHV3_22               .
## NDUFS5P6               .
## MEMO1P1                .
## MRPS10P1               .
## IGHV1OR16_1            .
## LDHAP5                 .
## EIF2S2P4               .
## RPS26P45               .
```

```
## KRT8P41               .
## FAM183BP              .
## KRT8P17               .
## VDAC1P2               .
## OR7E83P               .
## OR7E10P               5.583227212
## KRT16P2               .
## POM121L6P             .
## DYNLL1P6              .
## MARK2P12              .
## HSP90B3P              .
## KRT18P29              .
## BNIP3P28              .
## PSME2P2               .
## GGTLC4P               .
## RPL4P1                .
## IGLC6                 .
## TMEM198B              .
## SCML2P1               0.123924507
## MRPS21P2              .
## RPS4XP12              .
## FAM157A               .
## XRCC6P1               .
## BCAP31P1              .
## TP53TG3GP             .
## TUBAP2                .
## SAPCD2P3              .
## HNRNPA1P48            .
## FAM91A3P              .
## PAIP1P1               .
## HCG4                  .
## OR8R1P                .
## PTP4A1P1              .
## RPL5P34               .
## HMGN2P25              .
## SMPD4P1               .
## PPIAP11               .
## TP73_AS1              .
## IGHV1OR15_4           .
## LRRC37A6P             .
## CNN3P1                .
## TAGLN2P1              .
## PFN1P2                .
## IGJP1                 .
## CD46P1                .
## PRR20FP               .
## RPL5P28               1.226227509
## HCG4P7                .
## LDHAP3                .
## PPIHP1                .
## FAHD2P1               .
## AKR7L                 .
## NCAPGP2               .
## RPL7P22               .
## COPS8P2               .
## GPS2P1                .
## CLIC4P3               .
## MIOXP1                .
## TSPY9P                .
## AADACP1               .
## ADAM20P2              .
## MAGOH3P               .
## RPSAP58               .
## FXNP2                 .
## MTND6P25              .
```

```
## PCDHB18P             .
## DDX50P1              .
## PCDHB19P             .
## GTF3AP2              .
## MTCO3P12             .
## HMGB2P1              .
## OR7E62P              .
## LYPLA2P1             .
## OR7E39P              .
## HIGD1AP13            .
## RPS26P49             .
## FABP5P12             .
## REXO1L12P            .
## COL6A4P1             .
## HSPD1P1              .
## HBBP1                .
## RPL13AP20            .
## RPL7P18              .
## KRT18P59             .
## OR4A2P               .
## LAP3P1               .
## PAICSP4              .
## PGAM1P7              .
## DPH3P2               .
## DDX11L5              .
## RPS28P7              .
## HSP90AB3P            .
## UQCRBP2              .
## YWHAZP4              .
## BCAR1P1              .
## BMS1P16              .
## RPS27P12             0.501617531
## CTBP2P2              .
## VDAC1P1              .
## CEACAM22P            .
## ZNF252P              .
## MRPS5P3              .
## PPIAP31              .
## ACTG1P24             .
## BTG1P1               .
## SEC63P1              .
## DHX40P1              .
## LYPLA1P3             .
## XPOTP1               .
## NF1P5                .
## IGFL1P1              .
## CICP26               .
## EGFEM1P              .
## GSTT2                .
## TNPO1P3              .
## EFTUD1P1             .
## MRPL35P3             .
## RAC1P2               .
## CYP3A51P             .
## CYP2D8P              .
## HMGN1P28             .
## HMGN2P15             .
## MST1L                .
## IGKV2_26             .
## SUMO1P3              .
## ZNF887P              .
## KLF17P2              .
## SLC9A3P2             .
## PHB2P1               .
## PHBP12               .
```

```
## BCLAF1P1              .
## SLC25A24P1            .
## HNRNPA1P12            .
## RPL35AP32             .
## HMGN2P5               .
## YAP1P1                .
## ELL2P1                .
## MCTS2P                .
## OR7E15P               .
## MSL3P1                .
## NMNAT1P3              .
## GAPDHP1               .
## RPL10P3               .
## LDHAP4                .
## PCDHGB9P              .
## SEPT14P19             .
## TATDN2P2              .
## HNRNPA1P8             .
## ARL2BPP4              .
## OR52U1P               .
## AKR1C8P               .
## PRR13P5               0.011341937
## RPL7P58               .
## VN1R34P               .
## CALM1P2               .
## TPM3P9                .
## FABP5P11              .
## HSPA9P1               .
## CAP1P2                .
## AMYP1                 .
## IDSP1                 .
## SPDYE11               .
## GCNT1P4               .
## MTMR9LP               .
## ST13P2                .
## SPATA31D5P            .
## HMGN1P15              .
## PSMC1P7               .
## RNFT1P3               .
## RPS3AP54              .
## IGHV3_71              .
## RANP1                 .
## IGLV1_41              .
## RPL12P25              .
## MSX2P1                .
## HMGB1P37              .
## OR14L1P               .
## MRPS17P1              .
## MICC                  .
## RPSAP21               .
## TRIM80P               .
## HSPE1P25              .
## E2F6P1                .
## TATDN1P1              .
## OR2A9P                .
## OR7E111P              .
## IGHV1_12              .
## YWHAZP6               .
## PPIAP13               .
## TMEM14DP              .
## RPL21P13              .
## HNRNPCP3              .
## FAM86B3P              .
## WRBP1                 .
## SNRPCP3               .
```

```
## PFN1P4               .
## PNMA6B               .
## VN1R93P              .
## IGKV2D_23            3.667663388
## GRAMD4P8             .
## NPM1P25              .
## FAM86KP              .
## HLA_V                .
## CYP21A1P             .
## C8orf59P2            .
## RPL7L1P13            .
## RPS12P10             .
## PROS2P               .
## EIF4A1P10            .
## VDAC1P8              .
## NBPF5P               .
## NXNP1                .
## HSPD1P10             .
## KHSRPP1              .
## HCG4B                .
## CCT5P2               .
## LINC00643            .
## PMS2P2               .
## YWHAZP3              .
## MRPL42P2             .
## ANAPC1P1             .
## STRCP1               .
## TRIM60P14            .
## RPS2P24              .
## RPS3P1               .
## NSUN5P2              .
## KRT18P19             .
## MRPL15P1             .
## PYY2                 .
## RPS18P12             .
## GAPDHP65             .
## RAB6C_AS1            .
## RPL12P16             .
## GOLGA2P9             .
## CTAGE10P             .
## PHBP1                .
## MIPEPP3              .
## CYP51A1P2            .
## GEMIN8P4             .
## RPL29P14             .
## WASH2P               .
## RPL7P40              .
## GTF2IRD2P1           .
## RPL34P33             .
## POTEKP               .
## RPS3AP6              .
## RPL21P10             .
## HSP90B2P             .
## ISCA1P1              .
## CCT7P2               .
## SMUG1P1              .
## BCAP31P2             .
## OR2I1P               .
## VN1R21P              .
## RPL13AP5             .
## PABPC1P3             .
## FRG1KP               .
## CTAGE3P              .
## DDX11L16             .
## HNRNPA1P70           .
```

```
## ADORA2BP1              .
## BCLAF1P2               .
## H2AFZP4                .
## PEBP1P2                .
## ZNF204P                .
## HNRNPC*HNRNPCP9        .
## OR4C46*OR4C7P          .
## OR4C13*OR4C7P          .
## CTSF*CTSLP8            .
## VN1R4*VN1R6P           .
## TUT1*EEF1GP4           .
## GOLGA2*GOLGA8VP        .
## UBE2L6*UBE2L2          .
## RPSA*RPSAP39           .
## KRT34*KRT16P2          .
## PSME2*PSME2P2          .
## RPL4*RPL4P1            .
## HNRNPD*HNRNPA1P48      .
## HNRNPA1L2*HNRNPA1P48   .
## PPIB*PPIHP1            .
## GPS2*GPS2P1           -0.053349776
## RPSA*RPSAP58           .
## OR4C46*OR4A2P          .
## OR4C13*OR4A2P          .
## YWHAB*YWHAZP4          .
## RPS27*RPS27P12         .
## ACTC1*ACTG1P24         .
## SEC63*SEC63P1          .
## CYP4F11*CYP3A51P       .
## CYP4B1*CYP3A51P        .
## SLC9A1*SLC9A3P2        .
## HNRNPD*HNRNPA1P12      .
## HNRNPA1L2*HNRNPA1P12   .
## MCTS1*MCTS2P           .
## PCDHGA1*PCDHGB9P       .
## PCDHGA12*PCDHGB9P      .
## PCDHGB1*PCDHGB9P       .
## PCDHGA2*PCDHGB9P       .
## PCDHGA5*PCDHGB9P       .
## PCDHB16*PCDHGB9P       .
## PCDHGA6*PCDHGB9P       .
## PCDHB2*PCDHGB9P        .
## HNRNPD*HNRNPA1P8       .
## HNRNPA1L2*HNRNPA1P8    .
## ARL2BP*ARL2BPP4        .
## TRIM21*OR52U1P         .
## NBEAL1*RPL12P25        .
## RPSA*RPSAP21           .
## YWHAB*YWHAZP6          .
## TMEM14B*TMEM14DP       .
## HNRNPC*HNRNPCP3        .
## ZNF506*VN1R93P         .
## ZNF431*VN1R93P         .
## ZNF429*VN1R93P         .
## ZNF254*VN1R93P         .
## ZNF493*VN1R93P         .
## YWHAB*YWHAZP3          .
## RPS18*RPS18P12         .
## NBEAL1*RPL12P16        .
## KCNH3*GOLGA2P9         .
## KCNH1*GOLGA2P9         .
## MIA2*CTAGE10P          .
## RPL29*RPL29P14         .
## MIA2*CTAGE3P           .
## HNRNPD*HNRNPA1P70      .
```

```
## HNRNPA1L2*HNRNPA1P70  .
## ADORA1*ADORA2BP1      .
## ZFP3*ZNF204P          .
## BCHE*CELP             .
## TBC1D28*TBC1D3P2      .
## CYP2C9*CYP2D8P        .
## CEL*CELP              .
## ZFP3*ZNF252P          .
## CYP2A6*CYP2D8P        .
## TP73*TP73_AS1         .
## ACSL1*TPRXL           .
## SEPT5*SEPT7P9         .
## SEPT3*SEPT7P9         .
## ZBBX*GUCY1B2          .
## CDR2*RRN3P3           .
## KCNH6*GOLGA2P9        .
## CYP2A7*CYP2D8P        .
(active.min <- which(as.matrix(coef.min != 0)))
##  [1]    2    5    6   14   19   23   61   79  134  136  169  223  275  293
## [15]  299  301  317  437  442  446  493  509  861  941 1018 1020 1022 1023
## [29] 1030 1031 1034 1046 1048 1073 1086 1114 1164 1219 1266 1348
(index.min <- coef.min[active.min])
##  [1]  0.495993368  4.702272237  0.136788020  0.219953349 14.373617853
##  [6]  0.032217373  0.035734487  0.159598829  0.047178844  0.002768274
## [11]  0.008372821 -0.008675519 -0.114099946  0.058222957  0.012217098
## [16]  0.146633398 -0.015727118  0.088607253  0.015948551  0.077180043
## [21]  0.369951542  0.071648040  0.119397462  0.016464583  6.917183970
## [26] -0.085305142  0.127259216  1.043166033  1.238712267  1.550053891
## [31]  0.805817560  4.134368532  0.260231002  5.583227212  0.123924507
## [36]  1.226227509  0.501617531  0.011341937  3.667663388 -0.053349776
(sel.lasso <- rownames(coef.min)[active.min]) # Important
##  [1] "SLITRK3"      "GALP"         "OR4C13"       "VN1R4"
##  [5] "LCE3C"        "STXBP5"       "IYD"          "ARID1B"
##  [9] "SMR3A"        "DIP2B"        "LOC100101266" "C19orf66"
## [13] "PARP12"       "EXOC1"        "CEL"          "SLC9A1"
## [17] "CCDC28B"      "PCMT1"        "C10orf131"    "HARS2"
## [21] "CELP"         "C7orf53"      "OR52E6"       "HBS1L"
## [25] "MEMO1P3"      "HLA_K"        "GVINP2"       "RPS10P20"
## [29] "HSPA8P1"      "GAPDHP45"     "ANKRD30BP2"   "RPS20P25"
## [33] "KRT18P62"     "OR7E10P"      "SCML2P1"      "RPL5P28"
## [37] "RPS27P12"     "PRR13P5"      "IGKV2D_23"    "GPS2*GPS2P1"
sel.lasso2 <- c(sel.lasso,unlist(strsplit(sel.lasso[grep("[*]",sel.lasso)],"[*]")))
coxfit.lr <- coxph(S.train ~ SLITRK3 + GALP + OR4C13 + VN1R4 + LCE3C + STXBP5 + IYD +
ARID1B + SMR3A + DIP2B + LOC100101266 + C19orf66 + PARP12 + EXOC1 + CEL + SLC9A1 +
CCDC28B + PCMT1 + C10orf131 + HARS2 + CELP + C7orf53 + OR52E6 + HBS1L + MEMO1P3 +
HLA_K + GVINP2 + RPS10P20 + HSPA8P1 + GAPDHP45 + ANKRD30BP2 + RPS20P25 + KRT18P62 +
OR7E10P + SCML2P1 + RPL5P28 + RPS27P12 + PRR13P5 + IGKV2D_23 + GPS2*GPS2P1 + GPS2 +
GPS2P1, data = as.data.frame(X.train[,sel.lasso2]),model=TRUE)
names(coxfit.lr$coefficients) = sub(".*[]]","",names(coefficients(coxfit.lr)))
#################################
## Test set validation - LASSO
#################################

## risk score
#rs.lasso.train <- predict(cv.lasso, newx=X.train, s="lambda.min")
#rs.lasso.test <- predict(cv.lasso, newx=X.test, s="lambda.min")
rs.lasso.train <- predict(coxfit.lr, newdata=as.data.frame(X.train[,sel.lasso2]))
length(rs.lasso.train)
## [1] 508
rs.lasso.test <- predict(coxfit.lr,
newdata=as.data.frame(X.test[,sel.lasso2]),type="lp",se.fit = FALSE)
length(rs.lasso.test)
## [1] 254
# ## median stratification
good.prog.lasso <- (rs.lasso.test < median(rs.lasso.train))
```

```
(fit.lasso <- survfit(S.test ~ good.prog.lasso))
## Call: survfit(formula = S.test ~ good.prog.lasso)
##
##                          n events median 0.95LCL 0.95UCL
## good.prog.lasso=FALSE 129     27   8.12    6.50      NA
## good.prog.lasso=TRUE  125     18  10.80    9.48      NA
# ## logrank test for validation set
# (logrank.lasso <- survdiff(S.test ~ good.prog.lasso))
(logrank.lasso <- survdiff(S.test ~ rs.lasso.test))
## Call:
## survdiff(formula = S.test ~ rs.lasso.test)
##
##                                    N Observed Expected (O-E)^2/E
## rs.lasso.test=-6.12402260196489    1        0  0.00424  4.24e-03
## rs.lasso.test=-5.46716627911781    1        0  1.21198  1.21e+00
## rs.lasso.test=-4.83919510448891    1        0  0.41838  4.18e-01
## rs.lasso.test=-3.74887959758614    1        0  0.07561  7.56e-02
## rs.lasso.test=-3.74544153925291    1        0  0.31626  3.16e-01
## rs.lasso.test=-3.7289169616567     1        0  0.03332  3.33e-02
## rs.lasso.test=-3.61089826001097    1        1  0.17563  3.87e+00
## rs.lasso.test=-3.26457122207135    1        0  0.00000       NaN
## rs.lasso.test=-3.19972988581549    1        0  0.02191  2.19e-02
## rs.lasso.test=-2.84465255001928    1        0  0.38808  3.88e-01
## rs.lasso.test=-2.83275945586744    1        1  0.33666  1.31e+00
## rs.lasso.test=-2.8189124797241     1        1  0.13973  5.30e+00
## rs.lasso.test=-2.79980257931945    1        0  0.18829  1.88e-01
## rs.lasso.test=-2.78893431065472    1        0  0.03332  3.33e-02
## rs.lasso.test=-2.78121065526638    1        0  0.01286  1.29e-02
## rs.lasso.test=-2.72204586005388    1        0  0.00000       NaN
## rs.lasso.test=-2.70455245010445    1        0  0.41838  4.18e-01
## rs.lasso.test=-2.69650424519594    1        0  0.02191  2.19e-02
## rs.lasso.test=-2.68147916147473    1        0  0.02191  2.19e-02
## rs.lasso.test=-2.63706644225512    1        0  1.21198  1.21e+00
## rs.lasso.test=-2.60872629281989    1        0  0.02191  2.19e-02
## rs.lasso.test=-2.59477911699339    1        0  0.45287  4.53e-01
## rs.lasso.test=-2.58688830968641    1        1  0.36105  1.13e+00
## rs.lasso.test=-2.53890208337209    1        0  0.45287  4.53e-01
## rs.lasso.test=-2.44816454244445    1        0  1.21198  1.21e+00
## rs.lasso.test=-2.41498860716663    1        0  0.33666  3.37e-01
## rs.lasso.test=-2.33848889628507    1        0  0.12850  1.28e-01
## rs.lasso.test=-2.3236851655204     1        0  0.03332  3.33e-02
## rs.lasso.test=-2.27648006624829    1        0  0.09148  9.15e-02
## rs.lasso.test=-2.25378795380075    1        0  0.27847  2.78e-01
## rs.lasso.test=-2.20104990490008    1        0  0.24593  2.46e-01
## rs.lasso.test=-2.1921784822282     1        1  0.06101  1.45e+01
## rs.lasso.test=-2.18901041661297    1        0  0.11774  1.18e-01
## rs.lasso.test=-2.17999400592235    1        0  1.21198  1.21e+00
## rs.lasso.test=-2.07782059019054    1        0  0.00000       NaN
## rs.lasso.test=-2.03521735986549    1        0  0.02743  2.74e-02
## rs.lasso.test=-2.03287973796636    1        0  0.18829  1.88e-01
## rs.lasso.test=-2.02453353313064    1        0  0.11774  1.18e-01
## rs.lasso.test=-1.96816399774497    1        1  0.27847  1.87e+00
## rs.lasso.test=-1.96601450678807    1        0  0.33666  3.37e-01
## rs.lasso.test=-1.95983613324003    1        0  0.01732  1.73e-02
## rs.lasso.test=-1.91466828360414    1        0  0.36105  3.61e-01
## rs.lasso.test=-1.89882666324354    1        0  0.11774  1.18e-01
## rs.lasso.test=-1.83490616236934    1        0  0.03332  3.33e-02
## rs.lasso.test=-1.82897136884423    1        0  0.02743  2.74e-02
## rs.lasso.test=-1.81911467031688    1        0  0.10865  1.09e-01
## rs.lasso.test=-1.80738023156886    1        0  0.02191  2.19e-02
## rs.lasso.test=-1.805422126969      1        0  0.24593  2.46e-01
## rs.lasso.test=-1.79758791362814    1        0  0.02191  2.19e-02
## rs.lasso.test=-1.79415289456978    1        0  0.02191  2.19e-02
## rs.lasso.test=-1.78632710783422    1        1  0.03332  2.80e+01
## rs.lasso.test=-1.69488469857922    1        0  0.66634  6.66e-01
```

```
## rs.lasso.test=-1.6899540389837      1         0  0.00000        NaN
## rs.lasso.test=-1.61345772159328     1         0  0.36105   3.61e-01
## rs.lasso.test=-1.53324925514691     1         0  0.12850   1.28e-01
## rs.lasso.test=-1.52887430131693     1         0  0.45287   4.53e-01
## rs.lasso.test=-1.52796675786591     1         1  0.66634   1.67e-01
## rs.lasso.test=-1.52010462258034     1         0  0.02191   2.19e-02
## rs.lasso.test=-1.51466268398189     1         0  0.11774   1.18e-01
## rs.lasso.test=-1.51272373916643     1         0  0.11774   1.18e-01
## rs.lasso.test=-1.50891949830986     1         0  0.06820   6.82e-02
## rs.lasso.test=-1.50427160862151     1         0  0.18829   1.88e-01
## rs.lasso.test=-1.49638054436997     1         1  0.02191   4.37e+01
## rs.lasso.test=-1.49148744735441     1         0  0.06820   6.82e-02
## rs.lasso.test=-1.48390756112304     1         0  0.03332   3.33e-02
## rs.lasso.test=-1.43742349404024     1         0  0.02191   2.19e-02
## rs.lasso.test=-1.38599084606298     1         0  0.01286   1.29e-02
## rs.lasso.test=-1.36170236580708     1         0  0.07561   7.56e-02
## rs.lasso.test=-1.34216170590409     1         0  0.06820   6.82e-02
## rs.lasso.test=-1.34180055197494     1         0  0.03332   3.33e-02
## rs.lasso.test=-1.27219372991869     1         0  0.38808   3.88e-01
## rs.lasso.test=-1.26052963827282     1         0  0.58942   5.89e-01
## rs.lasso.test=-1.21281087428524     1         0  0.11774   1.18e-01
## rs.lasso.test=-1.19287205313386     1         0  0.02743   2.74e-02
## rs.lasso.test=-1.17451971077493     1         0  0.11774   1.18e-01
## rs.lasso.test=-1.1505540077543      1         0  0.33666   3.37e-01
## rs.lasso.test=-1.13838400250417     1         0  0.07561   7.56e-02
## rs.lasso.test=-1.09621102053717     1         0  0.29665   2.97e-01
## rs.lasso.test=-1.06378249268631     1         0  0.58942   5.89e-01
## rs.lasso.test=-1.04478756130621     1         0  0.11774   1.18e-01
## rs.lasso.test=-1.02738515841251     1         0  0.29665   2.97e-01
## rs.lasso.test=-1.02618614516356     1         1  0.29665   1.67e+00
## rs.lasso.test=-1.01659074836515     1         1  0.06820   1.27e+01
## rs.lasso.test=-1.00497402971856     1         0  0.36105   3.61e-01
## rs.lasso.test=-0.950023607818141    1         0  0.02191   2.19e-02
## rs.lasso.test=-0.93710093697511     1         0  0.03332   3.33e-02
## rs.lasso.test=-0.931295577932625    1         0  0.03332   3.33e-02
## rs.lasso.test=-0.917423846545303    1         0  0.07561   7.56e-02
## rs.lasso.test=-0.895673708771109    1         0  0.01732   1.73e-02
## rs.lasso.test=-0.887954012625259    1         0  0.10865   1.09e-01
## rs.lasso.test=-0.879579117555967    1         0  0.02743   2.74e-02
## rs.lasso.test=-0.854696196124925    1         1  0.21630   2.84e+00
## rs.lasso.test=-0.836180328510047    1         0  0.02191   2.19e-02
## rs.lasso.test=-0.812501852059935    1         0  0.20180   2.02e-01
## rs.lasso.test=-0.795972909797307    1         0  0.00000        NaN
## rs.lasso.test=-0.761540158059333    1         0  0.03332   3.33e-02
## rs.lasso.test=-0.743994400527462    1         0  0.06101   6.10e-02
## rs.lasso.test=-0.71723265874893     1         0  0.33666   3.37e-01
## rs.lasso.test=-0.707578062616094    1         1  0.49635   5.11e-01
## rs.lasso.test=-0.700755159045461    1         0  0.11774   1.18e-01
## rs.lasso.test=-0.677478783719396    1         1  0.04007   2.30e+01
## rs.lasso.test=-0.675204535370398    1         0  0.33666   3.37e-01
## rs.lasso.test=-0.655392763999926    1         0  0.02191   2.19e-02
## rs.lasso.test=-0.629000412822831    1         0  0.20180   2.02e-01
## rs.lasso.test=-0.627078232193139    1         0  0.02743   2.74e-02
## rs.lasso.test=-0.59822239291176     1         0  0.09148   9.15e-02
## rs.lasso.test=-0.57632781700806     1         0  0.00000        NaN
## rs.lasso.test=-0.571690259547789    1         0  0.58942   5.89e-01
## rs.lasso.test=-0.561233459971007    1         0  0.15123   1.51e-01
## rs.lasso.test=-0.484970724199704    1         0  0.02743   2.74e-02
## rs.lasso.test=-0.484122639961451    1         0  0.20180   2.02e-01
## rs.lasso.test=-0.473740204815273    1         0  0.15123   1.51e-01
## rs.lasso.test=-0.471788732117945    1         0  0.02191   2.19e-02
## rs.lasso.test=-0.457420560180601    1         1  0.18829   3.50e+00
## rs.lasso.test=-0.451328456175861    1         1  0.00853   1.15e+02
## rs.lasso.test=-0.437242709662089    1         0  0.02191   2.19e-02
## rs.lasso.test=-0.433566511379887    1         1  1.04531   1.96e-03
```

```
## rs.lasso.test=-0.400758567931095    1         0  0.13973  1.40e-01
## rs.lasso.test=-0.395574260900808    1         0  0.02743  2.74e-02
## rs.lasso.test=-0.377348162736531    1         0  0.02191  2.19e-02
## rs.lasso.test=-0.350775193677233    1         0  0.29665  2.97e-01
## rs.lasso.test=-0.345183226093772    1         1  0.08348  1.01e+01
## rs.lasso.test=-0.341061691578058    1         0  0.07561  7.56e-02
## rs.lasso.test=-0.333927137943877    1         0  0.09148  9.15e-02
## rs.lasso.test=-0.331470334432372    1         0  0.02191  2.19e-02
## rs.lasso.test=-0.317980729923466    1         0  0.03332  3.33e-02
## rs.lasso.test=-0.311744255392832    1         0  0.24593  2.46e-01
## rs.lasso.test=-0.292180737268487    1         0  0.07561  7.56e-02
## rs.lasso.test=-0.261720436321077    1         0  0.58942  5.89e-01
## rs.lasso.test=-0.256146165875599    1         0  0.02743  2.74e-02
## rs.lasso.test=-0.234121350100484    1         1  0.00424  2.34e+02
## rs.lasso.test=-0.231889819334631    1         0  0.00000       NaN
## rs.lasso.test=-0.222077783406051    1         0  0.27847  2.78e-01
## rs.lasso.test=-0.196746891927224    1         0  0.04007  4.01e-02
## rs.lasso.test=-0.179048447233887    1         0  0.02191  2.19e-02
## rs.lasso.test=-0.154909400745275    1         0  0.10865  1.09e-01
## rs.lasso.test=-0.142021465038968    1         0  0.02191  2.19e-02
## rs.lasso.test=-0.115729943622165    1         0  0.00000       NaN
## rs.lasso.test=-0.113305790372047    1         0  0.41838  4.18e-01
## rs.lasso.test=-0.104556349843264    1         1  0.10865  7.31e+00
## rs.lasso.test=-0.0888351831684131   1         0  0.16313  1.63e-01
## rs.lasso.test=-0.0596098589699525   1         0  0.66634  6.66e-01
## rs.lasso.test=-0.049335648074373    1         1  0.31626  1.48e+00
## rs.lasso.test=-0.0425983009497223   1         0  0.05391  5.39e-02
## rs.lasso.test=-0.0221823558581078   1         0  0.02191  2.19e-02
## rs.lasso.test=-0.000298171548923198 1         0  0.08348  8.35e-02
## rs.lasso.test=0.013730158217093     1         0  0.09996  1.00e-01
## rs.lasso.test=0.0173750918706579    1         0  0.01286  1.29e-02
## rs.lasso.test=0.0190651272724487    1         0  0.11774  1.18e-01
## rs.lasso.test=0.033540812029259     1         1  0.77745  6.37e-02
## rs.lasso.test=0.0370051853548509    1         1  0.02743  3.45e+01
## rs.lasso.test=0.0381588014885739    1         0  0.09148  9.15e-02
## rs.lasso.test=0.0409219660250423    1         0  0.27847  2.78e-01
## rs.lasso.test=0.050449743734291     1         1  0.90245  1.05e-02
## rs.lasso.test=0.0544768664398738    1         0  0.03332  3.33e-02
## rs.lasso.test=0.0972506532535435    1         1  0.20180  3.16e+00
## rs.lasso.test=0.103756755313463     1         0  0.02191  2.19e-02
## rs.lasso.test=0.133725472306962     1         0  0.12850  1.28e-01
## rs.lasso.test=0.149060398081627     1         0  0.38808  3.88e-01
## rs.lasso.test=0.17044335613947      1         0  0.03332  3.33e-02
## rs.lasso.test=0.181807420027072     1         1  0.58942  2.86e-01
## rs.lasso.test=0.186458632458985     1         0  0.00853  8.53e-03
## rs.lasso.test=0.203197375361683     1         0  0.01732  1.73e-02
## rs.lasso.test=0.243096064198087     1         0  0.01732  1.73e-02
## rs.lasso.test=0.254630056500998     1         0  0.02191  2.19e-02
## rs.lasso.test=0.291654560245775     1         0  0.10865  1.09e-01
## rs.lasso.test=0.317948208961944     1         1  0.38808  9.65e-01
## rs.lasso.test=0.376407001030947     1         0  0.18829  1.88e-01
## rs.lasso.test=0.416995194556076     1         0  0.00000       NaN
## rs.lasso.test=0.430447320734851     1         0  0.00000       NaN
## rs.lasso.test=0.466136315245214     1         0  0.09148  9.15e-02
## rs.lasso.test=0.47506657415772      1         0  0.02191  2.19e-02
## rs.lasso.test=0.549653706186958     1         0  0.45287  4.53e-01
## rs.lasso.test=0.571994502895092     1         0  1.21198  1.21e+00
## rs.lasso.test=0.581833771723988     1         0  0.03332  3.33e-02
## rs.lasso.test=0.58452510836766      1         0  0.02191  2.19e-02
## rs.lasso.test=0.594684421149936     1         0  0.00000       NaN
## rs.lasso.test=0.620910853481949     1         0  0.11774  1.18e-01
## rs.lasso.test=0.630406645062983     1         0  0.02191  2.19e-02
## rs.lasso.test=0.650856469615407     1         1  0.24593  2.31e+00
## rs.lasso.test=0.665553215275369     1         0  0.09148  9.15e-02
## rs.lasso.test=0.682330421512087     1         0  0.02191  2.19e-02
```

```
## rs.lasso.test=0.686163333222483      1         0  0.03332  3.33e-02
## rs.lasso.test=0.689086529555492      1         0  0.02191  2.19e-02
## rs.lasso.test=0.691277299345936      1         0  0.01732  1.73e-02
## rs.lasso.test=0.697837851483944      1         0  0.00000       NaN
## rs.lasso.test=0.699079680286222      1         0  0.11774  1.18e-01
## rs.lasso.test=0.723736390177885      1         1  0.41838  8.09e-01
## rs.lasso.test=0.778095125674971      1         1  0.12850  5.91e+00
## rs.lasso.test=0.786737414593388      1         1  0.05391  1.66e+01
## rs.lasso.test=0.812129025794797      1         1  0.09996  8.10e+00
## rs.lasso.test=0.836476135463988      1         1  0.16313  4.29e+00
## rs.lasso.test=0.85017694318434       1         0  0.01286  1.29e-02
## rs.lasso.test=0.903801153499743      1         0  0.02191  2.19e-02
## rs.lasso.test=0.935984969051246      1         0  0.58942  5.89e-01
## rs.lasso.test=0.950006360063067      1         0  0.33666  3.37e-01
## rs.lasso.test=0.953335329133269      1         0  0.11774  1.18e-01
## rs.lasso.test=0.954565475557581      1         0  0.02191  2.19e-02
## rs.lasso.test=0.988173198473115      1         0  0.16313  1.63e-01
## rs.lasso.test=0.991140669965575      1         0  0.03332  3.33e-02
## rs.lasso.test=1.03431132594138       1         0  0.11774  1.18e-01
## rs.lasso.test=1.05083114121455       1         1  0.01286  7.58e+01
## rs.lasso.test=1.0545799928591        1         1  1.21198  3.71e-02
## rs.lasso.test=1.05574734199237       1         0  0.02191  2.19e-02
## rs.lasso.test=1.13733699721333       1         0  0.03332  3.33e-02
## rs.lasso.test=1.1431022464824        1         0  0.00000       NaN
## rs.lasso.test=1.20721927451123       1         1  0.23100  2.56e+00
## rs.lasso.test=1.21430407395956       1         0  0.58942  5.89e-01
## rs.lasso.test=1.25982933812105       1         0  0.02191  2.19e-02
## rs.lasso.test=1.26652661932484       1         0  0.33666  3.37e-01
## rs.lasso.test=1.29037192971227       1         1  0.26180  2.08e+00
## rs.lasso.test=1.31477745525373       1         0  0.01286  1.29e-02
## rs.lasso.test=1.40655071870525       1         0  0.05391  5.39e-02
## rs.lasso.test=1.41024777397148       1         1  0.09148  9.02e+00
## rs.lasso.test=1.44280244687262       1         0  0.02191  2.19e-02
## rs.lasso.test=1.47143051695752       1         0  0.00000       NaN
## rs.lasso.test=1.5113666346068        1         0  0.26180  2.62e-01
## rs.lasso.test=1.51498681601518       1         0  0.02191  2.19e-02
## rs.lasso.test=1.52553124633765       1         0  0.45287  4.53e-01
## rs.lasso.test=1.63744648568716       1         0  0.20180  2.02e-01
## rs.lasso.test=1.74556971943259       1         0  0.27847  2.78e-01
## rs.lasso.test=1.81750910803497       1         0  0.02191  2.19e-02
## rs.lasso.test=1.94822769922688       1         0  0.16313  1.63e-01
## rs.lasso.test=1.95426256441248       1         1  0.15123  4.76e+00
## rs.lasso.test=1.99725359122321       1         0  0.03332  3.33e-02
## rs.lasso.test=2.03778505585287       1         1  0.45287  6.61e-01
## rs.lasso.test=2.03975658439491       1         0  0.02743  2.74e-02
## rs.lasso.test=2.14790588562938       1         0  0.26180  2.62e-01
## rs.lasso.test=2.21668523244327       1         0  0.00000       NaN
## rs.lasso.test=2.22043207958528       1         0  0.03332  3.33e-02
## rs.lasso.test=2.24195790867168       1         0  0.02743  2.74e-02
## rs.lasso.test=2.44569441985193       1         1  0.54180  3.87e-01
## rs.lasso.test=2.47026689518103       1         0  0.09996  1.00e-01
## rs.lasso.test=2.55505805187712       1         0  0.11774  1.18e-01
## rs.lasso.test=2.60059879253661       1         0  0.66634  6.66e-01
## rs.lasso.test=2.60778394835568       1         0  0.04007  4.01e-02
## rs.lasso.test=2.69218696593783       1         0  0.02191  2.19e-02
## rs.lasso.test=2.69979088354329       1         0  0.03332  3.33e-02
## rs.lasso.test=2.95228973206286       1         0  0.11774  1.18e-01
## rs.lasso.test=3.00791767585216       1         1  0.07561  1.13e+01
## rs.lasso.test=3.0467138729476        1         0  0.00424  4.24e-03
## rs.lasso.test=3.06818527463301       1         0  0.02191  2.19e-02
## rs.lasso.test=3.12465840383428       1         1  0.04697  1.93e+01
## rs.lasso.test=3.46043678120017       1         0  0.03332  3.33e-02
## rs.lasso.test=3.46191348458255       1         0  0.58942  5.89e-01
## rs.lasso.test=3.75529436499705       1         0  0.01286  1.29e-02
## rs.lasso.test=3.95671088839248       1         1  0.01732  5.57e+01
```

```
## rs.lasso.test=3.96032538631438    1        0  0.00000       NaN
## rs.lasso.test=4.81718535927043    1        0  0.02191  2.19e-02
## rs.lasso.test=5.66334319514631    1        0  0.00000       NaN
## rs.lasso.test=6.21540452777068    1        1  0.11774  6.61e+00
## rs.lasso.test=6.95727595499559    1        0  0.00000       NaN
## rs.lasso.test=8.43135151689843    1        0  0.03332  3.33e-02
## rs.lasso.test=15.2122770559427    1        0  0.07561  7.56e-02
##                                  (O-E)^2/V
## rs.lasso.test=-6.12402260196489    4.26e-03
## rs.lasso.test=-5.46716627911781    1.32e+00
## rs.lasso.test=-4.83919510448891    4.25e-01
## rs.lasso.test=-3.74887959758614    7.61e-02
## rs.lasso.test=-3.74544153925291    3.20e-01
## rs.lasso.test=-3.7289169616567     3.35e-02
## rs.lasso.test=-3.61089826001097    3.90e+00
## rs.lasso.test=-3.26457122207135         NaN
## rs.lasso.test=-3.19972988581549    2.20e-02
## rs.lasso.test=-2.84465255001928    3.94e-01
## rs.lasso.test=-2.83275945586744    1.32e+00
## rs.lasso.test=-2.8189124797241     5.34e+00
## rs.lasso.test=-2.79980257931945    1.90e-01
## rs.lasso.test=-2.78893431065472    3.35e-02
## rs.lasso.test=-2.78121065526638    1.29e-02
## rs.lasso.test=-2.72204586005388         NaN
## rs.lasso.test=-2.70455245010445    4.25e-01
## rs.lasso.test=-2.69650424519594    2.20e-02
## rs.lasso.test=-2.68147916147473    2.20e-02
## rs.lasso.test=-2.63706644225512    1.32e+00
## rs.lasso.test=-2.60872629281989    2.20e-02
## rs.lasso.test=-2.59477911699339    4.61e-01
## rs.lasso.test=-2.58688830968641    1.15e+00
## rs.lasso.test=-2.53890208337209    4.61e-01
## rs.lasso.test=-2.44816454244445    1.32e+00
## rs.lasso.test=-2.41498860716663    3.41e-01
## rs.lasso.test=-2.33848889628507    1.29e-01
## rs.lasso.test=-2.3236851655204     3.35e-02
## rs.lasso.test=-2.27648006624829    9.21e-02
## rs.lasso.test=-2.25378795380075    2.82e-01
## rs.lasso.test=-2.20104990490008    2.48e-01
## rs.lasso.test=-2.1921784822282     1.45e+01
## rs.lasso.test=-2.18901041661297    1.19e-01
## rs.lasso.test=-2.17999400592235    1.32e+00
## rs.lasso.test=-2.07782059019054         NaN
## rs.lasso.test=-2.03521735986549    2.76e-02
## rs.lasso.test=-2.03287973796636    1.90e-01
## rs.lasso.test=-2.02453353313064    1.19e-01
## rs.lasso.test=-1.96816399774497    1.89e+00
## rs.lasso.test=-1.96601450678807    3.41e-01
## rs.lasso.test=-1.95983613324003    1.74e-02
## rs.lasso.test=-1.91466828360414    3.66e-01
## rs.lasso.test=-1.89882666324354    1.19e-01
## rs.lasso.test=-1.83490616236934    3.35e-02
## rs.lasso.test=-1.82897136884423    2.76e-02
## rs.lasso.test=-1.81911467031688    1.09e-01
## rs.lasso.test=-1.80738023156886    2.20e-02
## rs.lasso.test=-1.805422126969      2.48e-01
## rs.lasso.test=-1.79758791362814    2.20e-02
## rs.lasso.test=-1.79415289456978    2.20e-02
## rs.lasso.test=-1.78632710783422    2.82e+01
## rs.lasso.test=-1.69488469857922    6.87e-01
## rs.lasso.test=-1.6899540389837          NaN
## rs.lasso.test=-1.61345772159328    3.66e-01
## rs.lasso.test=-1.53324925514691    1.29e-01
## rs.lasso.test=-1.52887430131693    4.61e-01
## rs.lasso.test=-1.52796675786591    1.72e-01
```

```
## rs.lasso.test=-1.52010462258034      2.20e-02
## rs.lasso.test=-1.51466268398189      1.19e-01
## rs.lasso.test=-1.51272373916643      1.19e-01
## rs.lasso.test=-1.50891949830986      6.86e-02
## rs.lasso.test=-1.50427160862151      1.90e-01
## rs.lasso.test=-1.49638054436997      4.39e+01
## rs.lasso.test=-1.49148744735441      6.86e-02
## rs.lasso.test=-1.48390756112304      3.35e-02
## rs.lasso.test=-1.43742349404024      2.20e-02
## rs.lasso.test=-1.38599084606298      1.29e-02
## rs.lasso.test=-1.36170236580708      7.61e-02
## rs.lasso.test=-1.34216170590409      6.86e-02
## rs.lasso.test=-1.34180055197494      3.35e-02
## rs.lasso.test=-1.27219372991869      3.94e-01
## rs.lasso.test=-1.26052963827282      6.04e-01
## rs.lasso.test=-1.21281087428524      1.19e-01
## rs.lasso.test=-1.19287205313386      2.76e-02
## rs.lasso.test=-1.17451971077493      1.19e-01
## rs.lasso.test=-1.1505540077543       3.41e-01
## rs.lasso.test=-1.13838400250417      7.61e-02
## rs.lasso.test=-1.09621102053717      3.00e-01
## rs.lasso.test=-1.06378249268631      6.04e-01
## rs.lasso.test=-1.04478756130621      1.19e-01
## rs.lasso.test=-1.02738515841251      3.00e-01
## rs.lasso.test=-1.02618614516356      1.69e+00
## rs.lasso.test=-1.01659074836515      1.28e+01
## rs.lasso.test=-1.00497402971856      3.66e-01
## rs.lasso.test=-0.950023607818141     2.20e-02
## rs.lasso.test=-0.93710093697511      3.35e-02
## rs.lasso.test=-0.931295577932625     3.35e-02
## rs.lasso.test=-0.917423846545303     7.61e-02
## rs.lasso.test=-0.895673708771109     1.74e-02
## rs.lasso.test=-0.887954012625259     1.09e-01
## rs.lasso.test=-0.879579117555967     2.76e-02
## rs.lasso.test=-0.854696196124925     2.87e+00
## rs.lasso.test=-0.836180328510047     2.20e-02
## rs.lasso.test=-0.812501852059935     2.04e-01
## rs.lasso.test=-0.795972909797307          NaN
## rs.lasso.test=-0.761540158059333     3.35e-02
## rs.lasso.test=-0.743994400527462     6.14e-02
## rs.lasso.test=-0.71723265874893      3.41e-01
## rs.lasso.test=-0.707578062616094     5.21e-01
## rs.lasso.test=-0.700755159045461     1.19e-01
## rs.lasso.test=-0.677478783719396     2.31e+01
## rs.lasso.test=-0.675204535370398     3.41e-01
## rs.lasso.test=-0.655392763999926     2.20e-02
## rs.lasso.test=-0.629000412822831     2.04e-01
## rs.lasso.test=-0.627078232193139     2.76e-02
## rs.lasso.test=-0.59822239291176      9.21e-02
## rs.lasso.test=-0.57632781700806           NaN
## rs.lasso.test=-0.571690259547789     6.04e-01
## rs.lasso.test=-0.561233459971007     1.52e-01
## rs.lasso.test=-0.484970724199704     2.76e-02
## rs.lasso.test=-0.484122639961451     2.04e-01
## rs.lasso.test=-0.473740204815273     1.52e-01
## rs.lasso.test=-0.471788732117945     2.20e-02
## rs.lasso.test=-0.457420560180601     3.53e+00
## rs.lasso.test=-0.451328456175861     1.16e+02
## rs.lasso.test=-0.437242709662089     2.20e-02
## rs.lasso.test=-0.433566511379887     2.10e-03
## rs.lasso.test=-0.400758567931095     1.41e-01
## rs.lasso.test=-0.395574260900808     2.76e-02
## rs.lasso.test=-0.377348162736531     2.20e-02
## rs.lasso.test=-0.350775193677233     3.00e-01
## rs.lasso.test=-0.345183226093772     1.01e+01
```

```
## rs.lasso.test=-0.341061691578058     7.61e-02
## rs.lasso.test=-0.333927137943877     9.21e-02
## rs.lasso.test=-0.331470334432372     2.20e-02
## rs.lasso.test=-0.317980729923466     3.35e-02
## rs.lasso.test=-0.311744255392832     2.48e-01
## rs.lasso.test=-0.292180737268487     7.61e-02
## rs.lasso.test=-0.261720436321077     6.04e-01
## rs.lasso.test=-0.256146165875599     2.76e-02
## rs.lasso.test=-0.234121350100484     2.35e+02
## rs.lasso.test=-0.231889819334631          NaN
## rs.lasso.test=-0.222077783406051     2.82e-01
## rs.lasso.test=-0.196746891927224     4.03e-02
## rs.lasso.test=-0.179048447233887     2.20e-02
## rs.lasso.test=-0.154909400745275     1.09e-01
## rs.lasso.test=-0.142021465038968     2.20e-02
## rs.lasso.test=-0.115729943622165          NaN
## rs.lasso.test=-0.113305790372047     4.25e-01
## rs.lasso.test=-0.104556349843264     7.36e+00
## rs.lasso.test=-0.0888351831684131    1.64e-01
## rs.lasso.test=-0.0596098589699525    6.87e-01
## rs.lasso.test=-0.049335648074373     1.50e+00
## rs.lasso.test=-0.0425983009497223    5.42e-02
## rs.lasso.test=-0.0221823558581078    2.20e-02
## rs.lasso.test=-0.000298171548923198  8.40e-02
## rs.lasso.test=0.013730158217093      1.01e-01
## rs.lasso.test=0.0173750918706579     1.29e-02
## rs.lasso.test=0.0190651272724487     1.19e-01
## rs.lasso.test=0.033540812029259      6.64e-02
## rs.lasso.test=0.0370051853548509     3.46e+01
## rs.lasso.test=0.0381588014885739     9.21e-02
## rs.lasso.test=0.0409219660250423     2.82e-01
## rs.lasso.test=0.050449743734291      1.11e-02
## rs.lasso.test=0.0544768664398738     3.35e-02
## rs.lasso.test=0.0972506532535435     3.19e+00
## rs.lasso.test=0.103756755313463      2.20e-02
## rs.lasso.test=0.133725472306962      1.29e-01
## rs.lasso.test=0.149060398081627      3.94e-01
## rs.lasso.test=0.17044335613947       3.35e-02
## rs.lasso.test=0.181807420027072      2.93e-01
## rs.lasso.test=0.186458632458985      8.57e-03
## rs.lasso.test=0.203197375361683      1.74e-02
## rs.lasso.test=0.243096064198087      1.74e-02
## rs.lasso.test=0.254630056500998      2.20e-02
## rs.lasso.test=0.291654560245775      1.09e-01
## rs.lasso.test=0.317948208961944      9.79e-01
## rs.lasso.test=0.376407001030947      1.90e-01
## rs.lasso.test=0.416995194556076           NaN
## rs.lasso.test=0.430447320734851           NaN
## rs.lasso.test=0.466136315245214      9.21e-02
## rs.lasso.test=0.47506657415772       2.20e-02
## rs.lasso.test=0.549653706186958      4.61e-01
## rs.lasso.test=0.571994502895092      1.32e+00
## rs.lasso.test=0.581833771723988      3.35e-02
## rs.lasso.test=0.58452510836766       2.20e-02
## rs.lasso.test=0.594684421149936           NaN
## rs.lasso.test=0.620910853481949      1.19e-01
## rs.lasso.test=0.630406645062983      2.20e-02
## rs.lasso.test=0.650856469615407      2.34e+00
## rs.lasso.test=0.665553215275369      9.21e-02
## rs.lasso.test=0.682330421512087      2.20e-02
## rs.lasso.test=0.686163333222483      3.35e-02
## rs.lasso.test=0.689086529555492      2.20e-02
## rs.lasso.test=0.691277299345936      1.74e-02
## rs.lasso.test=0.697837851483944           NaN
## rs.lasso.test=0.699079680286222      1.19e-01
```

```
## rs.lasso.test=0.723736390177885      8.21e-01
## rs.lasso.test=0.778095125674971      5.95e+00
## rs.lasso.test=0.786737414593388      1.67e+01
## rs.lasso.test=0.812129025794797      8.16e+00
## rs.lasso.test=0.836476135463988      4.33e+00
## rs.lasso.test=0.85017694318434       1.29e-02
## rs.lasso.test=0.903801153499743      2.20e-02
## rs.lasso.test=0.935984969051246      6.04e-01
## rs.lasso.test=0.950006360063067      3.41e-01
## rs.lasso.test=0.953335329133269      1.19e-01
## rs.lasso.test=0.954565475557581      2.20e-02
## rs.lasso.test=0.988173198473115      1.64e-01
## rs.lasso.test=0.991140669965575      3.35e-02
## rs.lasso.test=1.03431132594138       1.19e-01
## rs.lasso.test=1.05083114121455       7.61e+01
## rs.lasso.test=1.0545799928591        4.03e-02
## rs.lasso.test=1.05574734199237       2.20e-02
## rs.lasso.test=1.13733699721333       3.35e-02
## rs.lasso.test=1.1431022464824             NaN
## rs.lasso.test=1.20721927451123       2.59e+00
## rs.lasso.test=1.21430407395956       6.04e-01
## rs.lasso.test=1.25982933812105       2.20e-02
## rs.lasso.test=1.26652661932484       3.41e-01
## rs.lasso.test=1.29037192971227       2.10e+00
## rs.lasso.test=1.31477745525373       1.29e-02
## rs.lasso.test=1.40655071870525       5.42e-02
## rs.lasso.test=1.41024777397148       9.08e+00
## rs.lasso.test=1.44280244687262       2.20e-02
## rs.lasso.test=1.47143051695752            NaN
## rs.lasso.test=1.5113666346068        2.65e-01
## rs.lasso.test=1.51498681601518       2.20e-02
## rs.lasso.test=1.52553124633765       4.61e-01
## rs.lasso.test=1.63744648568716       2.04e-01
## rs.lasso.test=1.74556971943259       2.82e-01
## rs.lasso.test=1.81750910803497       2.20e-02
## rs.lasso.test=1.94822769922688       1.64e-01
## rs.lasso.test=1.95426256441248       4.80e+00
## rs.lasso.test=1.99725359122321       3.35e-02
## rs.lasso.test=2.03778505585287       6.72e-01
## rs.lasso.test=2.03975658439491       2.76e-02
## rs.lasso.test=2.14790588562938       2.65e-01
## rs.lasso.test=2.21668523244327            NaN
## rs.lasso.test=2.22043207958528       3.35e-02
## rs.lasso.test=2.24195790867168       2.76e-02
## rs.lasso.test=2.44569441985193       3.96e-01
## rs.lasso.test=2.47026689518103       1.01e-01
## rs.lasso.test=2.55505805187712       1.19e-01
## rs.lasso.test=2.60059879253661       6.87e-01
## rs.lasso.test=2.60778394835568       4.03e-02
## rs.lasso.test=2.69218696593783       2.20e-02
## rs.lasso.test=2.69979088354329       3.35e-02
## rs.lasso.test=2.95228973206286       1.19e-01
## rs.lasso.test=3.00791767585216       1.14e+01
## rs.lasso.test=3.0467138729476        4.26e-03
## rs.lasso.test=3.06818527463301       2.20e-02
## rs.lasso.test=3.12465840383428       1.94e+01
## rs.lasso.test=3.46043678120017       3.35e-02
## rs.lasso.test=3.46191348458255       6.04e-01
## rs.lasso.test=3.75529436499705       1.29e-02
## rs.lasso.test=3.95671088839248       5.60e+01
## rs.lasso.test=3.96032538631438            NaN
## rs.lasso.test=4.81718535927043       2.20e-02
## rs.lasso.test=5.66334319514631            NaN
## rs.lasso.test=6.21540452777068       6.66e+00
## rs.lasso.test=6.95727595499559            NaN
```

```
## rs.lasso.test=8.43135151689843       3.35e-02
## rs.lasso.test=15.2122770559427       7.61e-02
##
##  Chisq= 849  on 235 degrees of freedom, p= <2e-16
(p.median.lasso <- 1-pchisq(logrank.lasso$chisq, 1))
## [1] 0
## cox regression on risk score
#cox.lasso <- coxph(S.test ~ good.prog.lasso)
cox.lasso <- coxph(S.test ~ rs.lasso.test)
summary(cox.lasso)
## Call:
## coxph(formula = S.test ~ rs.lasso.test)
##
##   n= 254, number of events= 45
##
##                   coef exp(coef) se(coef)     z Pr(>|z|)
## rs.lasso.test 0.15374   1.16619  0.05687 2.703  0.00687 **
## ---
## Signif. codes:  0 '***' 0.001 '**' 0.01 '*' 0.05 '.' 0.1 ' ' 1
##
##               exp(coef) exp(-coef) lower .95 upper .95
## rs.lasso.test     1.166     0.8575     1.043     1.304
##
## Concordance= 0.601  (se = 0.05 )
## Likelihood ratio test= 5.6  on 1 df,   p=0.02
## Wald test            = 7.31  on 1 df,   p=0.007
## Score (logrank) test = 6.59  on 1 df,   p=0.01
## C-index
## C-index assessment - for two selected individuals, the probability that the one
with the higher score (shorter survival) will actually have the shorter survival time.
Good biomarkers have indexes between 0.7 - 0.8.
#(c.lasso <- rcorrcens(S.test ~ good.prog.lasso)[,"C"])
(c.lasso <- rcorrcens(S.test ~ rs.lasso.test)[,"C"]) # 0.3641975
## [1] 0.3988604
## kaplan-meier curves
plot(fit.lasso, lwd = 2, lty = c(1,1), col = c("red","blue"), xlab = 'Time (years)',
     ylab = 'Estimated Survival Function')
legend("topright", legend=c('high risk', 'low risk'), lty = c(1,1),
       col = c("red", "blue"), lwd = 2, bty = "n")
text(6,0.9,paste("p =",round(p.median.lasso,3)))
text(6,0.85,paste("C =",round(c.lasso,3)))
title("Kaplan-Meier Curves, Lasso w/screen")
```

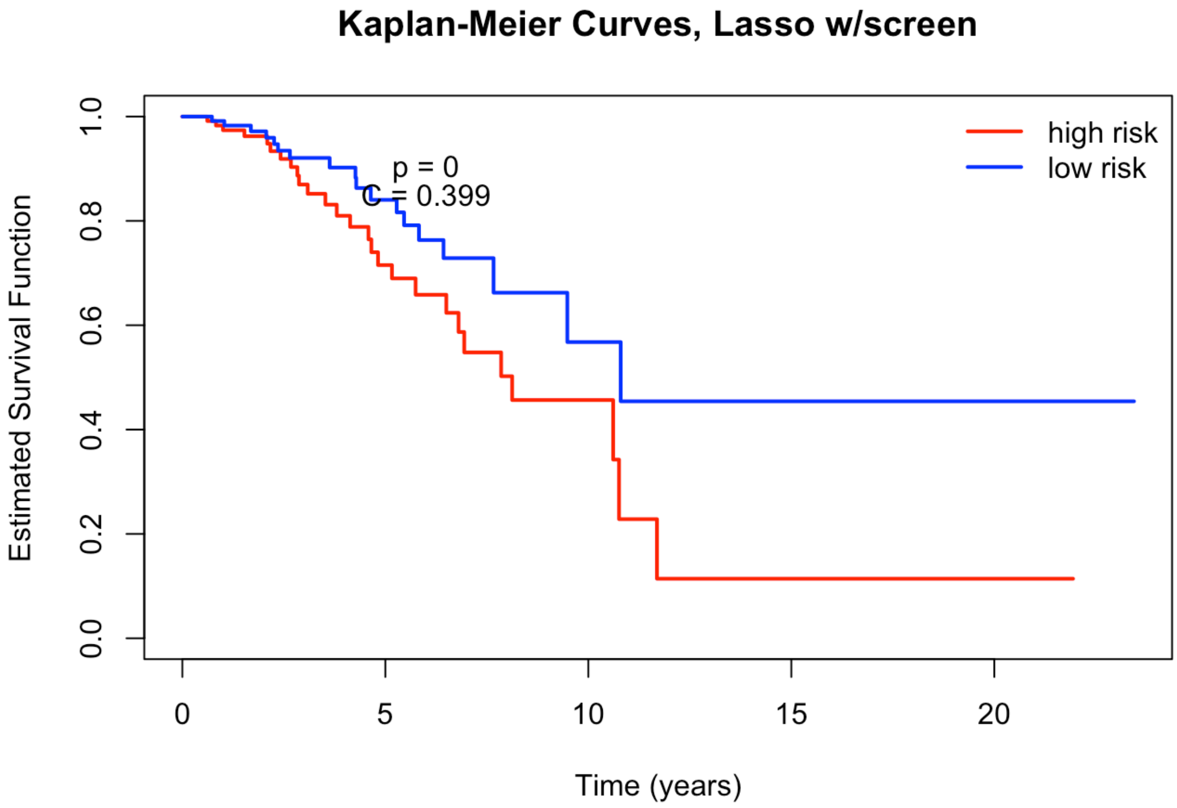


```
########################################################
## YZ newly added code for ctree - YZ 20180628
########################################################

#install.packages("partykit")
#install.packages("coin")
library("partykit")
library("coin")

colnames(RNA.g.pg.counts.01) = sub("-","_",colnames(RNA.g.pg.counts.01))

##Convert all data frame character columns to factors
temp <- surdata4
temp$pathologic_stage <- substring(surdata4$pathologic_stage, 7)
surdata5 <- as.data.frame(unclass(temp[, -1]))
rm(temp)

# BRCA_ctree1 <- ctree(Surv(survival.years, vital_status) ~ pathologic_stage +
pathology_T_stage + pathology_N_stage + pathology_M_stage
#                       + gender + radiation_therapy + histological_type +
number_of_lymph_nodes
#                       + race + ethnicity, data = surdata4)
# plot(BRCA_ctree1)

## Consider expression only
BRCA_ctree2 <- ctree(Surv(survival.years, vital_status) ~ .,
                     data = data.frame(survival.years=surdata5$survival.years,
vital_status=surdata5$vital_status, RNA.g.pg.counts.01[, sel.lasso]))
plot(BRCA_ctree2)
```

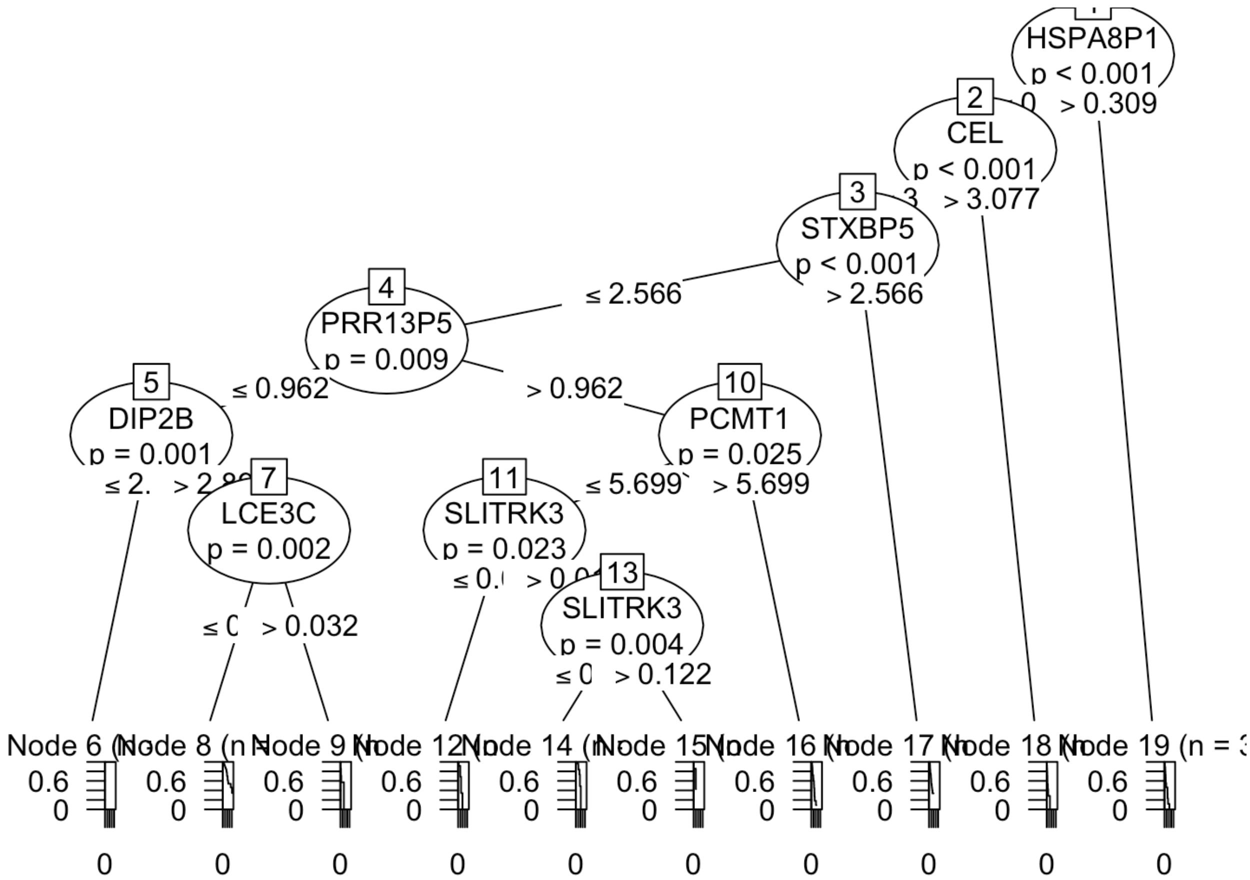


```
## Consider expression and clinical variables
BRCA_ctree3 <- ctree(Surv(survival.years, vital_status) ~ .,
                     data = data.frame(survival.years=surdata5$survival.years,
vital_status=surdata5$vital_status,
                                       pathologic_stage=surdata5$pathologic_stage,
gender=surdata5$gender,
                                       radiation_therapy=surdata5$radiation_therapy,
histological_type=surdata5$histological_type,
                                       race=surdata5$race,
ethnicity=surdata5$ethnicity,
                                       RNA.g.pg.counts.01[, sel.lasso2]))
plot(BRCA_ctree3)
```

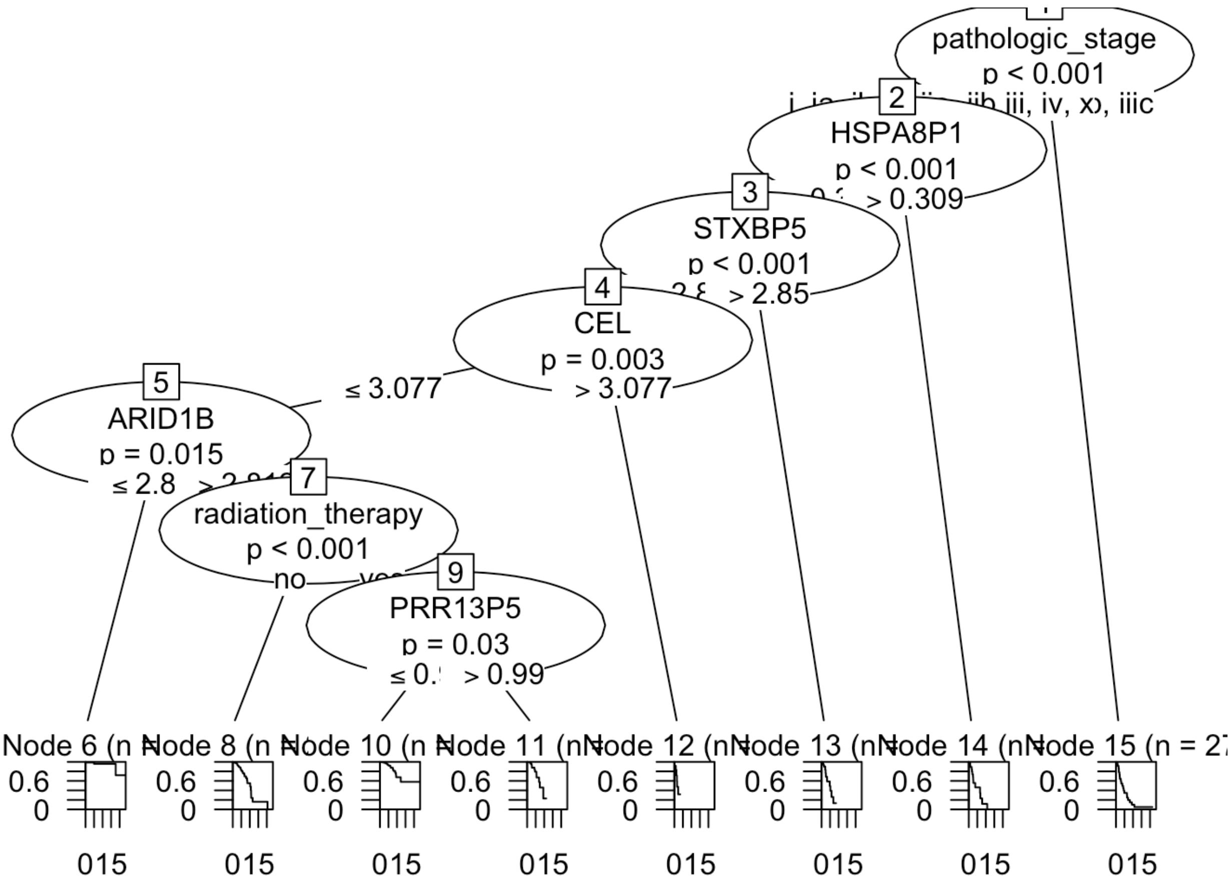


```
####################################################
# Plotting Interaction terms
####################################################

## Split at median expression of GPS2-GPS2P1 interaction
cox.GPS2GPS2P1 <- coxph(Surv(survival.years, vital_status) ~
RNA.g.pg.counts.01[,"GPS2*GPS2P1"], data = surdata4)
summary(cox.GPS2GPS2P1)
## Call:
## coxph(formula = Surv(survival.years, vital_status) ~ RNA.g.pg.counts.01[,
##     "GPS2*GPS2P1"], data = surdata4)
##
##   n= 762, number of events= 118
##
##                                        coef exp(coef) se(coef)      z
## RNA.g.pg.counts.01[, "GPS2*GPS2P1"] -0.23380   0.79152  0.07511 -3.113
##                                    Pr(>|z|)
## RNA.g.pg.counts.01[, "GPS2*GPS2P1"]  0.00185 **
## ---
## Signif. codes:  0 '***' 0.001 '**' 0.01 '*' 0.05 '.' 0.1 ' ' 1
##
##                                    exp(coef) exp(-coef) lower .95
## RNA.g.pg.counts.01[, "GPS2*GPS2P1"]    0.7915      1.263    0.6832
##                                    upper .95
## RNA.g.pg.counts.01[, "GPS2*GPS2P1"]     0.917
##
## Concordance= 0.577  (se = 0.032 )
## Likelihood ratio test= 10.21  on 1 df,   p=0.001
## Wald test            = 9.69  on 1 df,   p=0.002
## Score (logrank) test = 9.86  on 1 df,   p=0.002
```

```
GPS2GPS2P1.grps <- ifelse(RNA.g.pg.counts.01[,"GPS2*GPS2P1"] >
median(RNA.g.pg.counts.01[,"GPS2*GPS2P1"]), 1, 0)
table(GPS2GPS2P1.grps)
## GPS2GPS2P1.grps
##   0   1
## 381 381
km.GPS2GPS2P1 <- survfit(Surv(survival.years, vital_status) ~ GPS2GPS2P1.grps, data =
surdata4)
plot(km.GPS2GPS2P1, col = 1:2, xlab = "Time (years)",  ylab = "Survival Probability",
     lwd = 2, main = paste("GPS2*GPS2P1 Expression and Survival"))
leg.txt <- c("Below median", "Above median")
legend("topright", leg.txt, lty = 1, col = 1:2, bty = "n", lwd = 2, title =
"GPS2*GPS2P1 Expression")
```

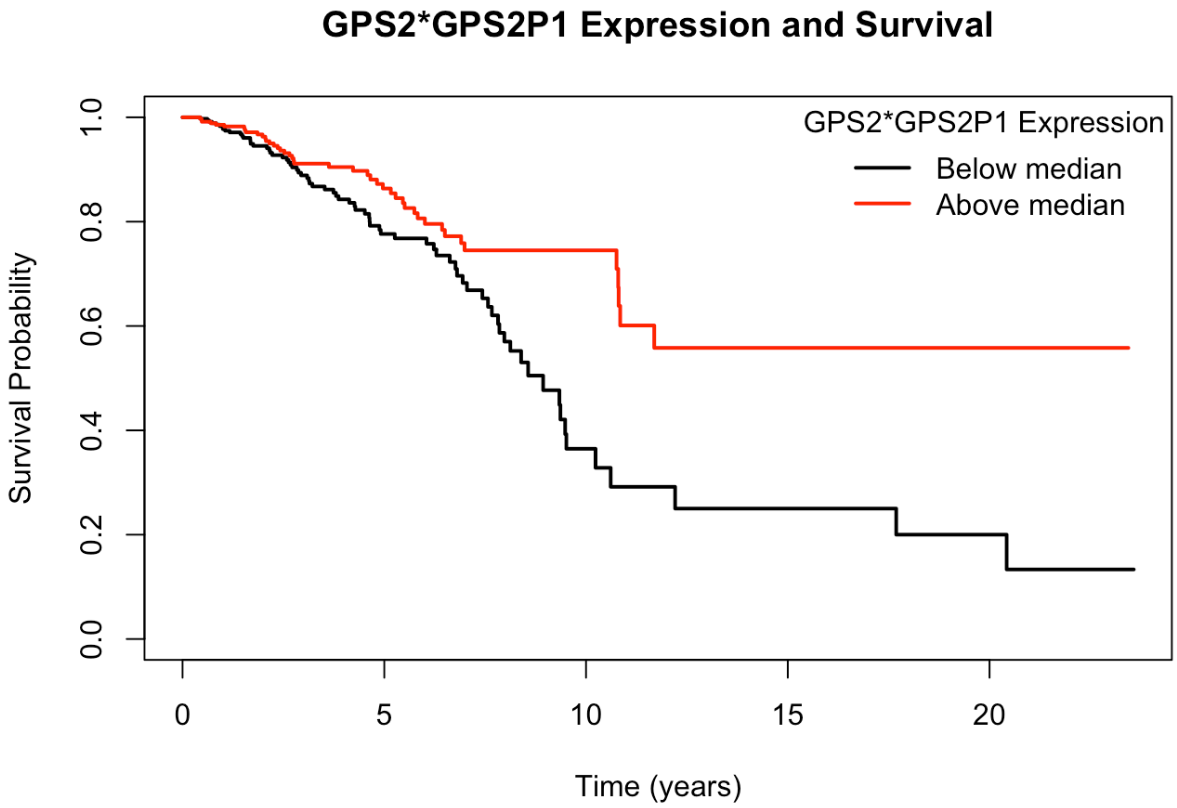


```
## Split at median expression of HSPA8-HSPA8P1 interaction
# cox.TRIM21TRIM60P17 <- coxph(Surv(survival.years, vital_status) ~
RNA.g.pg.counts.01[,"TRIM21*TRIM60P17"], data = surdata4)
# summary(cox.TRIM21TRIM60P17)
#
# TRIM21TRIM60P17.grps <- ifelse(RNA.g.pg.counts.01[,"TRIM21*TRIM60P17"] >
median(RNA.g.pg.counts.01[,"TRIM21*TRIM60P17"]), 1, 0)
# table(TRIM21TRIM60P17.grps)
#
# km.TRIM21TRIM60P17 <- survfit(Surv(survival.years, vital_status) ~
TRIM21TRIM60P17.grps, data = surdata4)
# plot(km.TRIM21TRIM60P17, col = 1:2, xlab = "Time (years)",  ylab = "Survival
Probability",
#      lwd = 2, main = paste("TRIM21*TRIM60P17 Expression and Survival"))
# leg.txt <- c("Below median", "Above median")
# legend("topright", leg.txt, lty = 1, col = 1:2, bty = "n", lwd = 2, title =
"TRIM21*TRIM60P17 Expression")
```

```
## Split at median expression of SLC25A47-SLC25A20P1 interaction
# cox.SLC25A47SLC25A20P1 <- coxph(Surv(survival.years, vital_status) ~
RNA.g.pg.counts.01[,"HSPA8*HSPA8P1"], data = surdata4)
# summary(cox.SLC25A47SLC25A20P1)
#
# SLC25A47SLC25A20P1.grps <- ifelse(RNA.g.pg.counts.01[,"SLC25A47*SLC25A20P1"] >
median(RNA.g.pg.counts.01[,"SLC25A47*SLC25A20P1"]), 1, 0)
# table(SLC25A47SLC25A20P1.grps)
#
# km.SLC25A47SLC25A20P1 <- survfit(Surv(survival.years, vital_status) ~
SLC25A47SLC25A20P1.grps, data = surdata4)
# plot(km.SLC25A47SLC25A20P1, col = 1:2, xlab = "Time (years)",  ylab = "Survival
Probability",
#       lwd = 2, main = paste("SLC25A47*SLC25A20P1 Expression and Survival"))
# leg.txt <- c("Below median", "Above median")
# legend("topright", leg.txt, lty = 1, col = 1:2, bty = "n", lwd = 2, title =
"SLC25A47*SLC25A20P1 Expression")

# More junk
xGPS2 = RNA.g.pg.counts.01[,"GPS2"]
xGPS2 = xGPS2[surdata4$vital_status==1]
xGPS2P1 = RNA.g.pg.counts.01[,"GPS2P1"]
xGPS2P1 = xGPS2P1[surdata4$vital_status==1]
ySurYrs = surdata4$survival.years[surdata4$vital_status==1];
lm(log2(ySurYrs[xGPS2P1<0.1]+1)~log2(xGPS2[xGPS2P1<0.1]+1))
##
## Call:
## lm(formula = log2(ySurYrs[xGPS2P1 < 0.1] + 1) ~ log2(xGPS2[xGPS2P1 <
##     0.1] + 1))
##
## Coefficients:
##                    (Intercept)  log2(xGPS2[xGPS2P1 < 0.1] + 1)
##                         3.6635                         -0.5919
lm(log2(ySurYrs[xGPS2P1>0.1]+1)~log2(xGPS2[xGPS2P1>0.1]+1))
##
## Call:
## lm(formula = log2(ySurYrs[xGPS2P1 > 0.1] + 1) ~ log2(xGPS2[xGPS2P1 >
##     0.1] + 1))
##
## Coefficients:
##                    (Intercept)  log2(xGPS2[xGPS2P1 > 0.1] + 1)
##                        -0.4773                          1.1450
lm(log2(ySurYrs+1)~log2(xGPS2+1))
##
## Call:
## lm(formula = log2(ySurYrs + 1) ~ log2(xGPS2 + 1))
##
## Coefficients:
##     (Intercept)  log2(xGPS2 + 1)
##          1.1405           0.4714
plot(log2(ySurYrs+1)~log2(xGPS2+1), col=ifelse(xGPS2P1<0.1,"red","blue"))
abline(1.1405,.4714)
abline(3.6635,-0.5919,col="red")
abline(-0.4773,1.1450,col="blue")
```

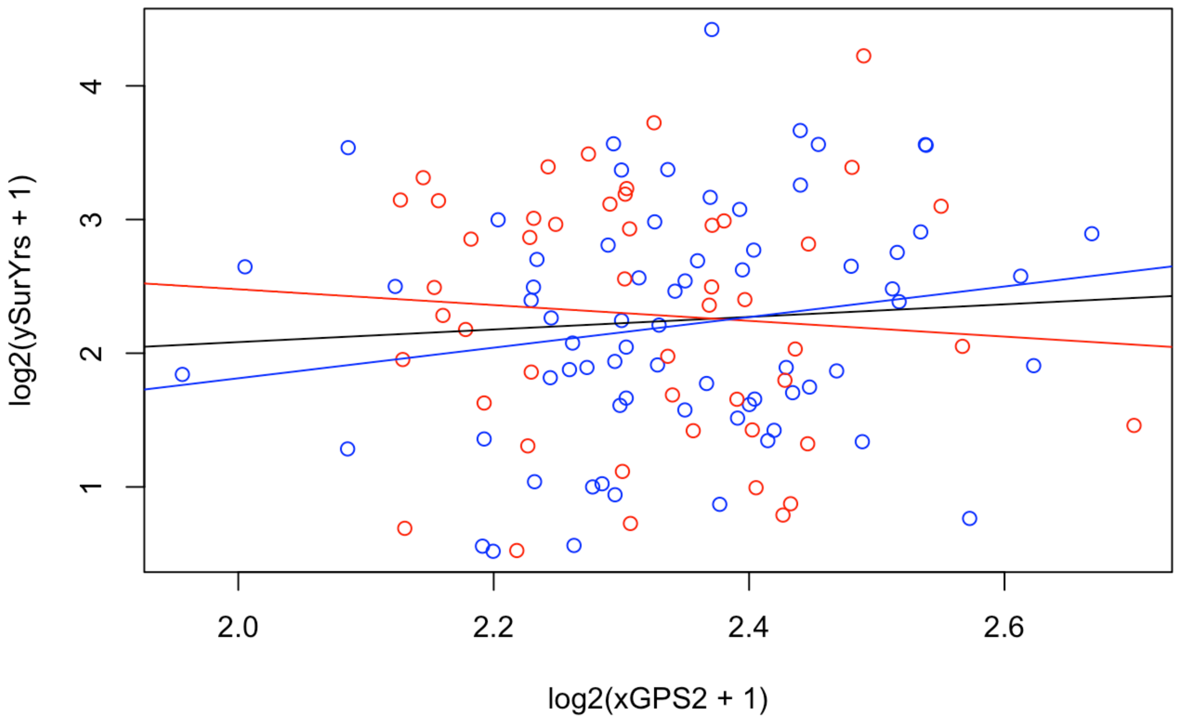


```
# xTRIM21 = RNA.g.pg.counts.01[,"TRIM21"]
# xTRIM21 = xTRIM21[surdata4$vital_status==1]
# xTRIM60P17 = RNA.g.pg.counts.01[,"TRIM60P17"]
# xTRIM60P17 = xTRIM60P17[surdata4$vital_status==1]
# lm(log2(ySurYrs[xTRIM60P17<0.3]+1)~log2(xTRIM21[xTRIM60P17<0.3]+1))
# lm(log2(ySurYrs[xTRIM60P17>0.3]+1)~log2(xTRIM21[xTRIM60P17>0.3]+1))
# lm(log2(ySurYrs+1)~log2(xTRIM21+1))
# plot(log2(ySurYrs+1)~log2(xTRIM21+1), col=ifelse(xTRIM60P17<0.3,"red","blue"))
# abline(0.02116,1.08163)
# abline(0.2326,0.9658,col="red")
# abline(-4.593,3.745,col="blue")

cor.test(RNA.g.pg.counts.01[RNA.g.pg.counts.01[,"GPS2P1"]<0.1,"GPS2P1"],RNA.g.pg.count
s.01[RNA.g.pg.counts.01[,"GPS2P1"]<0.1,"GPS2"])
##
##  Pearson's product-moment correlation
##
## data:  RNA.g.pg.counts.01[RNA.g.pg.counts.01[, "GPS2P1"] < 0.1, "GPS2P1"] and
RNA.g.pg.counts.01[RNA.g.pg.counts.01[, "GPS2P1"] < 0.1, "GPS2"]
## t = -3.2164, df = 222, p-value = 0.001492
## alternative hypothesis: true correlation is not equal to 0
## 95 percent confidence interval:
##  -0.33288586 -0.08220147
## sample estimates:
##        cor
## -0.2110106
cor.test(RNA.g.pg.counts.01[RNA.g.pg.counts.01[,"GPS2P1"]>0.1,"GPS2P1"],RNA.g.pg.count
s.01[RNA.g.pg.counts.01[,"GPS2P1"]>0.1,"GPS2"])
##
##  Pearson's product-moment correlation
##
```

```
## data:  RNA.g.pg.counts.01[RNA.g.pg.counts.01[, "GPS2P1"] > 0.1, "GPS2P1"] and
RNA.g.pg.counts.01[RNA.g.pg.counts.01[, "GPS2P1"] > 0.1, "GPS2"]
## t = 10.749, df = 536, p-value < 2.2e-16
## alternative hypothesis: true correlation is not equal to 0
## 95 percent confidence interval:
##  0.3489863 0.4882517
## sample estimates:
##       cor
## 0.4210977
cor.test(RNA.g.pg.counts.01[,"GPS2P1"],RNA.g.pg.counts.01[,"GPS2"])
##
##  Pearson's product-moment correlation
##
## data:  RNA.g.pg.counts.01[, "GPS2P1"] and RNA.g.pg.counts.01[, "GPS2"]
## t = 7.99, df = 760, p-value = 5e-15
## alternative hypothesis: true correlation is not equal to 0
## 95 percent confidence interval:
##  0.2115331 0.3426218
## sample estimates:
##       cor
## 0.2783733
#
cor.test(RNA.g.pg.counts.01[RNA.g.pg.counts.01[,"TRIM60P17"]<0.2,"TRIM60P17"],RNA.g.pg
.counts.01[RNA.g.pg.counts.01[,"TRIM60P17"]<0.2,"TRIM21"])
#
cor.test(RNA.g.pg.counts.01[RNA.g.pg.counts.01[,"TRIM60P17"]>0.2,"TRIM60P17"],RNA.g.pg
.counts.01[RNA.g.pg.counts.01[,"TRIM60P17"]>0.2,"TRIM21"])
# cor.test(RNA.g.pg.counts.01[,"TRIM60P17"],RNA.g.pg.counts.01[,"TRIM21"])
# paired.r(0.5203,-0.3540,NULL,725,33)
# paired.r(0.4211,-0.2110,NULL,536,222)
```
